# PRICKLE3 protects VANGL proteins from CK1-mediated phosphorylation and RNF43-mediated degradation

**DOI:** 10.1038/s42003-025-09422-9

**Published:** 2025-12-27

**Authors:** Katarzyna A. Radaszkiewicz, Tomasz W. Radaszkiewicz, Pavla Kolářová, Petra Paclíková, Kristína Gömöryová, Šárka Novotná, Lorena Agostini Maia, Tereza Číhalová, Yao Le, Tomáš Bárta, Kateřina Hanáková, Anna Hýsková, Konstantinos Tripsianes, Zbyněk Zdráhal, Christoph Winkler, Jakub Harnoš

**Affiliations:** 1 Department of Experimental Biology, Faculty of Science, Masaryk University, Brno, Czechia; 2 Department of Biological Sciences and Centre for Bioimaging Sciences, National University of Singapore, Singapore, Singapore; 3 Department of Histology and Embryology, Faculty of Medicine, Masaryk University, Brno, Czechia; 4 CEITEC-Central European Institute of Technology, Masaryk University, Brno, Czechia; 5 National Centre for Biomolecular Research, Faculty of Science, Masaryk University, Brno, Czechia

**Keywords:** Cell signalling, Post-translational modifications

## Abstract

The PRICKLE proteins (PRICKLE1–PRICKLE4) play essential roles in the WNT/planar cell polarity (WNT/PCP) pathway in vertebrates. This signaling system governs cell polarity, tissue architecture, and coordinated cell movements, yet the specific roles and molecular mechanisms of individual PRICKLE members within this pathway are poorly understood. Here, we identify PRICKLE3 as a previously unrecognized, central regulator of WNT/PCP signaling in human cells, *Xenopus laevis* and zebrafish (*Danio rerio*) embryos. Using enhanced proximity biotinylation (miniTurboID) combined with mass spectrometry, we found PRICKLE3 enriched at the plasma membrane, where it associates with core WNT/PCP proteins, including VANGL1 and VANGL2. Through immunoblotting, live imaging and functional assays, we further demonstrated that PRICKLE3 selectively enhances VANGL1/2 stability by protecting them from Casein kinase 1ε (CK1ε)-mediated phosphorylation. Mechanistically, PRICKLE3 modulates an interaction network involving VANGL1/2, CK1ε, and the ubiquitin ligase RNF43, thereby increasing VANGL stabilization and accumulation at the plasma membrane. These effects were unique to PRICKLE3, as PRICKLE1 showed no comparable activity. Together, our findings reveal a PRICKLE3-specific mechanism that couples CK1ε inhibition with RNF43 suppression to stabilize VANGL complexes. We also provide a comprehensive interactome and molecular tools to support further functional dissection of the PRICKLE family in development and disease.

## Abstract (web summary)

We mapped PRICKLE1–3 interactomes and identified PRICKLE3 as a key WNT/PCP regulator that stabilizes VANGL via CK1ε–RNF43 control, revealing a PRICKLE3-specific mechanism shaping polarity across vertebrates.

## Introduction

The planar cell polarity (PCP) pathway is the most extensively studied branch of non-canonical, β-catenin-independent WNT signaling^1,2^. A hallmark of the PCP pathway is its ability to propagate polarized information between neighboring cells, ultimately coordinating the global orientation of tissues within the body plane^3–5^. This foundational PCP signaling concept was first established through pioneering genetic studies in *Drosophila melanogaster*, where a set of key core PCP components were identified^3,6–9^. In vertebrates, the PCP pathway not only governs cellular polarity within (epithelial) tissues but also orchestrates essential processes such as collective cell migration, individual cell motility, and axon guidance^7,10–12^. Disruptions in PCP signaling in vertebrates are associated with a variety of developmental defects, including neural tube closure failure and craniofacial malformations, and have also been linked to skeletal and neurological syndromes^3,13–15^. Beyond development, dysregulated PCP signaling has been associated with cancer progression, where it contributes to metastatic dissemination and resistance to therapy^16,17^.

At the molecular level, PCP signaling relies on asymmetric transmembrane and cytoplasmic protein assemblies that generate polarized signaling domains. Frizzled (FZD) receptors and the cytoplasmic scaffold protein Dishevelled (DVL) oppose complexes formed by the transmembrane protein Van Gogh-like (VANGL) and its cytosolic partner PRICKLE^7,9,18^. Flamingo, known in vertebrates as CELSR (Cadherin EGF LAG Seven-Pass G-Type Receptor), is another critical membrane-spanning PCP component that acts as an atypical cadherin mediating cell adhesion and PCP asymmetry across neighboring cells^19,20^. PCP-specific WNT ligands, particularly WNT5A and WNT11, activate receptor complexes that include additional co-receptors such as ROR1/2, RYK, and PTK7^1,2,7,9,21^. PCP signaling fidelity depends on the precise regulation of these complexes at the plasma membrane, including their assembly, turnover, and post-translational modification.

Among the core PCP components, the PRICKLE protein family (PRICKLE1–4) remains one of the least understood, especially in vertebrate systems. Although genetic studies in invertebrates have established Prickle proteins as essential FZD/DVL complex antagonists, their function appears largely passive during planar polarity-dependent cytoskeletal remodeling, such as trichome orientation in *Drosophila*^1,2^. In contrast, vertebrate PRICKLE proteins seem to play a more active role, contributing directly to actomyosin regulation and morphogenesis, as recently shown^22–26^. However, their molecular mechanisms of action, subcellular dynamics, and functional specificity in vertebrate PCP signaling remain largely unexplored^15,27^. Several studies have implicated protein stability, trafficking, and ubiquitin-mediated regulation as critical factors in PCP control^1,12,17,28,29^, yet how PRICKLE proteins contribute to these layers of regulation is unknown.

In this study, we employed enhanced proximity-dependent biotinylation coupled with mass spectrometry to map protein interaction networks of PRICKLE family members in human cells. We further combined in vivo imaging, immunoblotting, and functional analysis in human cells, *Xenopus laevis*, and zebrafish (*Danio rerio*) with proteomic approaches to dissect the regulation of VANGL stability by PRICKLE proteins. Our analysis identified PRICKLE3 as a highly connected component of WNT/PCP receptor complexes that stabilizes VANGL proteins and potentiates WNT5A signaling. We further demonstrate that PRICKLE3 regulates the activity of the E3 ubiquitin ligase RNF43, a clinically relevant modulator of WNT receptor turnover. Together, these findings define a PRICKLE3-specific mechanism that safeguards VANGL complexes from degradation and offer molecular tools to dissect the functions of PRICKLE genes across vertebrate models using biochemical and proteomic approaches.

## Results

### PRICKLE3 interactome uncovers specific association with WNT/PCP pathway proteins

**Fig. 1:**
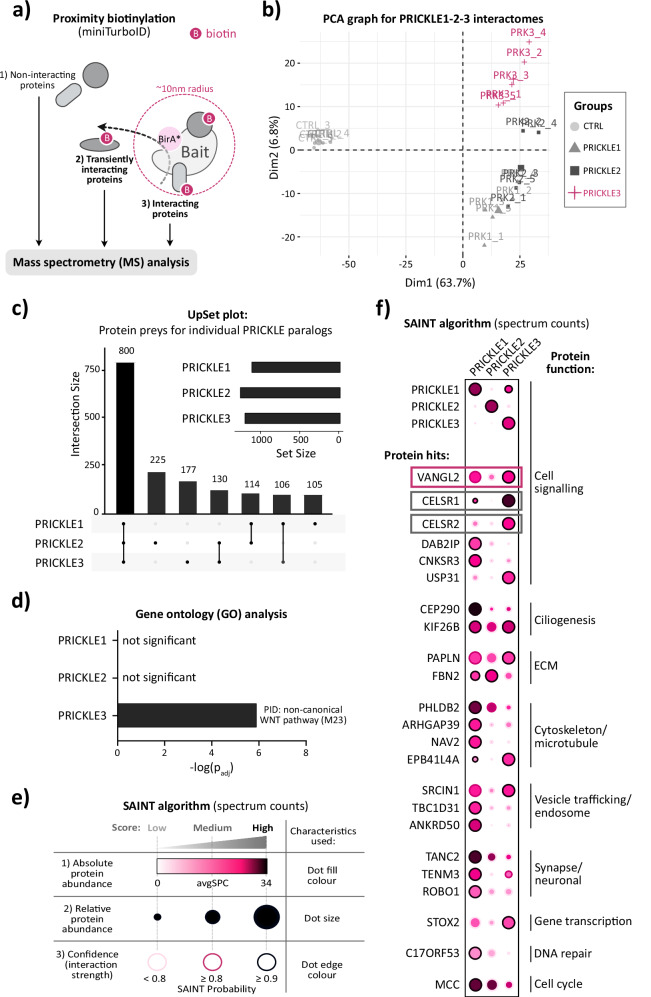
PRICKLE3 interactome uncovers specific association with WNT/PCP pathway proteins. **a** Schematic representation of the miniTurboID proximity-dependent biotinylation assay. The method involves biotin labeling of proteins in close proximity to the engineered biotin ligase (BirA*) fused with a protein of interest, followed by streptavidin-based enrichment and mass spectrometry analysis. Biotinylated proteins include both direct interactors and transiently proximal proteins, while non-interacting proteins lacking biotinylation were excluded from subsequent bioinformatic analyses. **b** Principal Component Analysis (PCA) showing sample clustering based on PRICKLE1–3 paralogs. Replicates for each bait clustered tightly together, indicating high reproducibility. PRICKLE1 and PRICKLE2 displayed similar prey profiles, whereas PRICKLE3 revealed a distinct interactome. **c** UpSet plot illustrating the overlap of biotinylated prey proteins among PRICKLE1–3 paralogs, providing insight into shared and paralog-specific interactors. **d** Gene Ontology (GO) enrichment analysis using Metascape revealed that only the interactors of PRICKLE3 were significantly enriched in components of the non-canonical WNT/Planar Cell Polarity (PCP) pathway (PID M23), indicating functional divergence between paralogs. **e**, **f** Dot plot with a legend summarizing the SAINTexpress (Significance Analysis of INTeractome) results, assessing bait–prey interaction confidence across clusters. In the dot plot, the color of each dot reflects the absolute abundance of the prey protein, with darker shades indicating higher abundance. The size of the dot represents the relative abundance of the prey across samples. The edge color of each dot encodes the SAINT Probability (SP), with darker edges corresponding to stronger bait–prey interactions.

Proximity-dependent labeling techniques have emerged as a well-established method to map protein–protein interactions^30,31^. To characterize PRICKLE interactomes, we used enhanced proximity-dependent biotinylation (miniTurboID), which employs a modified biotin ligase (BirA*) to label stable and transient interactors within ~10 nm of the protein of interest (POI, bait) (Fig. 1a)^32^. We developed stable HEK T-REx 293 TetON cell lines that inducibly express the PRICKLE1-3 proteins fused N-terminally with modified BirA* ligase (miniTurboID) and the V5 tag. PRICKLE4 was not analysed because it lacks the canonical three LIM domains present in the other members^15^. As a negative control, we used cells expressing only the miniTurboID-V5 construct, ensuring that any biotinylation observed was specific to PRICKLE fusion proteins (Fig. S1a). We validated the specificity of biotinylation by Westen blotting (WB) and immunofluorescence, confirming that interacting proteins were biotinylated only in the presence of doxycycline (DOX) and biotin (Fig. S1b–d). To ensure the data’s robustness, we prepared five independent biological replicates for each POI. The biotinylated proteins were enriched using streptavidin-coated beads and subsequently identified through mass spectrometry (MS). In total, MS identified a set of 7011 proteins, which, after quality filtering, was reduced to 5986 proteins (Supplementary Data 1). Subsequently, principal component analysis (PCA) of the 500 most variable proteins revealed cohesive replicate clustering and excluded the batch effect (Fig. 1b). Notably, it showed that PRICKLE3 has the most distinct interactome, whereas PRICKLE1 and PRICKLE2 share similar interactors (Fig. 1b).

All three PRICKLE variants had a similar number of significant proximal interactors, around 1100 (Fig. S1e, Supplementary Data 2), of which approximately 800 were common (Fig. 1c). PRICKLE2 had the highest number of unique interactors (225), followed by PRICKLE3 (177) and PRICKLE1 (105). Pairwise, PRICKLE3 and PRICKLE2 shared the most candidate interactors (130), while PRICKLE3 and PRICKLE1 shared 106 preys (Fig. 1c, Supplementary Data 3). Notably, PRICKLE1 and PRICKLE2 shared only 114 preys (Fig. 1c, Supplementary Data 3), which is surprisingly low given their closer sequence similarity^15^. This implies a high level of redundancy between PRICKLE members together with some degree of specialization of the individual paralogs.

To investigate potential functional differences between the paralogs, we first used the Human Cell Map (HCM)^33^ to precisely determine the subcellular localizations of PRICKLE1-3 based on their interactors. A great advantage of using this resource is that it was developed with the BioID assay in the same cellular model as our study (i.e., T-REx 293 cells). However, it is worth noting that not all proteins identified in this study could be mapped to the HCM, likely due to the use of a more robust biotin ligase miniTurboID. Our HCM analysis indicated that all three PRICKLE paralogs were predominantly located in cell junctions, mitochondrial matrix and plasma membrane. (Fig. S1f, Supplementary Data 4).

Then, we performed GO (Gene ontology) analysis^34^ on proteins identified as specific interactors of individual PRICKLE proteins. We observed that PRICKLE3 is most strongly connected with the non-canonical WNT signaling pathway (Fig. 1d, Supplementary Data 5–8). Furthermore, *k*-means clustering of candidate interactors uncovered two PRICKLE3-associated clusters composed of established WNT/PCP components, including VANGL1/2 and CELSR1/2 (Fig. S1g–h). The GO analysis also showed that the interactome of PRICKLE1-3 is enriched with proteins associated with membrane organization and cytoskeleton, as well as regulation of metabolism and other processes (Fig. S1i). Moreover, this analysis revealed that all three PRICKLE proteins are involved in various signaling pathways, especially signaling by vascular endothelial growth factor (VEGF), Rho GTPases, and WNT signaling pathway (Fig. S1i).

A more detailed analysis, involving the plotting of data as log_2_ fold change (log_2_ FC) against -log10 (adjusted p-value, padj), provided additional resolution (Fig. S1j). This approach suggests that a bait protein is likely to remain longer and result in stronger biotinylation of prey proteins, or alternatively, indicates a higher concentration of the bait protein within a particular cellular compartment. This analysis showed a stronger association of PRICKLE3 with integral plasma membrane non-canonical WNT components than PRICKLE1, as demonstrated by PCP proteins such as ROR1/2 and CELSR1/2, and it also indicates a stronger association of PRICKLE3 with VANGL1 and VANGL2 (Fig. S1j).

As a complementary approach to determining the confident bait-prey interactions using log_2_FC and adjusted *p*-values threshold on limma test results, we employed the SAINTexpress (Significance Analysis of INTeractome) tool^35^, which was integrated within the REPRINT resource^36^. SAINTexpress provides the SAINT probability (SP) metric, where SP > 0.9 means a highly confident interaction. In our data, we identified 26 bait-prey pairs visualized by DotPlot using SP > 0.9 (Fig. 1e, f, Supplementary Data 9). This analysis further supports that the PRICKLE3 paralog is the most significantly related to the non-canonical WNT components such as VANGL2, and CELSR1/2 (Fig. 1f).

Since our data indicated a strong link between PRICKLE3 and the non-canonical WNT pathway, together with an unexpected association with VANGL proteins, we decided to investigate this interaction in more detail in subsequent functional studies.

### PRICKLE3 contains functional VANGL-binding motifs (VBMs) and interacts with VANGL1/2

To further investigate the interaction between PRICKLE3 and VANGL1/2, we revisited the long-standing assumption that PRICKLE3 lacks a VANGL-binding domain (VBD), as initially suggested previously^37^. All PRICKLE (1–3) family members share three conserved sequence elements: the N-terminal PET (Prickle, Espinas, Testin) domain involved in protein–protein interactions and signal transduction; a cluster of three LIM (Lin-11, Isl-1, Mec-3) domains, which are Cys-rich motifs with diverse cellular functions; and a C-terminal PKH (Prickle homology) domain with a less well-defined role. In addition, all PRICKLE proteins possess a central intrinsically disordered region (IDR) that varies in length and sequence among paralogs^15^.

**Fig. 2:**
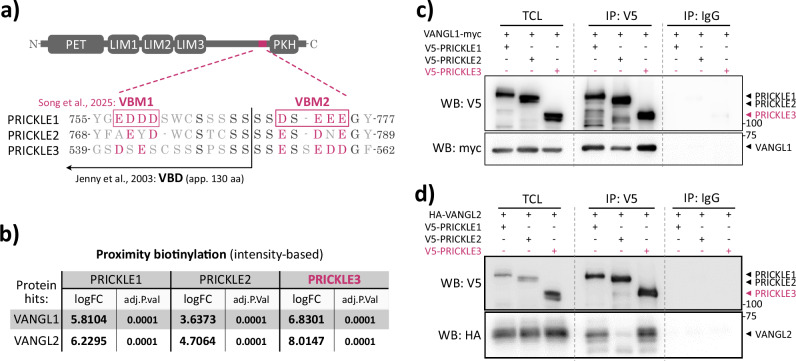
PRICKLE3 contains functional VANGL-binding motifs (VBMs) and interacts with VANGL1/2. **a** Multiple sequence alignment of PRICKLE1–3 in the region corresponding to the VANGL-binding domain (VBD) and two VANGL-binding motifs (VBMs). The alignment reveals that PRICKLE3 also contains conserved regions. Amino acid positions are indicated by numbers. The schematic above illustrates the domain organization of PRICKLE proteins, including the PET domain, three LIM domains, a central intrinsically disordered region, and the Prickle homology (PKH) domain. **b** Table summarizing proximity biotinylation results for VANGL1 and VANGL2 proteins, showing log₂ fold change (log₂FC) and –log₁₀(adjusted *p*-value). Values with log₂FC > 1.00 and *p* < 0.05 are highlighted in bold. **c** Co-immunoprecipitation of overexpressed VANGL1-myc with V5-tagged PRICKLE1–3 from HEK293 cells. Immunoprecipitation (IP) was performed using anti-V5 to pull down V5-PRICKLE proteins. PRICKLE3 showed strong interaction with VANGL1. TCL total cell lysate. **d** Co-immunoprecipitation of overexpressed VANGL2-HA with V5-tagged PRICKLE1–3 from HEK293 cells. Immunoprecipitation was performed using the anti-V5 antibody. PRICKLE3 exhibited strong association with VANGL2. TCL total cell lysate.

Recent cryo-EM studies^38,39^ have redefined the original VBD proposed previously^37^ (~130 amino acids near the C-terminus of PRICKLE) into two shorter VANGL-binding motifs (VBMs) enriched in negatively charged residues—one motif located within the previously proposed VBD and the other adjacent to it, closer to the C-terminus. By generating a short multiple sequence alignment (Fig. 2a), we identified that both negatively charged regions are conserved in PRICKLE3 to certain extent, supporting the presence of functional binding interface with VANGL.

Our proximity-biotinylation data, based on interaction signal intensity, further showed that PRICKLE3 interacts with both VANGL1 and VANGL2 at comparable levels to PRICKLE1 and PRICKLE2 (Fig. 2b). To experimentally validate this hypothesis as well as to confirm our proximity-based interactome data, we performed co-immunoprecipitation (co-IP) assays using all three PRICKLE paralogs. Consistent with our bioinformatic analyses (Fig. S1j, Fig. 1f) and sequence conservancy prediction (Fig. 2a), PRICKLE3 physically interacted with VANGL1 (Fig. 2c, Fig. S2a-b) and also bound VANGL2 (Fig. 2d, Fig. S2c-d). These results provide the first experimental evidence that PRICKLE3 contains a functional VANGL-binding region (i.e., two VBMs), reinforcing its direct involvement in the WNT/PCP signaling axis. Since VANGL1 and VANGL2 behaved essentially identically in our binding assays, we consider them functionally similar in the context of PRICKLE interactions.

We next examined how PRICKLE paralogs interact with endogenous DVL2 and DVL3 (Fig. S2a, c). All PRICKLE proteins bound both DVL isoforms, but PRICKLE3 additionally caused a noticeable electrophoretic shift in DVL2 and DVL3. Consistent with this, PRICKLE3 also interacted with DVL1 and DVL3 in our proximity assays (Fig. S2e). In summary, these results demonstrate that human PRICKLE proteins can bind DVL, although a detailed characterization of these interactions was beyond the scope of this study.

#### VANGL antibodies validation

Given that VANGL1/2 emerged as prominent PRICKLE3 interactors in both our proximity interactome analyses and co-IP experiments, we next validated a set of VANGL antibodies used for following experimental assays. The VANGL N-terminal region contains a cluster of Ser/Thr residues (Thr78, Ser79, and Ser82 in human VANGL2; Fig. S2f), which are known CK1δ/ε phosphorylation targets; phosphorylation by CK1δ/ε is required to activate VANGL and its trafficking to the plasma membrane^40–44^. We confirmed that phosphorylation of VANGL1/2 is CK1ε-dependent in our model: treatment of cells with the CK1δ/ε inhibitor PF-670462 (ref. ^45^) decreased phosphorylation of endogenous VANGL and accelerated the VANGL1 electrophoretic mobility on SDS-PAGE/WB (Fig. S2g). Consistently, CK1ε overexpression further increased phosphorylation of both VANGL1 and VANGL2 (Fig. S2h). In our cell-based model, VANGL phosphorylation was dependent on R-spondin, the RNF43 ubiquitin ligase inhibitor, but not on WNT5A ligands (Fig. S2g). To further assess the specificity of VANGL antibodies, we used *VANGL2* knockout (KO) and *VANGL1/2* double KO cells^46^. The antibody predominantly detected phosphorylated VANGL1, as evidenced by VANGL1^−/−^/2^−/^^−^ cells (Fig. S2g), but also phosphorylated VANGL2, as evidenced by the stronger signal observed in overexpressed VANGL2 with CK1ε (Fig. S2h). Based on these results, we designated the antibody as pVANGL1/2 and considered it a reliable readout for subsequent analyses.

### PRICKLE3 enhances VANGL protein stability and promotes ROR2 electrophoretic shifts

**Fig. 3:**
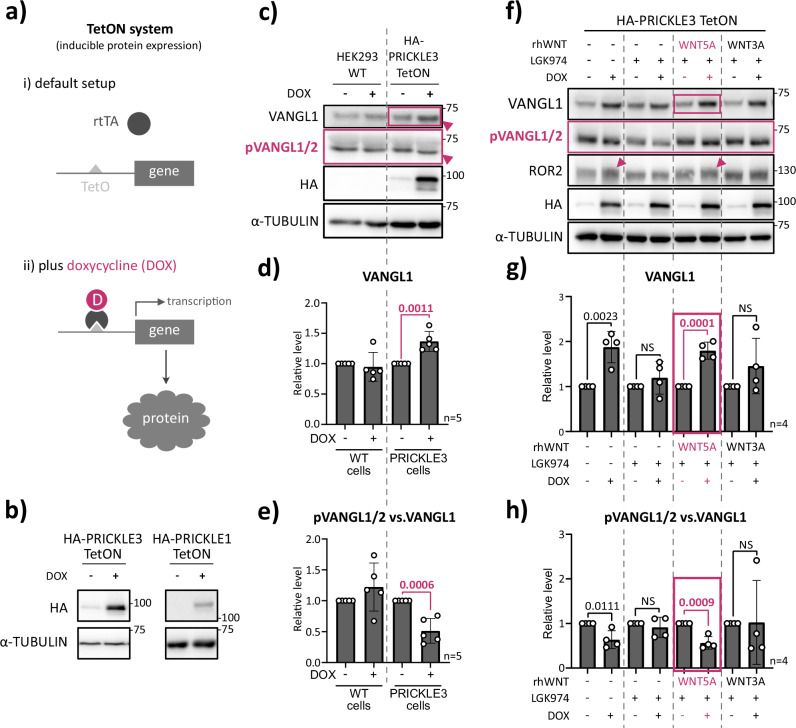
PRICKLE3 affects VANGL protein stability and correlates with changes in VANGL and ROR2 phosphorylation. **a** General schematic of the TetON inducible gene expression system used in this study. In the absence of doxycycline, the reverse tetracycline-controlled transactivator (rtTA) is inactive. Upon doxycycline addition, rtTA binds to the tetO promoter and activates transcription of the gene of interest, leading to protein expression. **b** Doxycycline-induced overexpression of HA-tagged PRICKLE3 in HEK T-REx 293 PRICKLE3 TetON cells and of PRICKLE1 in the corresponding inducible cell line. **c** Induction of HA-PRICKLE3 expression by doxycycline in HEK T-REx 293 PRICKLE3 TetON cells. HEK T-REx 293 wildtype (WT) cells served as a control for doxycycline effects. Arrowheads indicate phosphorylation-dependent shifts in the electrophoretic mobility of VANGL proteins. α-TUBULIN was used as a loading control. Representative result from *n* = 5. **d**, **e** Densitometric quantification of Western blot signals. Values were normalized to untreated cells. Statistical analysis was performed using an unpaired *t*-test; corresponding *p*-values are shown (*n* = 5). **f** Effect of recombinant WNT stimulation on VANGL phosphorylation. HEK T-REx 293 PRICKLE3 TetON cells were pre-treated overnight with the porcupine inhibitor LGK-974 to block endogenous WNT ligand secretion and subsequently stimulated with 100 ng/ml human recombinant WNT5A or WNT3A for 3 h. Arrowheads indicate phosphorylation-dependent mobility shifts of ROR. α-TUBULIN served as a loading control. Representative result from *n* = 4. **g**, **h** Densitometric quantification of Western blot signals from panel f. Data were normalized to untreated controls. Statistical analysis was performed using an unpaired *t*-test; corresponding *p*-values are reported (*n* = 4).

HEK293 cells can be used to study individual components and molecular mechanisms of both canonical and non-canonical WNT pathways^47–53^. For mechanistic studies, we exploited a DOX-inducible Tet-ON system in HEK293 T-REx cells (Fig. 3a). This system avoids transfection-related stress and thus offers more physiological conditions than transient overexpression. Specifically, we generated stable HEK293 T-REx cell lines inducibly expressing HA-tagged PRICKLE1 and PRICKLE3 under a DOX control (Fig. 3b; Fig. S3a–e).

Previous studies have demonstrated a reciprocal regulation between PRICKLE and VANGL proteins, with both shown to modulate the stability of each other and influence the WNT pathway^43,54–59^. Based on these insights, our initial objective was to evaluate whether PRICKLE3 alters VANGL protein abundance and phosphorylation status. PRICKLE3 induced expression significantly elevated VANGL1 levels and caused a shift in its electrophoretic mobility on SDS-PAGE (Fig. 3c–e), indicating reduced phosphorylation. This finding was independently confirmed using the pVANGL1/2 antibody, which showed a concomitant reduction in signal and a mobility shift (Fig. 3c, Fig. S3g). To quantify this effect, we calculated the ratio of phosphorylated VANGL1/2 to total VANGL1, revealing a statistically significant decrease (Fig. 3e). This demonstrates that PRICKLE3 not only stabilizes VANGL1 but also modulates its phosphorylation. To test whether this mechanism is cell-type specific, we replicated the experiment in MDCK cells, where we observed a comparable increase in VANGL1 levels (Fig. S3h–i), supporting the conserved and robust nature of this regulation.

Notably, PRICKLE3 had no detectable effect on DVL2/3 phosphorylation or total protein levels in our inducible system (Fig. S3f; see Discussion for further interpretation).

We next examined how PRICKLE3 modulates VANGL levels and its phosphorylation in the presence of WNT ligands and pathway inhibitors (Fig. 3f–h, Fig. S3j–k). Under endogenous WNT blockage conditions using the porcupine inhibitor LGK-974, PRICKLE3 had no effect on VANGL1 or pVANGL2/VANGL1 ratios, suggesting that its function is ligand-dependent. Strikingly, PRICKLE3-mediated effects on VANGL phosphorylation and levels were observed exclusively in cells treated with WNT5A, a non-canonical WNT ligand, but not with WNT3A, which activates the canonical pathway (Fig. 3f–h). Moreover, PRICKLE3 expression induced a electrophoretic shift in ROR2 (pink arrowheads in Fig. 3f), a known WNT5A co-receptor phosphorylated upon ligand binding^60,61^. ROR2 has been shown to form a WNT/PCP receptor complex with VANGL2 in response to WNT5A stimulation^41^, underscoring the potential of PRICKLE3 to regulate membrane-localized WNT/PCP complexes. Regarding DVL2/3, PRICKLE3 did not alter their level and phosphorylation status (Fig. S3j), suggesting that DVL2/3 operate in different WNT complexes.

Collectively, these data indicate that PRICKLE3 affects VANGL protein stability and correlates with changes in VANGL and ROR2 co-receptor phosphorylation.

### PRICKLE3, but not PRICKLE1, stabilizes VANGL1/2 by protecting it from degradation

**Fig. 4:**
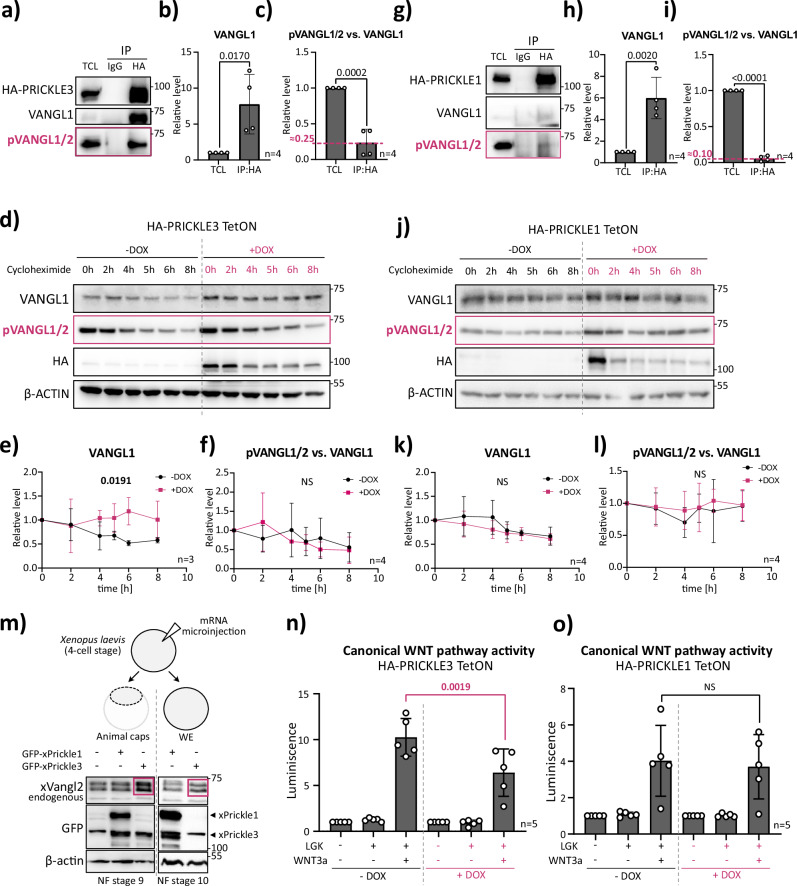
PRICKLE3, not PRICKLE1, stabilizes VANGL by protecting it from degradation. **a** Co-immunoprecipitation of HA-tagged PRICKLE3. HEK T-REx 293 PRICKLE3 TetON cells were induced overnight with doxycycline. Immunoprecipitation was performed using the anti-HA antibody or IgG control to assess the interaction between PRICKLE3 and endogenous VANGL1/2. *n* = 4. TCL = Total Cell Lysate. **b**, **c** Densitometric quantification of Western blot signals from panel a. Values were normalized to the corresponding TCL levels. Statistical analysis was performed using an unpaired *t*-test; corresponding *p*-values are reported (*n* = 4). **d** Cycloheximide (CHX) pulse-chase assay to assess PRICKLE3 protein stability. HEK T-REx 293 PRICKLE3 TetON cells (HA-PRICKLE3) were induced overnight with doxycycline, followed by treatment with 50 µg/mL CHX at various time points. Protein levels were analysed by Western blot; β-ACTIN served as a loading control. *n* = 3. **e**, **f** Densitometric quantification of Western blot signals from panel d. Results were normalized to the 0-h time point. Linear regression was used for statistical analysis; *p*-values are reported. (NS) indicates no statistically significant difference (in e, *n* = 3; in f, *n* = 4). **g** Co-immunoprecipitation of HA-tagged PRICKLE1. HEK T-REx 293 PRICKLE1 TetON cells were induced overnight with doxycycline. Immunoprecipitation using anti-HA antibodies or IgG control was performed to test for interaction with endogenous VANGL1/2. *n* = 4. TCL Total Cell Lysate. **h**, **i** Densitometric quantification of Western blot signals from panel **g**. Data were normalized to the corresponding TCL amount. Statistical analysis was performed using an unpaired *t*-test; *p*-values are reported (*n* = 4). **j** Cycloheximide (CHX) pulse-chase assay to assess PRICKLE1 protein stability. HEK T-REx 293 PRICKLE1 TetON cells (HA-PRICKLE1) were induced overnight with doxycycline, followed by treatment with 50 µg/mL CHX at indicated time points. Western blot analysis was performed with β-ACTIN as loading control. *n* = 4. **k**, **l** Densitometric quantification of Western blot signals. Values were normalized to the 0-hour time point. Statistical analysis was performed using linear regression. (NS) indicates no statistically significant difference (*n* = 4). **m** In vivo validation in *Xenopus laevis* embryos. Corresponding mRNAs were microinjected at the 4-cell stage, and animal caps were dissected at stage 9 and analysed by Western blot, together with whole emrbryos (WE) at stage 10. xPrickle3, not xPrickle1, expression led to stabilization of endogenous xVangl2. (x = *Xenopus*). **n**, **o** TOPFlash luciferase assay assessing canonical WNT/β-catenin pathway activity. In PRICKLE3-inducible cells (panel n), co-expression of PRICKLE3 led to decreased reporter activity, suggesting a potential stabilization of VANGL or inhibition of RNF43. In PRICKLE1-inducible cells (panel o), this effect was not observed. *n* = 5 independent experiments; statistical analysis was performed using unpaired Student’s *t*-test.

The increased VANGL protein levels observed upon inducible PRICKLE3 expression raised the possibility that PRICKLE3 either enhances VANGL synthesis or prevents its degradation. To distinguish between these possibilities and validate the interaction between PRICKLE3 and VANGL at the endogenous level, we first performed an immunoprecipitation assay in our inducible HEK293 cells. These experiments confirmed that endogenous VANGL, predominantly in the non-phosphorylated form, binds to PRICKLE3 (Fig. 4a–c, see also Fig. S4a for pVANGL detection).

To assess VANGL stability, we employed a cycloheximide (CHX)-based pulse-chase assay (Fig. 4d–f, see also Fig. S4b), a widely accepted method to measure protein degradation^62^. In the presence of PRICKLE3, VANGL levels remained stable over time (Fig. 4d, e), indicating that PRICKLE3 protects VANGL from degradation. In contrast, PRICKLE3 had no effect on the stability of phosphorylated VANGL1/2 (Fig. 4d; quantified in Fig. S4b) or DVL2 (Fig. S4c).

We next assessed the function of PRICKLE1. Immunoprecipitation of PRICKLE1 also pulled down VANGL, again mainly in its non-phosphorylated form (Fig. 4g–i, see also Fig. S4d for pVANGL detection). However, PRICKLE1 failed to stabilize either total or phosphorylated VANGL, as shown in the CHX assay (Fig. 4j–l, Fig. S4e-f).

This distinction between PRICKLE3 and PRICKLE1 supports the idea that PRICKLE3—but not PRICKLE1—stabilizes VANGL proteins. To further probe the mechanism, we turned to *Xenopus* embryos, a classical vertebrate model for PCP signaling^63^. Upon microinjection of mRNA encoding *Xenopus* Prickle1 or Prickle3, we observed elevated levels of endogenous Vangl2 only in the presence of xPrickle3—not xPrickle1—in both animal caps and whole embryos (WE), providing cross-species confirmation of the stabilizing effect in vivo and with the Vangl2 paralog (Fig. 4m).

To test whether VANGL stabilization has functional consequences, we employed a canonical WNT reporter assay (TOPFlash)^64^, based on the broadly accepted principle that canonical and non-canonical WNT signaling are often in antagonistic balance^2^. Stabilizing VANGL, a core component of the non-canonical pathway, should repress canonical WNT activity^41^. Consistent with this, PRICKLE3 induction led to a significant decrease in TOPFlash reporter activity (Fig. 4n), whereas PRICKLE1 expression had no such effect (Fig. 4o), in line with the ability of PRICKLE3 to stabilize VANGL1 or inhibit RNF43 (see further).

Together, these results establish PRICKLE3 as a functionally distinct paralog within the PRICKLE family, uniquely capable of stabilizing VANGL1/2 and modulating WNT pathway dynamics across vertebrate systems.

### PRICKLE3 reduces CK1-mediated phosphorylation of VANGL

The importance of Vangl/VANGL phosphorylation in establishing PCP has been confirmed in a range of animal models, including *Drosophila*, *Xenopus*, *Danio rerio*, and mouse^40,41,43,44,65^. VANGL phosphorylation by CK1δ/ε is essential for its activation, protection from endoplasmic-reticulum-associated degradation (ERAD), and trafficking to the plasma membrane^40^. Since our data demonstrate that PRICKLE3 reduces the levels of phosphorylated VANGL (Fig. 3c, quantified in Fig. S3g), and CK1ε is a known VANGL kinase, we next asked whether PRICKLE3 directly influences this phosphorylation step.

**Fig. 5:**
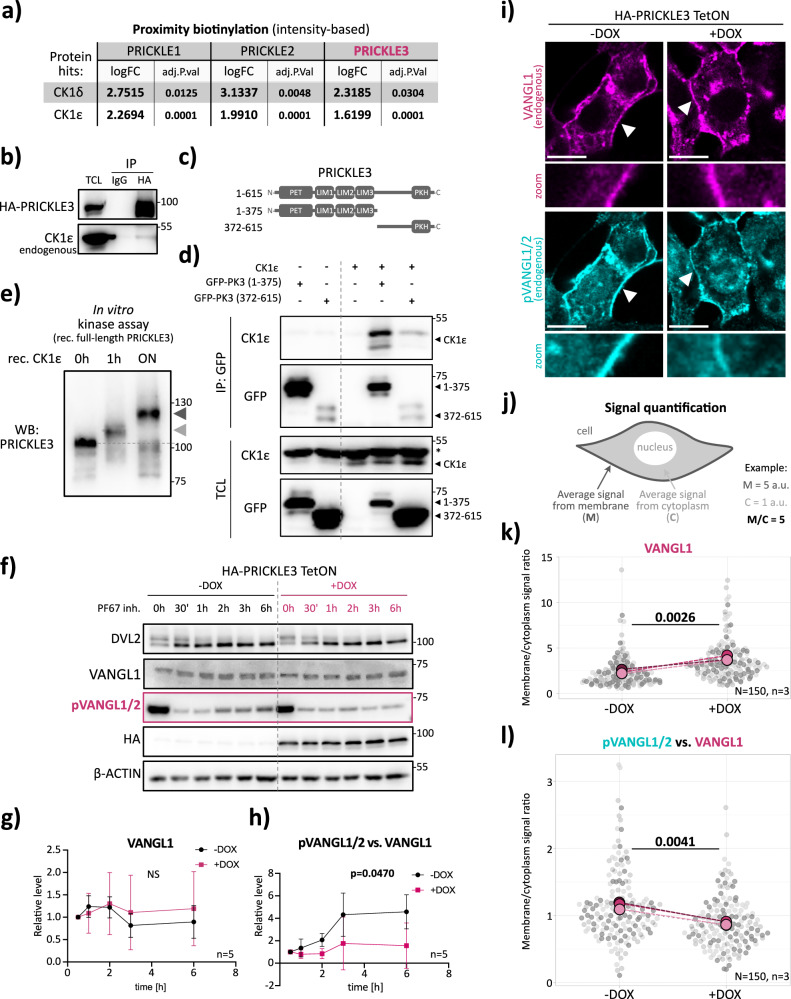
PRICKLE3 reduces CK1ε-mediated phosphorylation of VANGL. **a** Table summarizing proximity biotinylation results for CK1δ and CK1ε proteins, showing log₂ fold change (log₂FC) and –log₁₀(adjusted *p*-value). Values with log₂FC > 1.00 and *p* < 0.05 are highlighted in bold. **b** Co-immunoprecipitation of HA-tagged PRICKLE3. HEK T-REx 293 PRICKLE3 TetON cells were induced overnight with doxycycline. Pull-downs were performed using anti-HA antibodies and IgG control to test the interaction between PRICKLE3 and endogenous CK1ε. *n* = 4. TCL = Total Cell Lysate. **c** Schematic representation of PRICKLE3 deletion mutants: the N-terminal fragment (aa 1–375) and the C-terminal fragment (aa 372–615). **d** Co-immunoprecipitation of CK1ε with GFP-tagged PRICKLE3 deletion constructs shown in panel c. Immunoprecipitation was performed using anti-GFP antibody. Both fragments were able to bind CK1ε, but the N-terminal fragment exhibited a stronger interaction. **e** In vitro kinase assay using full-length PRICKLE3 as a substrate and recombinant CK1ε kinase. Reactions were performed at three time points (0-h, 1 h, and overnight – ON) in the presence of ATP (2 mM) and MgCl₂ (10 mM). Western blot analysis was used to detect PRICKLE3 phosphorylation using the anti-PRICKLE3 antibody. Gray triangles indicate phosphorylated PRICKLE3, while the dashed line marks the baseline corresponding to unphosphorylated PRICKLE3. **f** Analysis of VANGL phosphorylation dynamics by CK1ε in the presence or absence of PRICKLE3 in cells. HEK T-REx 293 PRICKLE3 TetON cells (HA-PRICKLE3) were induced overnight with doxycycline, followed by treatment with 2.5 μM PF-670462 (PF-67) at the indicated time points. Western blot was used to assess phosphorylation levels; β-ACTIN served as loading control. *n* = 5. **g**, **h** Densitometric quantification of Western blot signals from panel f. Results were normalized to the 0-h time point. Statistical analysis was performed using linear regression; corresponding *p*-values are reported (*n* = 5). **i** Immunofluorescence analysis of membrane-associated VANGL1 and pVANGL1/2 in the presence of PRICKLE3. PRICKLE3 expression was induced by overnight doxycycline treatment. Scale bar, 10 µm. **j** Quantification of signal accumulation is illustrated; for example, membrane (M) intensity was 5 a.u., cytoplasmic (C) intensity was 1 a.u., resulting in an M/C ratio of 5. Quantification of membrane levels of VANGL1 (in panel **k**) and pVANGL1/2 vs. VANGL1 (in panel **l**). Average membrane fluorescence intensity was normalized to cytoplasmic signal. Statistical analysis was performed using SuperPlots of Data. *n* = 3 biological replicates, ~150 cells per condition; each replicate is shown in a distinct shade of gray, with the corresponding average highlighted in a matching shade of pink and connected by a dashed line. Corresponding *p*-values are reported.

We first examined our proximity-biotinylation data and observed that PRICKLE3 interacts with both CK1δ and CK1ε isoforms (Fig. 5a). We then confirmed that PRICKLE3 interacts with endogenous CK1ε using co-IP in inducible cells (Fig. 5b). To dissect this further, we generated PRICKLE3 deletion constructs corresponding to the N-terminal half (amino acids 1–375; containing the PET and LIM1–3 domains) and the C-terminal half (amino acids 372–615; Fig. 5c). Both fragments were able to bind CK1ε, but the N-terminal region exhibited markedly stronger binding (Fig. 5d). This suggests that the PET-LIM region may act as a scaffold for CK1ε at the plasma membrane or function as a docking platform that influences substrate specificity. Given this interaction, we asked whether CK1ε phosphorylates PRICKLE3, a poorly explored aspect of their relationship, as CK1ε is traditionally viewed as a modulator of other PCP core components but not PRICKLE. Notably, recombinant and commercially available full-length PRICKLE3 protein was phosphorylated by CK1ε in vitro, as shown by a mobility shift in kinase assays (Fig. 5e).

We next tested whether PRICKLE3 could interfere with VANGL2 phosphorylation in a recombinant in vitro system. Specifically, we mixed CK1ε and VANGL2 N-terminus (amino acids 1–100), observing the expected mobility shift upon phosphorylation (figure. S5a). However, excess of recombinant PRICKLE3 C-terminal fragment (amino acids 526–615) reduced VANGL2 phosphorylation in a time-dependent manner (figure. S5a). This suggests that PRICKLE3 can directly compete with VANGL2 for access to CK1ε, thereby limiting its phosphorylation.

To validate these findings in cells, we treated our inducible PRICKLE3-expressing system with the selective CK1δ/ε inhibitor PF-670462 and followed VANGL phosphorylation over time (Fig. 5f–h, Fig. S5b). Strikingly, PRICKLE3 did not further stabilize VANGL1 in the absence of CK1δ/ε activity, indicating that CK1-dependent phosphorylation is a key determinant of VANGL degradation. In the presence of PRICKLE3, pVANGL1/2 vs. VANGL1 levels remained low over time (Fig. 5h), unlike in conditions lacking PRICKLE3, where VANGL is freely phosphorylated by CK1ε (Fig. 5h), consistent with PRICKLE3 limiting CK1-dependent VANGL phosphorylation.

Of note, in the absence of PRICKLE3, we observed a modest increase in the phosphorylated VANGL1/2 pool despite CK1δ/ε inhibition (Fig. 5f), suggesting partial compensation by alternative kinases or incomplete long-term inhibition of CK1δ/ε by PF-670462. To test this, we analysed VANGL phosphorylation in cells overexpressing another candidate member of the CK1 family, CK1α^42,66^. In our setting, CK1α was able to phosphorylate the VANGL N-terminal portion, although to a much lesser extent than CK1ε (Fig. S5c). This aligns with prior PCP studies on CK1α’s role in Vang phosphorylation in *Drosophila*^42,66^ and other studies indicating that CK1 isoforms exhibit overlapping substrate specificity and can compensate for one another in (not only WNT) signaling pathways under conditions of isoform-specific inhibition^67–69^. Collectively, these results indicate that PRICKLE3 limits VANGL phosphorylation and degradation, primarily through modulation of CK1ε activity, but possibly also by affecting other CK1 family members under inhibitor conditions.

#### VANGL membrane accumulation

We then asked whether PRICKLE3 promotes the VANGL accumulation at the plasma membrane. In our inducible system, PRICKLE3 expression significantly increased VANGL1membrane accumulation, as shown by immunofluorescence (Fig. 5i–l, Fig. S5d-e). To investigate whether this recruitment correlates with changes in phosphorylation, we quantified the membrane-associated signal of total VANGL and pVANGL, normalized to cytoplasmic signal to control for transfection variability (Fig. 5j–l, Fig. S5f). These analyses revealed that PRICKLE3 promotes accumulation of VANGL at the membrane while at the same time decreases the pVANGL1/2 vs. VANGL1 ratio (Fig. 5k–l), suggesting that membrane-bound VANGL is preferentially unphosphorylated in the presence of PRICKLE3.

Taken together, our results suggest that PRICKLE3 limits CK1ε-mediated phosphorylation of VANGL, likely through competitive binding to CK1ε. This uncovers a previously unrecognized mechanism whereby PRICKLE3 modulates the phosphorylation status and membrane accumulation of VANGL, providing a new regulatory layer in the WNT/PCP signaling cascade in vertebrates.

### PRICKLE3 protects VANGL from RNF43-mediated ubiquitination

We next investigated the molecular basis of VANGL stabilization by PRICKLE3, focusing on how it protects VANGL from degradation. In eukaryotic cells, two major pathways mediate protein degradation: the ubiquitin-proteasome system and the autophagy-lysosome pathway^70^. Given this, we concentrated on E3 ubiquitin ligase RNF43 and its close homolog ZNRF3, which are transmembrane enzymes removing WNT receptors FZDs and LRP5/6 from the cell surface^71^. RNF43/ZNFR3 also interact with and inhibit key components of the non-canonical WNT5A pathway, including ROR1/2, VANGL1/2, DVL1-3, and CK1 kinases^51,72–75^.

**Fig. 6:**
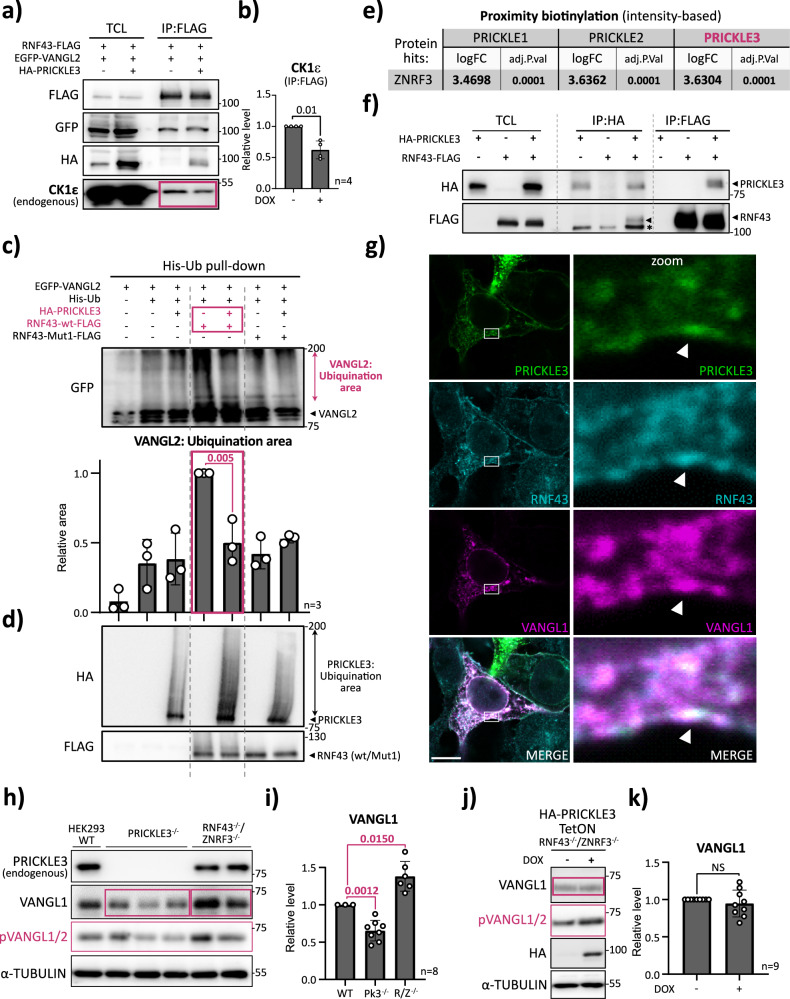
PRICKLE3 protects VANGL from RNF43-mediated ubiquitination. **a** Co-immunoprecipitation of RNF43–VANGL2 complexes in the presence or absence of PRICKLE3. HEK T-REx 293 PRICKLE3 TetON cells were transfected with RNF43-FLAG and EGFP-VANGL2 constructs. PRICKLE3 expression was induced by overnight doxycycline treatment. Representative experiment from *n* = 4. **b** Densitometric quantification of endogenous CK1ε Western blot signal. Intensities were normalized to levels in doxycycline-untreated cells. Statistical analysis was performed using an unpaired *t*-test; corresponding *p*-values are reported (*n* = 4). **c**, **d** Ubiquitination assay. In **c**, HEK T-REx 293 PRICKLE3 TetON cells were transfected with His-tagged ubiquitin, EGFP-VANGL2, and FLAG-tagged RNF43 constructs (either wild-type or enzymatically inactive Mut1 variant). PRICKLE3 expression was induced by overnight doxycycline treatment. Ubiquitinated proteins were enriched by His pull-down and analysed by Western blot. The area corresponding to ubiquitinated VANGL2 is indicated. Representative experiment from *n* = 3; additional replicates shown in Supplementary Fig. 6A. In panel **d**, Input control membranes probed for HA-tagged PRICKLE3 and FLAG-tagged RNF43 expression. **e** Table summarizing proximity biotinylation results for ZNRF3 protein, showing log₂ fold change (log₂FC) and –log₁₀(adjusted p-value). Values with log₂FC > 1.00 and *p* < 0.05 are highlighted in bold. **f** Interaction between RNF43 and PRICKLE3. HA-tagged PRICKLE3 and FLAG-tagged RNF43 were co-expressed in HEK293 wild-type cells. Co-immunoprecipitation was performed using either anti-HA or anti-FLAG antibodies and analysed by Western blot. Representative experiment from *n* = 3. TCL Total Cell Lysate. Asterisk (*) denotes non-specific IgG signal arising from the IP procedure. **g** Immunofluorescence imaging showing subcellular co-localization of PRICKLE3, RNF43, and VANGL1. HEK293 wild-type cells were transfected with HA-PRICKLE3, MYC-VANGL1, and RNF43-FLAG constructs. Proteins were visualized using tag-specific antibodies. Arrowheads indicate sites of co-localization. Scale bar, 10 µm. **h** Western blot analysis of VANGL1 protein levels in CRISPR-Cas9-modified HEK293 cell lines. Loss of *PRICKLE3* resulted in reduced VANGL1 levels, while *RNF43/ZNRF3* double knockout led to VANGL1 accumulation. **i** Densitometric quantification of VANGL1 signal from panel h. Intensities were normalized to the loading control and compared to wild-type cells. Statistical analysis was performed using an unpaired *t*-test; *n* = 2–3 independent clones per condition. **j** Western blot analysis showing that doxycycline-induced PRICKLE3 expression in RNF43/ZNRF3-deficient cells does not further increase VANGL1 protein levels, supporting the hypothesis that PRICKLE3 acts via RNF43. **k** Densitometric quantification of VANGL1 signal from **j**. Induced and uninduced samples were compared; no significant difference was observed. Statistical analysis was performed using an unpaired *t*-test; *n* = 3 clones.

Given that RNF43 robustly ubiquitinates VANGL2, leading to its proteasomal degradation^51^, we wanted to determine whether PRICKLE3 interferes indirectly with this process. We first performed co-IP experiments, which revealed a physical interaction between PRICKLE3 and RNF43 (Fig. 6a; see also Fig. 6f, showing PRICKLE3 pulldown by RNF43 in the absence of VANGL2). Given the fact that RNF43 activity is regulated by phosphorylation via CK1 family members^72,73^, we next asked whether CK1ε is present in this complex, and whether PRICKLE3 affects its association. We quantified the CK1ε levels within the VANGL2–RNF43 complexes and observed that PRICKLE3 overexpression significantly reduced CK1ε recruitment to RNF43 (Fig. 6b). This suggests that PRICKLE3 may inhibit RNF43 activity by disrupting its interaction with CK1ε. Notably, total VANGL2 levels remained unchanged in these assays, implying that PRICKLE3 shields CK1ε from accessing the RNF43–VANGL2 complex, either through direct CK1ε sequestration or by altering the complex architecture. These findings suggest that PRICKLE3 reduces RNF43 activity by limiting CK1ε availability within the complex, thereby indirectly stabilizing VANGL2.

To investigate whether PRICKLE3 directly affects the ubiquitination of VANGL, we used an established His-tagged ubiquitin pulldown assay^51^ to assess RNF43-mediated ubiquitination of VANGL2 in the presence or absence of PRICKLE3. VANGL2 was co-expressed with either wild-type RNF43 or an enzymatically inactive variant (Mut1)^51,75^, which served as a robust negative control. We found that VANGL2 ubiquitination by wild-type RNF43 was markedly reduced when PRICKLE3 was present, while no such reduction occurred with the inactive RNF43 variant (Fig. 6c, Fig. S6a). These results indicate that PRICKLE3 can directly inhibit RNF43’s enzymatic activity.

To our surprise, PRICKLE3 itself appeared ubiquitinated (see HA-PRICKLE3 immunoblot in Fig. 6d), yet was not subject to degradation, prompting us to examine whether PRICKLE3 physically interacts with RNF43. Proximity biotinylation and co-immunoprecipitation experiments confirmed this interaction (Fig. 6e–f). Furthermore, confocal microscopy revealed that PRICKLE3, VANGL2, and RNF43 co-localize within the same subcellular compartments near the plasma membrane (Fig. 6g). This spatial proximity suggests that PRICKLE3 may act locally to interfere with RNF43-mediated VANGL2 ubiquitination by modulating their interactions at the site of action.

#### Loss-of-function validation using CRISPR-Cas9

As an ultimate test of our model, we used CRISPR-Cas9 to generate targeted loss-of-function HEK293 cell lines. Whereas most of our study relied on PRICKLE3 induced overexpression, we now generated *PRICKLE3*-deficient cells, and additionally made use of pre-existing *RNF43/ZNRF3* double KO cell lines described previously^76^. To further explore functional rescue, we cloned a DOX-inducible PRICKLE3 transgene into these *RNF43/ZNRF3* mutant cells. For all analyses, we used three independent *PRICKLE3*-deficient clones (Fig. S6b), two *RNF43/ZNRF3*-deficient clones^76^, and three inducible PRICKLE3 clones in the *RNF43/ZNRF3*-deficient background (Fig. S6d).

As predicted, loss of endogenous PRICKLE3 led to reduced VANGL1 levels, consistent with its stabilizing function (Fig. 6h, i). Conversely, loss of endogenous RNF43 resulted in increased VANGL1 levels, consistent with its role in promoting degradation (Fig. 6h, i). In agreement with this, pVANGL1/2 vs. VANGL1 levels were elevated in PRICKLE3-deficient cells (Fig. S6c), indicating that the absence of PRICKLE3 removes its protective effect against CK1ε-mediated phosphorylation. In contrast, RNF43/ZNRF3 deficiency had no significant impact on pVANGL1/2 vs. VANGL1 levels (Fig. S6c), suggesting that in the absence of the E3 ligases, phosphorylated VANGL simply accumulates due to impaired degradation of its phosphorylated form.

Notably, we also observed that endogenous PRICKLE3 levels were reduced in RNF43/ZNRF3-deficient cells (Fig. S6d), suggesting that RNF43-mediated PRICKLE3 ubiquitination may paradoxically stabilize it. This aligns with our ubiquitination assay (Fig. 6d) and supports a model in which RNF43 interacts with and ubiquitinates both PRICKLE3 and VANGL, but with opposing consequences: PRICKLE3 stabilization versus VANGL degradation. In this context, PRICKLE3 may act as a molecular shield, diverting RNF43 activity away from VANGL and thereby protecting it from ubiquitin-mediated degradation.

Finally, in the *RNF43/ZNRF3*-deficient cells carrying inducible PRICKLE3 (Fig. S6e), we reasoned that PRICKLE3 would have no further effect, as VANGL could no longer be targeted for degradation via RNF43. Indeed, PRICKLE3 induction in this background failed to increase VANGL levels, confirming that RNF43 is the critical PRICKLE3 target in this context (Fig. 6j, k, Fig. S6f). Thus, genetic evidence confirms that RNF43/ZNFR3 is the primary target through which PRICKLE3 exerts its stabilizing effect on VANGL.

Using both gain-of-function and loss-of-function approaches, we show that PRICKLE3 effectively disrupts RNF43/ZNRF3-mediated VANGL1/2 degradation, likely by directly interfering with RNF43 – either by acting as its substrate or by excluding CK1ε from the RNF43 complex, thereby preventing its activation.

### RNF43 downregulates VANGL complexes from membranes in vivo

**Fig. 7:**
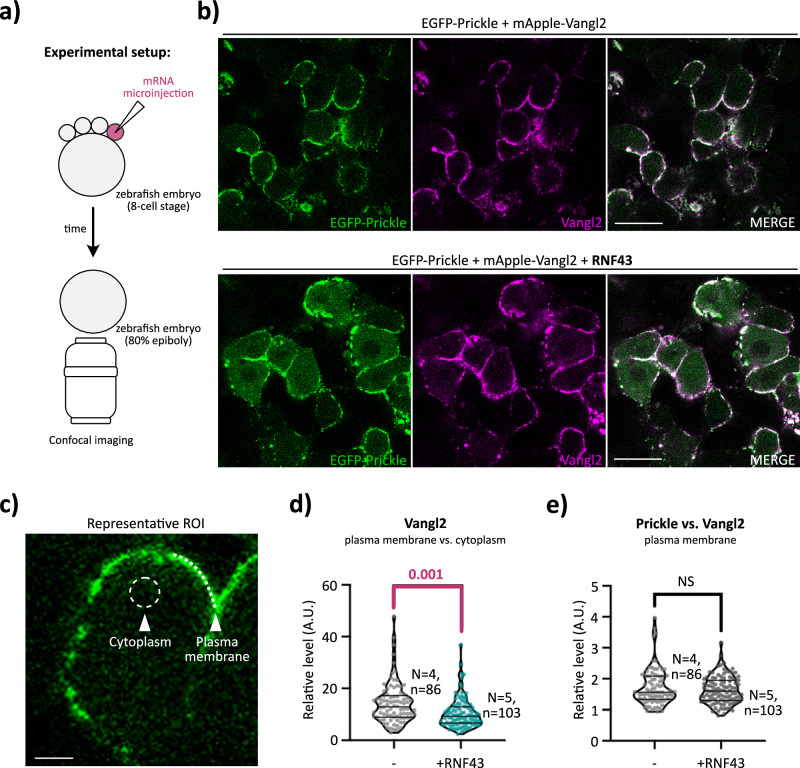
RNF43 downregulates VANGL complexes from membranes in vivo. **a** Schematic of the experimental setup. Wild-type zebrafish embryos were co-injected at the 8-cell stage into a single dorsal blastomere with 100 pg of *Drosophila EGFP-Prickle* mRNA and 40 pg of zebrafish *mApple-Vangl2* mRNA (approximately a 1.5:1 molar ratio), or with 100 pg of *EGFP-Prickle*, 40 pg of *mApple-Vangl2*, and 40 pg of human *RNF43* mRNA. Embryos were dorsally mounted and imaged by confocal microscopy at 80% epiboly. Imaging was performed at comparable Z-depths within the enveloping epithelium. **b** Representative single-plane confocal images showing cells overexpressing EGFP-Prickle and mApple-Vangl2, with or without co-expression of human RNF43. Scale bar, 20 μm. **c** Representative region of interest (ROI) used for quantitative image analysis, as shown in panel d. Mean fluorescence intensities of EGFP-Prickle and mApple-Vangl2 at the plasma membrane were measured using the line tool in ImageJ (dashed line). Cytoplasmic intensities were measured in adjacent regions using the circle tool (dashed circle). Scale bar, 5 μm. **d**, **e** Relative membrane localization of EGFP-Prickle and mApple-Vangl2 was calculated by dividing membrane intensity by cytoplasmic intensity for each marker. The ratio of membrane-localized EGFP-Prickle to membrane-localized mApple-Vangl2 was also calculated (in panel **e**). Statistical analysis was performed using the Mann–Whitney test with a 95% confidence interval. Exact *p*-values are indicated for each comparison. The number of embryos and individual cells analysed are denoted as *N* and *n*, respectively.

To further assess whether RNF43 mediate VANGL internalization in a physiologically relevant setting (Fig. 7a), we used zebrafish embryos, where the PCP pathway is strongly active during convergence–extension movements at 80% epiboly^77,78^. Specifically, we employed zebrafish embryos and co-expressed RNF43 together with VANGL and PRICKLE orthologs – Vangl2 from zebrafish^79^ and Prickle derived from *Drosophila*^80^. When co-expressed with EGFP-Prickle, mApple-Vangl2 localized robustly to the plasma membrane of superficial epithelial cells at 80% epiboly, as visualized by confocal microscopy (Fig. 7b). This pattern was consistent across multiple embryos and Z-depths, and quantitative region of interest (ROI) analysis confirmed strong membrane enrichment (Fig. 7c).

However, upon the addition of human RNF43, the membrane-associated levels of Vangl2 were significantly reduced, which was accompanied by increases in cytoplasmic distribution (Fig. 7d). This result aligns with the known function of RNF43 as a transmembrane E3 ubiquitin ligase that internalizes WNT pathway components such as Vangl^51^. Intriguingly though, the Prickle-to-Vangl2 ratio at the plasma membrane remained constant (Fig. 7e), suggesting that in zebrafish embryos, Prickle and Vangl2 form a stable complex that is either co-internalized or co-regulated.

These data indicate that Prickle preserves membrane Vangl2 even under elevated RNF43 activity, likely by shielding it from recognition or internalization. This in vivo evidence supports a model in which Prickle stabilizes Vangl2 by preventing degradation and modulating its membrane dynamics.

## Discussion

This study is the first to utilize the miniTurboID assay for a direct comparison of human PRICKLE1-3 interactomes. An important advantage of this method over standard IP is that cell lysis can be performed in strong detergents, enabling isolation of hydrophobic proteins, while the extraordinarily strong non-covalent biotin–streptavidin interaction ensures efficient recovery of reaction products. This makes miniTurboID particularly powerful to map transient or hydrophobic interactors^31^.

Through this approach, we confirmed previously known PRICKLE protein interactors and uncovered potential novel interactions. For instance, Misshapen-like kinase 1 and Casein kinase (CK1 and CK2) kinases, which were previously described as PRICKLE1 interactors^26,81,82^, were also identified for PRICKLE2-3 in our dataset. Notably, all our baits significantly interact with CK1δ/ε, which was not previously reported for all three PRICKLE paralogs. Based on our datasets, PRICKLE3 can interact with SRCIN1 (also known as SNAP-25-Interacting Protein) which was shown to destabilize β-catenin and affect the composition of the immune tumor microenvironment in breast cancer^83^ and potentially be involved in the Src and STAT3 signaling in neuroblastoma^84^. The *Xenopus laevis* homolog of another protein found here – epbl41l4a – is expressed in the rotating somites and the forming neural tube, suggesting potential involvement of PRICKLE3 in the WNT/PCP pathway-driven development^85^. Another example is CEP290, one of the strongest PRICKLE1 interactors, which is a master regulator of the ciliary transition zone and a key component in nearly all known ciliopathies^86^. These examples highlight the significance of our work and indicate potential future directions (see Fig. 1f for more examples).

As for subcellular specificity and functional PRICKLE paralog divergence, our findings from proximity analyses indicate that PRICKLE1-3 interactors are quite uniformly localized within cells— primarily at the periphery, centrosomes, endomembrane system, and mitochondrial matrix. Of note, to date, only PRICKLE3 has been linked to mitochondria, as it has been associated with mitochondrial dysfunction and ATPase biogenesis^87,88^. Additional investigation is needed to understand how and whether PRICKLE paralogs signal to mitochondria.

When we focused on the paralog-specific interactions in context of signaling pathways, already at an early stage of working with proximity data PRICKLE3 appeared different from PRICKLE1/2. Subsequent analysis revealed that the proximal PRICKLE3 microenvironment is enriched in the proteins forming the GO (Gene ontology) term dedicated to the non-canonical WNT/PCP pathway (PID: M23). Indeed PRICKLE3, fused with miniTurboID enzyme, marked the most significant (membrane-bound) components in non-canonical WNT signaling, including VANGL1/2, CELSR1/2, and ROR1/2 (refs. ^20,28,89^). This directly suggested involvement in the signaling events occurring at the plasma membrane, which was subsequently experimentally validated (for instance, see Figs. 5i or 7b). In line with this, PRICKLE3, but not PRICKLE1, could stabilize membrane-localized VANGL and enhance ROR2 trafficking in a WNT5A-dependent manner (e.g., Fig. 4f).

The vertebrate VANGL proteins, named after *Drosophila* Van Gogh (Vang), also known as Strabismus (Stbm)^90^, are core components in the WNT/PCP pathway. In contrast to *Drosophila*, which encodes a single gene, vertebrates possess two paralogs, which share over 70% sequence identity^38^. Although VANGL1 and VANGL2 exhibit distinct spatiotemporal expression patterns and mutations in either gene resulting in variable phenotypes, functional studies indicate substantial redundancy between the two paralogs, suggesting that they act in concert to ensure robust PCP signaling in both vertebrate development and disease^91,92^.

To explore how VANGL function is shaped by its direct binding partners, we focused on PRICKLE proteins, the best-characterized VANGL interactors to date^15,37–39,57,65,79,93–101^. Our binding assays (Fig. S2a-b) together with bioinformatic analyses demonstrated that all three PRICKLE paralogs (PRICKLE1–3) associate with both VANGL1 and VANGL2, likely via shared interaction interfaces, as suggested recently^38^. Based on these observations, VANGL1 and VANGL2 were treated interchangeably in our PRICKLE-mediated experimental setup.

Among the three PRICKLE paralogs, PRICKLE3 consistently exhibited the strongest association with both VANGL paralogs (see Fig. 1f; Fig. 2b–d; Figs. S1–2). This result was unexpected, given that PRICKLE3 has been widely reported to lack a VANGL-binding domain (VBD), e.g., refs. ^15,37^. However, our data provide clear evidence that PRICKLE3 not only contains VANGL-binding motifs (VBMs) that are functional and sufficient to interact with VANGL (see Fig. 2). This observation aligns with prior studies from the Sokol group, which identified PRICKLE3–VANGL2 complexes in *Xenopus* embryos, particularly during neural plate morphogenesis^65,96,97,102–104^. While PRICKLE1 and PRICKLE2 also bind to VANGL and certainly participate in PCP signaling, only PRICKLE3 appears to exert a stabilizing effect on VANGL-containing complexes, reminiscent of the ancestral function of *Drosophila* Prickle in core PCP complex maintenance^43^. Together, these findings suggest that PRICKLE3 may play a more direct and possibly underappreciated role in the assembly or stabilization of vertebrate PCP signaling platforms.

Regarding mechanisms of VANGL degradation, previous findings demonstrated that VANGL proteins are predominantly degraded via the proteasomal pathway^40^ and RNF43/ZNRF3 are involved in this process^51^. Collectively, our data reveal that PRICKLE3 stabilizes VANGL through multiple complementary mechanisms: (i) inhibition of CK1ε-mediated phosphorylation of the VANGL N-terminus, which may act as a phospho-degron and mark VANGL for proteasomal degradation; (ii) by acting as a decoy substrate for CK1ε, thus buffering its kinase activity away from other critical targets such as VANGL or RNF43; and (iii) interference with the E3 ubiquitin ligase RNF43.

As for the first point, the role of CK1 kinases in protein degradation is well established. For example, CK1α promotes the proteasomal degradation of β-catenin, and CK1δ targets MDM2 (refs. ^105–107^). In line with this paradigm, our data suggest that CK1ε can similarly target membrane-bound VANGL for degradation (Figs. 4, 5). Specifically, we observed that VANGL proteins are more stable at the plasma membrane when CK1ε activity—and consequently VANGL phosphorylation—is moderate. Consistently, in *RNF43/ZNRF3*-deficient cells, we detected increased total VANGL protein levels. In this double-knockout background, PRICKLE3 induction did not further increase VANGL abundance, indicating that PRICKLE3 acts through RNF43/ZNRF3 to control VANGL stability. These results provide strong evidence that CK1ε-mediated phosphorylation of the VANGL N-terminus functions as a phospho-degron regulating its membrane localization and proteasomal degradation. We further show that PRICKLE3 is capable of reducing this phosphorylation, further supporting its role in VANGL stabilization and extending current models of VANGL trafficking and turnover.

Notably, our findings do not contradict prior studies showing that non-phosphorylatable VANGL mutants are unstable^40,41^. Rather, we propose that PRICKLE3 binds VANGL specifically at the plasma membrane and protects it from excessive phosphorylation-dependent removal. This concept is supported by studies in *Drosophila melanogaster*, where excessive phosphorylation of Vang’s N-terminus reduces its membrane retention, and Prickle acts as a protective factor^43,108^. For instance, the phospho-mimetic S82E/S84E Vang mutant is depleted from the membrane, while the non-phosphorylatable S82A/S84A variant remains membrane-associated. Similarly, a dominant-negative CK1ε mutant (K38R) leads to increased VANGL membrane stability, when compared to wild-type CK1ε. Comparable results were reported in vertebrates: in *Xenopus*, phosphorylated VANGL accumulates on the posterior side of the neural plate (induced by Frizzled3) and is internalized, while unphosphorylated anterior VANGL preferentially binds PRICKLE3 and remains membrane-bound^97^. In the mouse limb, CK1ε-dependent phosphorylation correlates with lower VANGL2 levels proximally, where VANGL2 is more phosphorylated, compared to higher, less phosphorylated levels distally^41^.

However, Kelly et al.^42^ reported that CK1ε promotes VANGL stabilization at the membrane. While this interpretation may reflect model-specific dynamics, Strutt et al.^43^ observed the opposite phenotype using the same *Drosophila* system, highlighting the complexity of CK1ε-dependent VANGL regulation. Therefore, further studies are needed to dissect the precise role of CK1ε in invertebrate PCP signaling. Our findings, in combination with vertebrate studies, suggest that CK1ε-dependent phosphorylation may facilitate VANGL membrane removal, a process that could be modulated by PRICKLE3.

Second, we provide direct evidence that PRICKLE3 is a CK1ε substrate. In vitro kinase assays using recombinant proteins confirmed that CK1ε phosphorylates PRICKLE3, identifying a novel post-translational modification of this paralog. While our previous work reported interaction between PRICKLE2 and CK1ε^24^, the findings shown in this study represent the first demonstration that PRICKLE3 is directly phosphorylated by CK1ε. These results suggest that PRICKLE3 could act as a decoy substrate for CK1ε, thereby diverting kinase activity from other targets such as VANGL2 or RNF43. This mechanistic view is consistent with earlier observations in *Drosophila*^43^, where Prickle was proposed to protect Vang from phosphorylation, although the underlying mechanism was not defined. Our study provides the first experimental evidence supporting a substrate-competition model for this type of regulation.

Third, we propose that PRICKLE3 interferes with RNF43 function. Rather than directly competing with RNF43 for CK1ε access, PRICKLE3 may act as a decoy substrate for CK1ε, thereby reducing VANGL phosphorylation and subsequent RNF43-mediated ubiquitination. This model aligns with prior reports showing that PRICKLE2, in complex with VANGL2, is subject to polyubiquitination^57^. In our system, we observed PRICKLE3 ubiquitination in the presence of VANGL2 and RNF43, implying functional competition at the enzymatic activation or substrate occupancy levels. Surprisingly, our data show that RNF43 interacts with and ubiquitinates both PRICKLE3 and VANGL, but with opposing consequences: PRICKLE3 stabilization versus VANGL degradation. In this context, PRICKLE3 may act as a molecular shield, diverting RNF43 activity away from VANGL and thereby protecting it from ubiquitin-mediated degradation. These data support a model in which PRICKLE3 limits RNF43-mediated VANGL ubiquitination either through CK1ε sequestration or by acting as a competitive substrate^72^.

**Fig. 8:**
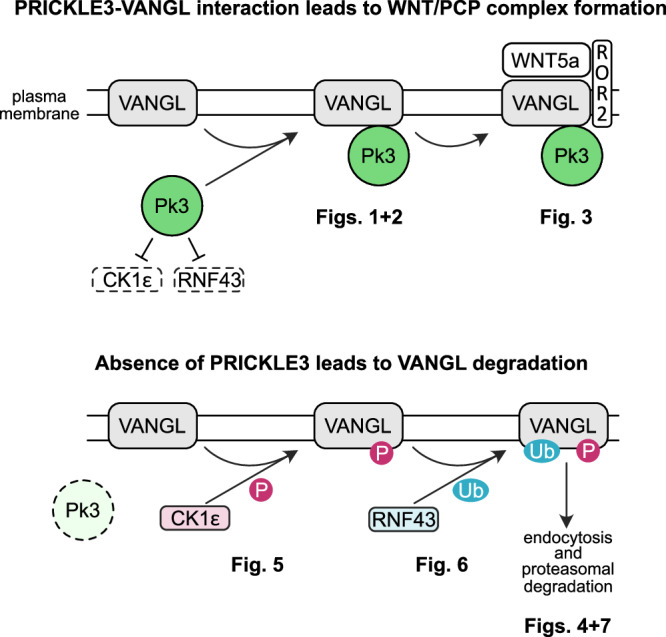
Summary of PRICKLE3 function, emphasizing its role and mechanisms of action in the stabilization of VANGL proteins. Proposed model of PRICKLE3 function in regulating VANGL stability and WNT/PCP signaling. Top Protection from RNF43-mediated ubiquitination. PRICKLE3 disrupts this CK1ε/RNF43 feedback loop. It results in the stabilization of VANGL1/2 and ROR2 protein activation at the plasma membrane. Also, phosphorylation of membrane-bound VANGL1/2 is reduced. Bottom Negative regulation of WNT/PCP complexes by RNF43. Casein kinase 1ε plays a dual role in the regulation of WNT/PCP complexes. CK1ε phosphorylation is essential for the proper translocation of VANGL proteins to the plasma membrane. The activity of this kinase is also required for the function of the FZD and ROR, the WNT receptors and co-receptors. In addition, the enzymatic activity of the E3 ubiquitin ligase RNF43, a negative regulator of WNT signaling, is also promoted by CK1ε. As a result, WNT/PCP plasma membrane complexes are degraded. This relationship establishes a CK1ε/RNF43-mediated negative feedback loop that tightly controls the activation level of the PCP signaling pathway.

These three PRICKLE3-dependent mechanisms likely complement each other in regulating VANGL stability; however, the degree to which each contributes under physiological conditions remains beyond the scope of this study. Taken together, our findings expand the current understanding of how PRICKLE3 may counteract RNF43-mediated disassembly of WNT5A/PCP receptor complexes, which are organized around VANGL as a core structural component^51^. A consolidated overview of PRICKLE3’s molecular functions is presented in Fig. 8, illustrating the distinct mechanisms by which PRICKLE3 interacts with CK1ε and RNF43 to regulate VANGL stability and modulate positively non-canonical WNT signaling at the plasma membrane. Nevertheless, without an assay to directly measure this “activation,” the functional state of these complexes remains uncertain, and future studies will be required to address this important question.

In the context of evolutionarily conserved regulation of Vang/VANGL ubiquitination, our findings, along with previous research^51^, support the model in which RNF43 ubiquitinates VANGL. However, the classic PCP model organism *Drosophila melanogaster* lacks a homolog of RNF43 (ref. ^109^), implying that alternative E3 ubiquitin ligases must fulfill this role in this invertebrate model. In flies, phosphorylated Prickle is targeted for proteasomal degradation by a distinct E3 ligase complex composed of Cullin1 (Cul1), SkpA, and Supernumerary limbs (Slimb), following phosphorylation by the Nemo kinase^56,110^. These differences suggest that the ubiquitin-mediated regulation of PCP components has diverged significantly between *Drosophila* and vertebrates. In vertebrates, RNF43 also targets other core WNT components such as Frizzled, reinforcing its specialized and possibly vertebrate-specific role in coordinating the broader WNT/PCP signaling complexity. Rather than representing a gap in evolutionary conservation, the absence of RNF43 in *Drosophila* may instead highlight the emergence of additional regulatory layers required for the spatial and temporal refinement of PCP signaling in vertebrate tissues.

When examining regulation of Dishevelled by PRICKLE proteins, we observed binding between Dishevelled and PRICKLE proteins, with PRICKLE3 showing a preferential interaction with a post-translationally modified form of DVL that appears distinct from the forms bound by PRICKLE1 and PRICKLE2 (Fig. S2a-b, and Fig. S2c for proximity biotinylation data). The interaction between PRICKLE and DVL is consistent with previous reports^58,94,101,111,112^. However, in contrast to earlier studies, we did not observe a reduction in DVL protein levels or phosphorylation states upon PRICKLE induced overexpression^54,55,101,113^. Specifically, neither DVL2 nor DVL3 levels appeared altered in our experimental system.

This discrepancy may be explained by several factors, including differences in experimental systems, such as cell type or species. For example, Tree et al.^101^ reported PRICKLE-mediated Dsh/DVL degradation in *Drosophila*, and similar effects were seen in human hepatocellular carcinoma cells^55^ and in neural crest-derived neuroblastoma C1300 cells^113^. On the other hand, PRICKLE expression has been associated with increased DVL levels in acute myeloid leukemia (AML) samples^114^. Moreover, technical aspects such as antibody specificity may contribute to variable results; for instance, the anti-DVL3 antibody (sc-8027, Santa Cruz Biotechnology) has been shown to produce different banding patterns depending on the DVL3 phosphorylation status^115^. Finally, our use of a doxycycline-inducible (TetON) system to control PRICKLE3 expression may lead to different dynamics than the constitutive overexpression used in other studies.

Taken together, these findings suggest that PRICKLE’s influence on DVL levels is highly context-dependent, with effects ranging from degradation to stabilization or no change at all. Thus, further investigation is required to define the determinants underlying this variability.

On the topic of implications for WNT signaling and disease, the dataset generated in this study has been rigorously validated and provides detailed insights into the cellular microenvironment of PRICKLE proteins. We believe this dataset will be a valuable resource for advancing the understanding of PRICKLE protein functions in human cells, particularly in the context of cellular signaling and structural dynamics, including pathological contexts.

We have established a functional connection between PRICKLE3 and RNF43, an E3 ubiquitin ligase with key roles in development and tumorigenesis^116,117^. RNF43 has been widely implicated in cancers of the gastrointestinal tract, skin, ovaries, and other tissues due to its role in modulating WNT signaling, a pathway essential for both normal cellular growth and oncogenesis. RNF43 dysregulation disrupts this pathway, contributing to tumor progression and metastasis^117,118^. Our findings suggest that PRICKLE3 may modulate RNF43 activity, revealing a novel regulatory axis in the non-canonical WNT pathway with potential disease relevance.

## Materials and methods

### Cell culture

All cell lines utilized in this study (Table S1) were cultured in Dulbecco’s Modified Eagle Medium (DMEM, 41966-029, Gibco, Life Technologies), supplemented with 10% fetal bovine serum (FBS, 10270-106, Gibco, Life Technologies) and 1% penicillin-streptomycin (XC-A4122/100, Biosera). The cells were maintained at 37 °C in a 5% CO_2_ humidified atmosphere. Routine tests for mycoplasma contamination were regularly conducted to ensure the absence of contamination in the cell cultures.

### Cells treatments

Cells with a doxycycline-inducible system were treated with 0.1–1 μg/ml of doxycycline (HY-N0565B, MedChem Express) overnight to induce protein expression.

To inhibit endogenous WNT ligands, cells were treated with 1 μM LGK-974 (1241454, PeproTech) overnight. Following this, non-canonical WNT signaling was activated by incubating the cells with recombinant human WNT5A (645-WN, R&D Systems) at a concentration of 100 ng/mL for 3 h. Similarly, canonical WNT signaling was activated by incubating the cells with 100 ng/mL of recombinant human WNT3A (5036-WN, R&D Systems) for 3 h.

To assess protein degradation kinetics, cells were pretreated with doxycycline overnight. The following day, the cells were treated with 20 μM cycloheximide (Sigma-Aldrich, C7698) and harvested at various time points for analysis.

To inhibit CK1ε activity, cells were treated with 2.5 μM PF670462 (sc-204180, Santa Cruz), for the indicated time.

### Preparation of inducible stable cell lines

HEK293 T-REx PRICKLE3 and PRICKLE1 TetON cell lines were generated by transfecting HEK293 T-REx cells (R71007, Invitrogen) with the pcDNA4TO-zeo plasmid encoding N-terminally HA-tagged PRICKLE genes. Similarly, HEK293 T-REx miniTurboID TetON cell lines were established by transfecting HEK293 T-REx cells with pcDNA4/TO-zeo plasmids encoding human PRICKLE1-3 proteins, tagged at the N-terminus with miniTurboID biotin ligase and a V5 tag, or with a control plasmid containing only the miniTurboID enzyme and a V5 tag (details in Table S2). Inducible PRICKLE3 TetON cell lines derived from MDCK cells and RNF43/ZNRF3 knockout cells^76^ were generated using the same transfection and selection strategy as for HEK293 wild-type cells. Twenty-four hours post-transfection, cells were selected using 100 µg/mL of Zeocin (ant-zn-1p, Invivogen). Subsequently, single clones were isolated, propagated, and validated through Western blot analysis and immunofluorescence.

### Preparation of CRISPR/Cas9 cells

To target the *PRICKLE3* gene in HEK293 T-REx cells, a previously established protocol was followed^76^. In brief, the guide RNA (gRNA) sequence *TCCATCTCCGACGACGACTC* (*AGG –* PAM seq.) was cloned into the gRNA_GFP-T1 vector backbone. The resulting construct was transfected into cells in combination with PiggyBac-Hygro and transposase-expressing plasmids using the JetOPTIMUS transfection reagent. Following transfection, cells were subjected to selection with hygromycin B and subsequently plated as single cells into 96-well plates. To identify clones harboring CRISPR/Cas9-induced modifications in both alleles of the target gene, restriction fragment length polymorphism (RFLP) analysis was performed using HinfI (ER0801, Thermo Fisher Scientific) restriction enzyme. Subsequently, all clones were sequenced using the Illumina platform and compared with the reference sequence^119^. Changes in the targeted sequences are shown in Fig. S6b.

### Plasmids/cloning

The generation of all plasmids involved the amplification of sequences using Q5® High-Fidelity DNA Polymerase (M0491S, New England Biolabs), followed by cloning utilizing the In-Fusion cloning method (639690, Takara Bio). All plasmids utilized in this study, including those generated in this work and those obtained from previous studies, are listed in Table S2.

To generate the TetON-inducible pcDNA4-HA-PRICKLE3 plasmid, the cDNA encoding PRICKLE3 was amplified from the pGFP_LMO6 (PRICKLE3) plasmid. For the construction of the TetON-inducible pcDNA4-HA-PRICKLE1 plasmid, the cDNA encoding PRICKLE1 was amplified from the pCMV6-XL5-Prickle1 plasmid. The amplified sequences were subsequently cloned into the pcDNA4-TO-RNF43-2xHA-2xFLAG plasmid, which had been linearized using HindIII (ER0501, Thermo Fisher Scientific) and XbaI (ER0681, Thermo Fisher Scientific).

For the generation of the pcDNA4-CTRL-miniTurboBioID plasmid, the cDNA encoding V5-miniTurboID was amplified from the pCW57-EPHA2-V5-miniTurboID plasmid. Similarly, to generate the pcDNA4-PRICKLE1-3 miniTurboBioID plasmids, the cDNA encoding V5-miniTurboID was amplified from the pCW57-EPHA2-V5-miniTurboID plasmid, and the PRICKLE1-3 sequences were amplified from the pCMV6-XL5-Prickle1, pGateway 3XFlag Prickle2, and pGFP_LMO6 (PRICKLE3) plasmids, respectively. The resulting sequences were cloned into the pcDNA4-TO-RNF43-2xHA-2xFLAG plasmid which had been linearized using HindIII and XbaI.

The pcDNA3-RNF43-FLAG and pcDNA3-RNF43 Ring mut-FLAG plasmids were generated by PCR skipping the HA tag from the pcDNA4-TO-RNF43-2xHA-2xFLAG and pcDNA4-TO-RNF43Mut1-2xHA-2xFLAG plasmids, respectively. The sequences were then cloned into pcDNA3, which had been linearized using HindIII and EcoRV (ER0301, Thermo Fisher Scientific).

The plasmids pGFP_PRICKLE3 (aa 1–375) and pGFP_ PRICKLE3 (aa 372–615) were generated by site-directed mutagenesis using the XLII QuikChange kit (Agilent, Cat. No. 200521). The N-terminal truncation construct (aa 1–375) was produced using the primers: Pk3 N-term forward 5’-AGCCCACAGCTCCAGGGCCGAGCTGAAGGCAGAATCGAATTCTGCA-3’ and Pk3 N-term reverse 5’-TGCAGAATTCGATTCTGCCTTCAGCTCGGCCCTGGAGCTGTGGGCT-3’. The C-terminal truncation construct (aa 372–615) was generated using the primers: Pk3 C-term forward 5’-TGTACAAGTCCGGACTCAGATCCCCCACAGCTCCAGGGCCGAGCCG-3’ and Pk3 C-term reverse 5’-CGGCTCGGCCCTGGAGCTGTGGGGGATCTGAGTCCGGACTTGTACA-3’.

### Transfection

Cells were transfected using the jetOPTIMUS transfection reagent (117-07, Polypus) at a 1:1 ratio with plasmid DNA, or alternatively, transfections were performed using 1 μg/ml polyethyleneimine (PEI) (23966-100, Polysciences) at pH 7.4, with plasmid DNA in a 4:1 ratio. The culture media were replaced 6 h post-transfection. The amount of plasmid DNA used for transfection was as follows: 300 ng per well of a 24-well plate for antibody verification, 2.5 µg for a 4 cm culture dish for ubiquitination assays, 10 µg for a 10 cm dish for co-immunoprecipitation or stable cell line preparation.

### Western-blot analysis

Cells were lysed directly in a buffer containing 100 mM Tris/HCl (pH 6.8), 20% glycerol, 1% SDS, 0.01% bromophenol blue, and 1% 2-mercaptoethanol. Protein separation by SDS-PAGE was performed according to the manufacturer’s protocol with minor modifications (Bio-Rad). Briefly, proteins were resolved on an SDS-PAGE gel at 110 V and subsequently transferred to an Immobilon-P PVDF membrane (Millipore, IPVH00010) for 1 h at 100 V. Membranes were then blocked in a 5% non-fat dry milk solution prepared in TBS-T for 30 min and incubated overnight at 4 °C with primary antibodies. Following incubation, membranes were washed in TBS-T and treated with HRP-conjugated secondary antibodies. Protein detection and visualization were carried out using the Fusion SL imaging system (Vilber) with the Immobilon Western Chemiluminescent HRP Substrate (Merck, WBKLS0500). A complete list of antibodies used in this study is provided in Table S3.

### Immunoprecipitation

Cells were transfected with the appropriate plasmid DNA and/or induced with doxycycline overnight and cultured for 24 h. Subsequently, the cells were washed once with PBS and lysed for 5 min in a cold buffer consisting of 50 mM Tris (pH 7.6), 200 mM NaCl, 1 mM EDTA, 0.5% NP40, fresh 0.1 mM DTT (E3876, Sigma), a protease inhibitor cocktail (04693159001, Roche), and a phosphatase inhibitor (No. 524625, Merck). The samples were then sonicated. Cell debris was removed by centrifugation at 16,000 g for 15 min at 4 °C, and 10% of the total cell lysate was preserved as a control for Western blot analysis. Lysates were incubated with 1 μg of antibody for 16 h at 4 °C on a head-over-tail rotator. Subsequently, 20 μL of protein G-Sepharose beads (17-0618-05; GE Healthcare), equilibrated in complete lysis buffer, were added to each sample and gently rotated for 6 h at 4 °C. After this incubation, the samples were washed six times with lysis buffer and then resuspended in 100 μL of Western blot sample buffer. The immunoprecipitation experiments were analysed using Western blotting.

### His-ubiquitin pull-down assay

The His-ubiquitin pull-down assay was performed as described by Radaszkiewicz et al.^51^. Briefly, HEK239 T-REx PRICKLE3 TetON cells were transfected with plasmids encoding polyhistidine-tagged ubiquitin, EGFP-VANGL2, and FLAG-tagged wild-type or enzymatically inactive Mut1 RNF43 constructs. Six hours post-transfection, the culture media were replaced, and PRICKLE3 expression was induced with doxycycline. Twenty hours after transfection, the cells were treated with 5 μM MG-132 (HY-13259, MedChem Express) for 5 h. Subsequently, the cells were lysed in a buffer containing 6 M guanidine hydrochloride (G3272, Sigma), 0.1 M NaxHxPO4 pH 8.0, and 10 mM imidazole (I5513, Sigma), then sonicated and boiled. Samples were centrifuged at 16,000 g for 10 min at room temperature to remove insoluble fractions. Ten percent of the cellular lysate was retained as a transfection control, which was subjected to ethanol precipitation and subsequently resuspended in the Western blot sample buffer. To perform the pull-down of tagged proteins, 10 µL of equilibrated His Mag Sepharose Ni beads (GE28-9799-17, GE Healthcare) were added to each sample and incubated on a roller for 6 h at room temperature. The beads were subsequently washed three times with a buffer composed of 8 M urea (U5378, Sigma), 0.1 M NaH2PO4 (pH 6.3), 0.01 M Tris, and 15 mM imidazole. Following the washes, the beads were resuspended in 100 μL of Western blot sample buffer, boiled for 5 min, and loaded onto an SDS-PAGE gel for further analysis.

### Dual-luciferase TOPFlash/renilla reporter assay

Cells were seeded into 24-well plates at a density of 250,000 cells per well. On the following day, cells were transfected with 150 ng of Firefly luciferase reporter plasmid (Super8X TOPFlash 368) and 150 ng of Renilla luciferase control plasmid (pRLtkLuc 345) per well using 0.3 µL of JetOPTIMUS transfection reagent (Polyplus, #117-07). Transfections were carried out in serum-free medium for 6 h, after which the medium was replaced with fresh complete medium. Where indicated, expression of PRICKLE1 or PRICKLE3 was induced using 0.5 µg/mL doxycycline (DOX). In selected wells, cells were additionally treated with 1 µM LGK-974 (StemRD, #974-02) and/or 100 ng/mL recombinant human WNT3A (R&D Systems, #1324-WN).

After 16 h of stimulation, the medium was removed and cells were frozen at −80 °C until further processing. On the day of measurement, plates were thawed at room temperature and 50 µL of lysis buffer was added to each well. Samples were incubated on a plate shaker for 15 min. Subsequently, 20 µL of lysate was transferred to a 96-well white plate, and 25 µL of Luciferase Assay Reagent II (LAR II) was added to initiate the Firefly luciferase reaction. Luminescence was immediately measured using a Hidex Sense luminometer. Then, Stop & Glo® Reagent was added to the same wells to quench the Firefly signal and initiate the Renilla luciferase reaction, which was also immediately measured.

The reagents and buffers used were from the Dual-Luciferase® Reporter Assay System (Promega, #E1960). Luminescence values are presented as the ratio of TOPFlash to Renilla signals (TOPFlash fold induction), normalized within each experiment to the appropriate control condition. Data were analyzed using Microsoft Excel 2007 and GraphPad Prism 6 and are shown as means ± standard deviation (S.D.). The number of independent experiments is specified in the figure legends.

### Zebrafish experiments

All experiments involving zebrafish were conducted in accordance with protocol BR22-1497 as approved by the Institutional Animal Care and Use Committee (IACUC) of the National University of Singapore.

Adult zebrafish were maintained at 28 °C in recirculating aquaria systems under a 14 h/10 h light/dark cycle in the fish facility of the Department of Biological Sciences (DBS) at National University of Singapore. Wild type (WT) embryos were collected immediately after spawning and transferred to 0.3X Danieau’s solution (17.4 mM NaCl, 0.21 mM KCl, 0.12 mM MgSO_4_, 0.18 mM Ca(NO_3_)_2_, 1.5 mM HEPES, pH = 7.2) for microinjection. Injected embryos were raised in a 28 °C incubator until 80% epiboly, when confocal imaging was performed. Embryonic stages were defined by hours post fertilization (hpf) at 28 °C and morphological features^120^.

To clone the construct for expression of N-terminal tagged mApple-Vangl2, mApple and zebrafish Vangl2, CDS DNA fragments were amplified by Phanta Max high fidelity DNA polymerase (Vazyme). The *pCS2* + *-mApple-vangl2* construct was assembled with *XhoI*- and *XbaI*-co-digested *pCS2* + , *mApple* and zebrafish *vangl2* CDS DNA fragments by CloneExpress II One-step cloning kit (Vazyme).

The mMACHINE SP6 Transcription Kit (ThermoFisher) was used for capped mRNA synthesis with *KpnI*-linearized *pCS105-EGFP-Prickle*^79,80^, *NotI*-linearized *pCS2* + *-mApple-Vangl2* (cloned in this study) and *NotI*-linearized *pcs2* + *-hRNF43-HA*^72^ plasmids as templates, respectively. The final concentrations of synthesized mRNA were measured by Qubit 4 Fluorometer (ThermoFisher) using Qubit™ RNA HS Assay Kit (ThermoFisher).

To study the effect of human RNF43 (hRNF43) on Prickle and Vangl2 localization in WT embryos, 100 pg of *EGFP-Prickle* and 40 pg of *mApple-Vangl2* or 100 pg of *EGFP-Prickle*, 40 pg of *mApple-Vangl2* and 40 pg of *hRNF43-HA* capped mRNAs were co-injected into a single cell of 8-cell stage WT embryos, respectively.

At 80% epiboly, embryos were mounted in 0.5% low-melting agarose in glass bottom imaging dishes. Embryos were oriented with the developing dorsal region facing towards the glass slide of the imaging dish. Single plane fluorescent and DIC images of EGFP-Prickle and mApple-Vangl2 positive cells were captured at comparable Z-depth levels from the enveloping epithelium using a LSM900 confocal microscope (Zeiss) with a 63x (NA = 1.4) oil lens.

To quantitate the relative levels of plasma membrane localized EGFP-Prickle and mApple-Vangl2, two paired regions of interest (ROIs) were drawn on each EGFP-Prickle and mApple-Vangl2 positive cell using ImageJ^121^. Membrane ROIs were selected on cells that had no fluorescent neighboring cell, and the cytoplasm ROIs were selected in regions close to the respective membrane ROI. Line function was used for the membrane ROIs, and circle function for the corresponding cytoplasm ROIs. The mean intensities of paired ROIs were measured, and the relative membrane levels were calculated by dividing the mean intensity of membranous ROI by that of cytoplasmic ROI. To calculate the relative ratio between plasma membrane localized EGFP-Prickle and mApple-Vangl2, the mean intensities of EGFP-Prickle and mApple-Vangl2 were measured using the same membranous ROIs. The relative membranous Prickle level to Vangl2 was calculated by normalizing the mean intensity of membranous EGFP-Prickle to that of membranous mApple-Vangl2.

To compare the effect of hRNF43 on Prickle and Vangl2 localization on membranes, statistical analyses were performed using Mann–Whitney test. *p* values were calculated with a confidence interval of 95%.

### *Xenopus* experiments

All procedures involving *Xenopus laevis* were conducted in accordance with Czech legislation on the use of animals for research and were approved by the relevant institutional and governmental authorities (MSMT-30784/2022 and MSMT-21426/2025, Ministry of Education, Youth and Sports of the Czech Republic; 45055/2020-MZE-18134, Ministry of Agriculture of the Czech Republic; MZP/2025/630/2482, Ministry of the Environment of the Czech Republic).

Embryos were obtained and maintained using standard procedures. Briefly, testes were surgically removed from anesthetized adult males (20% MS-222; Sigma-Aldrich, A5040) and stored in cold 1× Marc’s Modified Ringer’s solution (MMR; 100 mM NaCl, 2 mM KCl, 1 mM MgSO₄, 2 mM CaCl₂, 5 mM HEPES, pH 7.4) supplemented with 50 µg/mL gentamicin (Sigma-Aldrich, G3632). To induce ovulation, sexually mature females were injected with 260 U of human chorionic gonadotropin (hCG; Merck, Ovitrelle 250 G) into the dorsal lymph sac and kept overnight at 18 °C. The following day, eggs were collected by gently squeezing the females and fertilized in vitro using a freshly macerated piece of testis in 0.1× MMR. Fertilized embryos were cultured in 0.1× MMR at 18–21 °C and staged according to^122^.

For expression of target proteins, embryo microinjections were carried out at the 4-cell stage in 3% Ficoll 400 (Cytiva, #17-0300-10). 10 nl of synthetic mRNA encoding the protein of interest at 800 pg were microinjected into all four blastomeres. For Western blot analysis, fifteen animal caps per condition were collected at Nieuwkoop and Faber (NF) stage 9–9.5 and incubated alongside five sibling embryos until they reached stage 10. Both animal caps and whole embryos (WE) were then lysed in buffer containing 50 mM Tris-HCl (pH 7.6), 50 mM NaCl, 1 mM EDTA, 1% Triton X-100, 1× cOmplete™ Protease Inhibitor Cocktail (Roche, #11697498001), 10 mM NaF, 1 mM Na₃VO₄, 25 mM β-glycerol phosphate, and 1 mM PMSF. Lysates were cleared by centrifugation at 15,000 × *g* for 5 min. The resulting supernatant was mixed with 4× SDS sample buffer and subjected to SDS-PAGE followed by Western blotting. Approximately one and a half animal caps’ worth of protein was loaded per lane.

### Protein purification

#### Purification of PRICKLE3 (aa 526–615)

The C-terminal fragment of human PRICKLE3 (residues 526–615) was expressed as a His-ZZ fusion using a pETM11 vector in *E. coli* Rosetta (DE3) cells. Cultures were grown in LB medium supplemented with kanamycin and chloramphenicol, induced with 0.5 mM IPTG at OD < 0.8, and incubated overnight at 16 °C. Cell pellets were lysed in 6 M urea-containing buffer supplemented with protease inhibitors. Following clarification, the soluble fraction was subjected to immobilized metal affinity chromatography (IMAC) using a Co²⁺-charged HiTrap IMAC HP column. The His-ZZ tag was cleaved using TEV protease, followed by dialysis overnight, precipitation of the protease by heat shock, and further purification by ion exchange chromatography (IEX; HiTrap Q HP) and size-exclusion chromatography (SEC; Superdex 75 Increase 10/300 GL). Final aliquots were snap-frozen in liquid nitrogen and stored at −80 °C.

#### Purification of VANGL2 (aa 1–100)

A recombinant N-terminal fragment of human VANGL2 (residues 1–100) was cloned into a pETM11 vector with an N-terminal His–Z tag and expressed in *E. coli* BL21(DE3) cells. Cultures were grown in M9 mineral medium at 37 °C to OD₆₀₀ ~0.7–0.8, and expression was induced with 0.25 mM IPTG for 3–4 h at 37 °C. Cells were harvested and lysed in buffer (25 mM Tris-HCl pH 8.0, 500 mM NaCl, 300 mM sucrose, 10% glycerol, 10 mM imidazole, 1% Triton X-100) supplemented with protease inhibitors (PMSF, AppliChem). Lysates were sonicated (5 s ON / 10 s OFF cycles, amplitude 22, 20 min, 4 °C) and clarified by centrifugation at 27,000 × *g* for 1 h at 4 °C. The supernatant was loaded onto a Co²⁺-charged HiTrap IMAC HP column (Cytiva) and eluted in a gradient to 500 mM imidazole. The fusion tag was removed by TEV protease digestion (1 mg/mL) for 1 h at 25 °C, followed by overnight dialysis at 4 °C into low-salt buffer (25 mM Tris-HCl pH 8.0, 50 mM NaCl, 1 mM EDTA, 5% glycerol). TEV protease was precipitated by heat shock and removed by centrifugation. The sample was further purified by ion exchange chromatography (HiTrap Q HP, Cytiva) and size-exclusion chromatography (Superdex 75 Increase 10/300 GL, Cytiva) in storage buffer (50 mM phosphate buffer pH 6.5, 50 mM KCl). Purified protein was concentrated, snap-frozen in liquid nitrogen, and stored at −80 °C.

### In vitro kinase assay

#### PRICKLE3 full-length and CK1ε

An in vitro kinase assay in Fig. 5e was performed using full-length human CK1ε^123^ and recombinant PRICKLE3 (Antibodies Online, ABIN1316198) as the substrate in a buffer containing 25 mM Tris-HCl (pH 7.5), 250 mM NaCl, 1 mM DTT, 10 mM MgCl2, 1 mM EDTA, and 2 mM ATP. The molar ratio was set to 1:10, with 0.21 μM substrate and 2.1 μM kinase in a 25 μl reaction mix. A sample collected immediately after initiation of the reaction (upon MgCl2 addition) served as the control. The reaction was then carried out for 1 h and overnight (ON). Reactions were stopped by adding 2× Laemmli buffer and boiling at 95 °C for 1 min. Samples were subsequently analysed by WB.

#### VANGL and CK1ε

Kinase reactions in Fig. S5a were carried out in a buffer containing 50 mM Na₂HPO₄, 50 mM NaH₂PO₄, and 50 mM KCl (pH 6.5). For the reaction, 5 µM purified VANGL2 (1–100) and 50 µM PRICKLE3 (526–615) were incubated with 100 nM recombinant CK1ε^123^, 2 mM ATP, and 10 mM MgCl₂. The total reaction volume was 140 µL. Aliquots (20 µL) were collected at 0, 10, 30, 60, and 120 min. Samples were mixed with 2× reducing Laemmli buffer, denatured at 95 °C, and analysed by SDS-PAGE (15% Tris-Glycine gels, 100 V, 2 h). Protein marker was used for molecular weight reference (PageRuler Plus Prestained Protein Ladder, 10 to 250 kDa, ThermoScientific).

### Immunofluorescence and confocal microscopy of tissue culture cells

Cells were plated on glass coverslips that had been coated for 30 min at 37 °C with Cultrex (3536-001-02, Biotechne, R&D Systems) diluted 1:500 in PBS. The cells were fixed with 4% paraformaldehyde (PFA) (1.00196, Sigma-Aldrich), in PBS, followed by permeabilization with 0.1% Triton X-100 in PBS and blocking with a 1% solution of bovine serum albumin (BSA) in PBS. The samples were then incubated overnight at 4 °C with primary antibodies diluted in 1% BSA in PBS. The following day, the cells were incubated with the appropriate Alexa Fluor™ secondary antibodies (Invitrogen) for 1 h at room temperature, also diluted in 1% BSA in PBS. To visualize biotinylation reaction products, streptavidin-Alexa Fluor™ conjugate was utilized. Nuclei were counterstained with 1 μg/mL DAPI (62248, Invitrogen). Finally, the samples were mounted using DAKO medium (S3023, DAKO). Images were acquired using a Leica TCS SP8 confocal laser scanning microscopy platform (Leica).

### Image analysis of the membrane levels of protein

Image analysis was conducted using ImageJ software. To evaluate the membrane levels of proteins, we analyzed the average signal from membrane-associated proteins and normalized it to the average signal from the cytoplasm. Briefly, regions of interest (ROIs) were defined to encompass both the membrane and cytoplasmic areas (see Fig. 5j). We measured the gray value intensity and quantified the mean intensity values for both membrane and cytoplasm. The ratio of these values represented the membrane levels of the protein. Three independent experiments were performed, with gray value intensity measured in at least 40 cells for each condition in every experiment. Statistical analysis of the results and graph preparation were conducted using the SuperPlots of Data tool^124^ available at https://huygens.science.uva.nl/SuperPlotsOfData/.

### miniTurboID samples preparation

A miniTurboID assay was performed according to the published protocol^125^ with minor modifications (details to follow). HEK293 T-REx miniTurboID TetON cell lines were cultured in 15 cm dishes. Once the cells reached confluency, they were treated with doxycycline (1 μg/mL) overnight to induce protein expression. Following doxycycline induction, the cells were stimulated with biotin (50 μM; Santa Cruz Biotechnology) for 6 h. The cells were subsequently washed twice with PBS and lysed on ice for 15 min in a cold lysis buffer consisting of 50 mM Tris (pH 7.6), 500 mM NaCl, 0.4% SDS, and freshly added: 2% Triton X-100, 1 mM DTT (E3876, Sigma), and a protease inhibitor cocktail (04693159001, Roche). The lysates were gently sonicated and incubated on ice for 5 min. Cellular debris was removed by centrifugation at 16,000 g for 15 min at 4 °C. The supernatant was diluted two-fold with 50 mM Tris-HCl (pH 7.4). Ten percent of the total lysate was set aside for subsequent Western blot analysis. The remaining lysates were incubated with Streptavidin Sepharose High-Performance beads (17-5113-01, GE Healthcare) at 4 °C with gentle agitation overnight to capture biotinylated proteins, using 40 μL of bead slurry per sample. After incubation, the samples were washed four times with a wash buffer containing 50 mM Tris (pH 7.6), 250 mM NaCl, 0.2% SDS, and 1% Triton X-100 (freshly added), followed by two additional washes with 50 mM Tris (pH 7.6). Finally, the beads were stored at -80°C for further analysis.

### Mass spectrometry and data analysis (miniTurboID)

Proteins were extracted from the beads using 2% SDS solution for 20 min in a thermomixer (Eppendorf ThermoMixer C, 20 min, 50 °C, 750 rpm). After that, the beads suspension was centrifuged (2 min, 1000 × *g*) and the supernatant was transferred to clean test tube. Dithiothreitol (0.5 M solution in water) was added to the protein solution (final concentration 0.1 M) and proteins were reduced for 15 min at 95 °C. Reduced proteins solution was used for filter-aided sample preparation (FASP) as described elsewhere^126^ using 0.75 μg of trypsin (sequencing grade; Promega). The resulting peptides were extracted into LC-MS vials by 2.5% formic acid (FA) in 50% acetonitrile (ACN) and 100% ACN with the addition of polyethylene glycol (final concentration 0.001%)^127^ and concentrated in a SpeedVac concentrator (Thermo Fisher Scientific).

LC-MS/MS analyses of all peptides were done using UltiMate 3000 RSLCnano system connected to Orbitrap Exploris 480 spectrometer (Thermo Fisher Scientific). Before LC separation, tryptic digests were online concentrated and desalted using a trapping column (Acclaim PepMap 100 C18, dimensions 300 μm ID, 5 mm long, 5 μm particles, Thermo Fisher Scientific). The trap column was then washed with 0.1% FA and the peptides were eluted in backflush mode from the trapping column onto an analytical column tempered to 50 °C (Aurora C18, 3rd generation, 25 cm long, 75 μm ID, 1.7 μm particles, P/N AUR3-25075C18-TSI, Ion Opticks) by 60 min gradient program (flow rate 150 nL.min^-1^, 3-37% of mobile phase B; mobile phase A: 0.1% FA in water; mobile phase B: 0.1% FA in 80% ACN) followed by 7 min long system wash using 80% of mobile phase B. Equilibration of the trapping column and the analytical column was done before sample injection to sample loop. Spray voltage and sweep gas were set to 1.5 kV and 1, respectively.

Data were acquired in a data-independent acquisition mode (DIA). The survey scan covered m/z range of 350-1400 at a resolution of 60,000 (at m/z 200) and a maximum injection time of 55 ms (normalized AGC target 300%). HCD MS/MS (27% relative fragmentation energy) were acquired in the range of m/z 200-2000 at a resolution of 30,000 (maximum injection time 55 ms, normalized AGC target 1000%). Overlapping windows scheme in the precursor m/z range from 400 to 800 were used as isolation window placements – see Supplementary Data 10 for more details. Raw data were converted to mzML format using msconvert (version 3.0.21193-ccb3e0136) using peakPicking (vendor msLevel = 1-) and demultiplex (optimization=overlap_only massError=10ppm) filters applied.

DIA data in mzML format were processed in DIA-NN^128^ (version 1.8.1) in library free mode against a modified cRAP database (based on http://www.thegpm.org/crap/; 111 sequences in total) and UniProtKB protein database for *Homo sapiens* (https://ftp.uniprot.org/pub/databases/uniprot/current_release/knowledgebase/reference_proteomes/Eukaryota/UP000005640/UP000005640_9606.fasta.gz version from 2023-11-08, number of protein sequences: 20,596). No optional, but carbamidomethylation as fixed modification and trypsin/P enzyme with 1 allowed missed cleavage and peptide length 7-30 were set during the library preparation. False discovery rate (FDR) control was set to 1%. MS1 and MS2 accuracies as well as scan window parameters were set (8 ppm, 21 ppm, 9 scans, respectively) based on the initial test searches (median value from all samples ascertained parameter values). MBR was switched on.

The MaxLFQ intensities, reported in the output of DIA-NN, report.tsv were further processed using the software container environment (https://github.com/OmicsWorkflows), version 4.7.7a. The full processing workflow is available on the WorkflowHub under the identifier 1202 with DOI 10.48546/WORKFLOWHUB.WORKFLOW.1202.1. Briefly, it covered a) removal of low-quality precursors and contaminant protein groups, b) protein group intensities log2 transformation and LoessF normalization, c) imputation of missing values by random draws from a manually defined left-shifted Gaussian distribution originated from the actual data with shift of 1.8 and scale 0.3 in a column-wise manner, d) differential expression analysis using LIMMA statistical test calculated for protein groups.

Follow-up analyses were carried out in R, v. 4.4.0 and are available in the GitHub repository: https://github.com/HarnosLab/2024_Radaszkiewicz, with DOI 10.5281/zenodo.14142009. Data visualizations were done using ComplexHeatmap, UpsetR, ggplot2 R packages, gene ontology was done using gProfiler2 (version *e111_eg58_p18_f463989d*) and Metascape tools. SAINT probability score for bait-prey interactions was computed using the REPRINT resource^36^, v. 2.0, with the following settings: experiment type was set to Proximity Dependent Biotinylation; File Type as tab-separated matrix; only user controls were selected. To compute the probabilistic SAINT score, SAINTexpress was used. DotPlots were created using the ProHits-viz suite^129^.

The mass spectrometry proteomics data have been deposited to the ProteomeXchange Consortium via the PRIDE partner repository with the dataset identifier PXD057854.

### Statistics and reproducibility

Statistical significance was determined using two-tailed unpaired Student’s t-tests, linear regression analysis, or, for zebrafish embryo experiments, the Mann–Whitney *U* test. All statistical tests were performed with a 95% confidence interval. Statistical analysis and data visualization were conducted in GraphPad Prism 8.0.

All graphs are presented with error bars showing ± SD, unless stated otherwise in the corresponding figure legends. Sample sizes (n), the definition of n, and the number of biological and technical replicates are reported in each figure legend. Biological replicates refer to independent experiments performed on different days or using different embryo batches, whereas technical replicates refer to repeated measurements within the same experiment.

All key experiments were independently reproduced at least three times, and qualitative observations (e.g., microscopy imaging) were consistently observed across multiple biological replicates. Experiments without formal statistical analysis (e.g., representative immunoblots or imaging panels) were repeated sufficiently to ensure reproducibility, as described in the figure legends.

Sample sizes were not predetermined using statistical methods but were based on commonly accepted standards in the field, our previous work, and established practice in WNT/PCP and developmental biology experiments.

### Reporting summary

Further information on research design is available in the Nature Portfolio Reporting Summary linked to this article.

## Supplementary information

Supplementary_Information

Description of Additional Supplementary Materials

Supplementary_Data_1-10

Supplementary_Data_11

Reporting Summary

## Data Availability

The mass spectrometry proteomics data have been deposited in the ProteomeXchange Consortium via the PRIDE partner repository under the dataset identifier PXD057854. DIA data in mzML format were processed in DIA-NN^128^ (version 1.8.1) in library free mode against a modified cRAP database (based on http://www.thegpm.org/crap/; 111 sequences in total) and UniProtKB protein database for *Homo sapiens* (https://ftp.uniprot.org/pub/databases/uniprot/current_release/knowledgebase/reference_proteomes/Eukaryota/UP000005640/UP000005640_9606.fasta.gz version from 2023-11-08, number of protein sequences: 20,596). All uncropped Western blot images from the main figures are available in Supplementary Fig. 7. Supplementary Data 1–10 contain the Excel source files underlying the mass spectrometry analyses, with detailed description provided in the main text. Supplementary Data 11 provides the Excel source data underlying all graphs presented in the manuscript. All other data supporting the findings of this study are available within the paper or the Supplementary Materials. The human RNF43 plasmid is available from Prof. Tadasuke Tsukiyama under a material transfer agreement (MTA). All other data are available from the corresponding author (or relevant sources, as applicable) upon reasonable request.

## Code availability

The MaxLFQ intensities, reported in the output of DIA-NN, report.tsv were further processed using the software container environment (https://github.com/OmicsWorkflows), version 4.7.7a. The full processing workflow is available on the WorkflowHub under the identifier 1202 with DOI 10.48546/WORKFLOWHUB.WORKFLOW.1202.1. Follow-up analyses were carried out in R, v. 4.4.0 and are available in the GitHub repository: https://github.com/HarnosLab/2024_Radaszkiewicz, with DOI 10.5281/zenodo.14142009. Data visualizations were done using ComplexHeatmap, UpsetR, ggplot2 R packages, gene ontology was done using gProfiler2 (version *e111_eg58_p18_f463989d*) and Metascape tools. SAINT probability score for bait-prey interactions was computed using the REPRINT resource^36^, v. 2.0, with the following settings: experiment type was set to Proximity Dependent Biotinylation; File Type as tab-separated matrix; only user controls were selected. To compute the probabilistic SAINT score, SAINTexpress was used. DotPlots were created using the ProHits-viz suite^129^.
