# Supplementary material for: PRICKLE3 protects VANGL proteins from CK1-mediated phosphorylation and RNF43-mediated degradation
Source: Commun Biol. 2025 Dec 27;9:142. doi: 10.1038/s42003-025-09422-9 (PMC12859043; doi:10.1038/s42003-025-09422-9)
Supplement: Supplementary file 1 — Supplementary_Information [file 42003_2025_9422_MOESM1_ESM.pdf]

Supplementary Information to:

PRICKLE3 protects VANGL proteins from CK1-mediated phosphorylation and RNF43-mediated degradation

Katarzyna A. Radaszkiewicz[1], Tomasz W. Radaszkiewicz[1], Pavla Kolářová[1], Petra Paclíková[1], Kristína Gömöryová[1], Šárka Novotná[1], Lorena Agostini Maia[1], Tereza Číhalová[1], Yao Le[2], Tomáš Bárta[3], Kateřina Hanáková[4], Anna Hýsková[4], Konstantinos Tripsianes[4], Zbyněk Zdráhal[4,5], Christoph Winkler[2], and Jakub Harnoš[1]✉

[1]Department of Experimental Biology, Faculty of Science, Masaryk University, Brno, 62500, Czechia.
[2]Department of Biological Sciences and Centre for Bioimaging Sciences, National University of Singapore, Singapore, 117543, Singapore.
[3]Department of Histology and Embryology, Faculty of Medicine, Masaryk University, Brno, 62500, Czechia.
[4]CEITEC-Central European Institute of Technology, Masaryk University, Brno, 62500, Czechia.
[5]National Centre for Biomolecular Research, Faculty of Science, Masaryk University, Brno, 62500, Czechia.

✉Corresponding author (ORCID 0000-0002-0752-9260), email: harnos@sci.muni.cz

The Supplementary Information contains 7 figures and 3 tables

Supplementary Figure 1

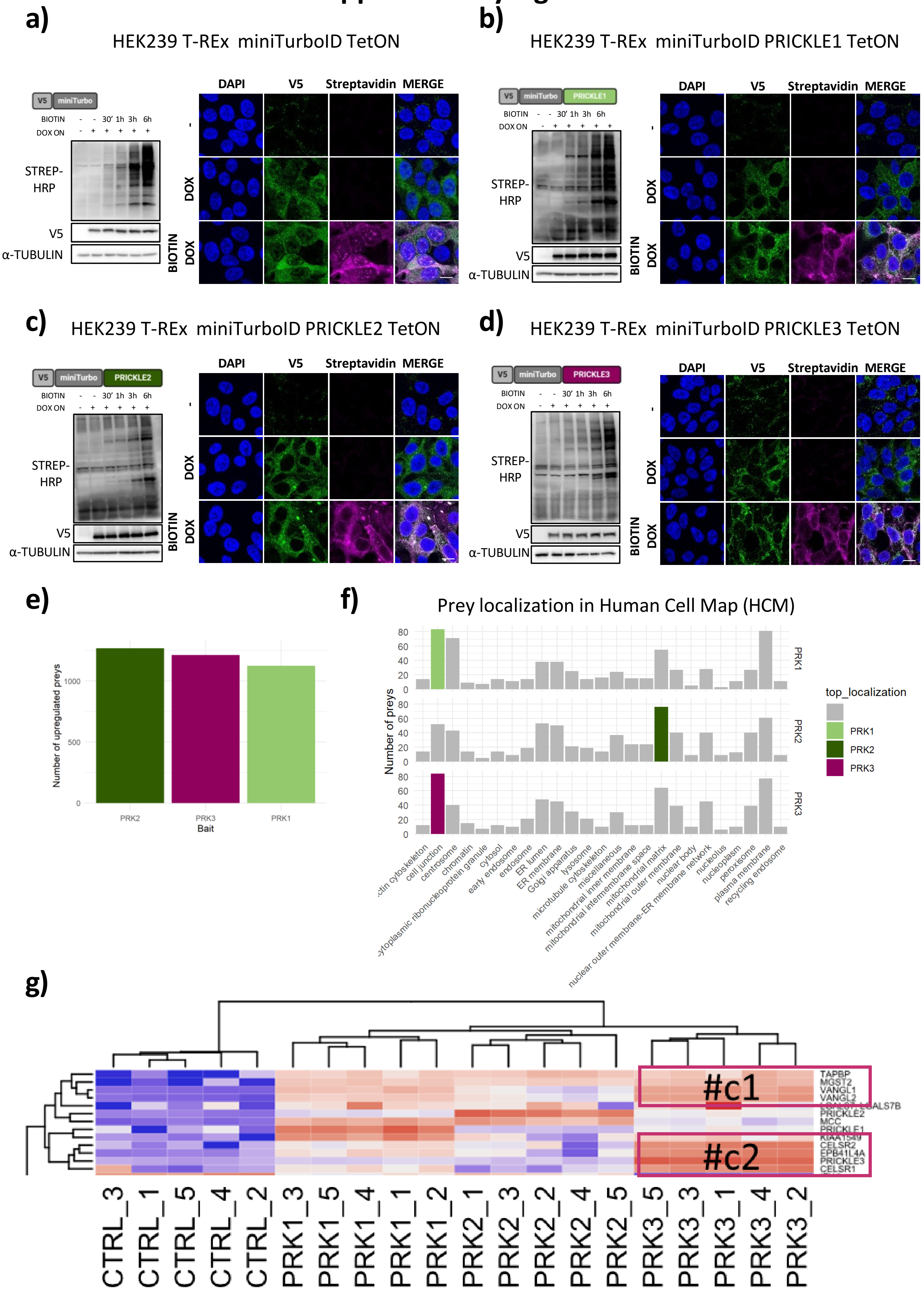

Supplementary Figure 1

continued

h)

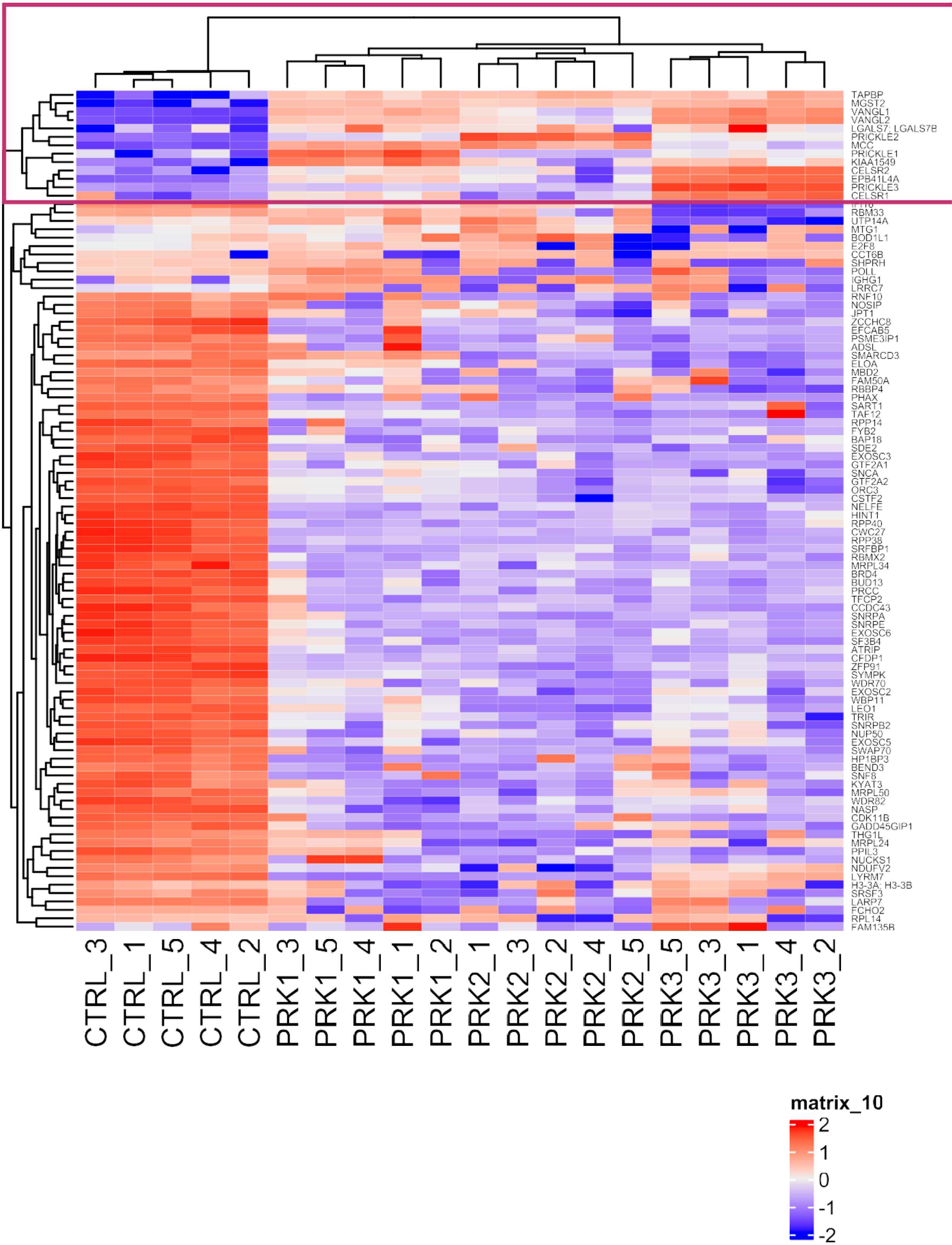

Supplementary Figure 1

continued

i)

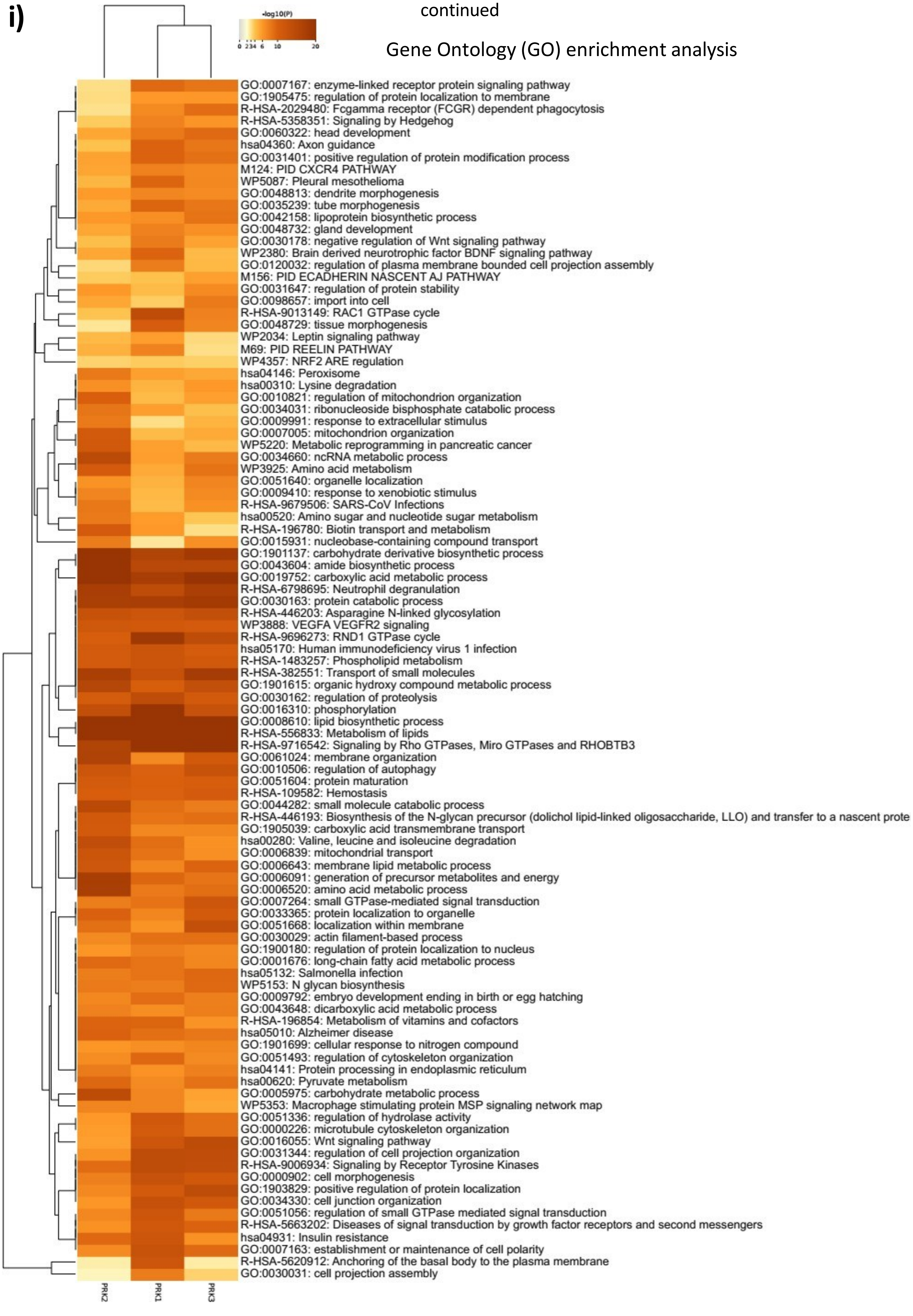

Gene Ontology (GO) enrichment analysis

Supplementary Figure 1

continued

j)

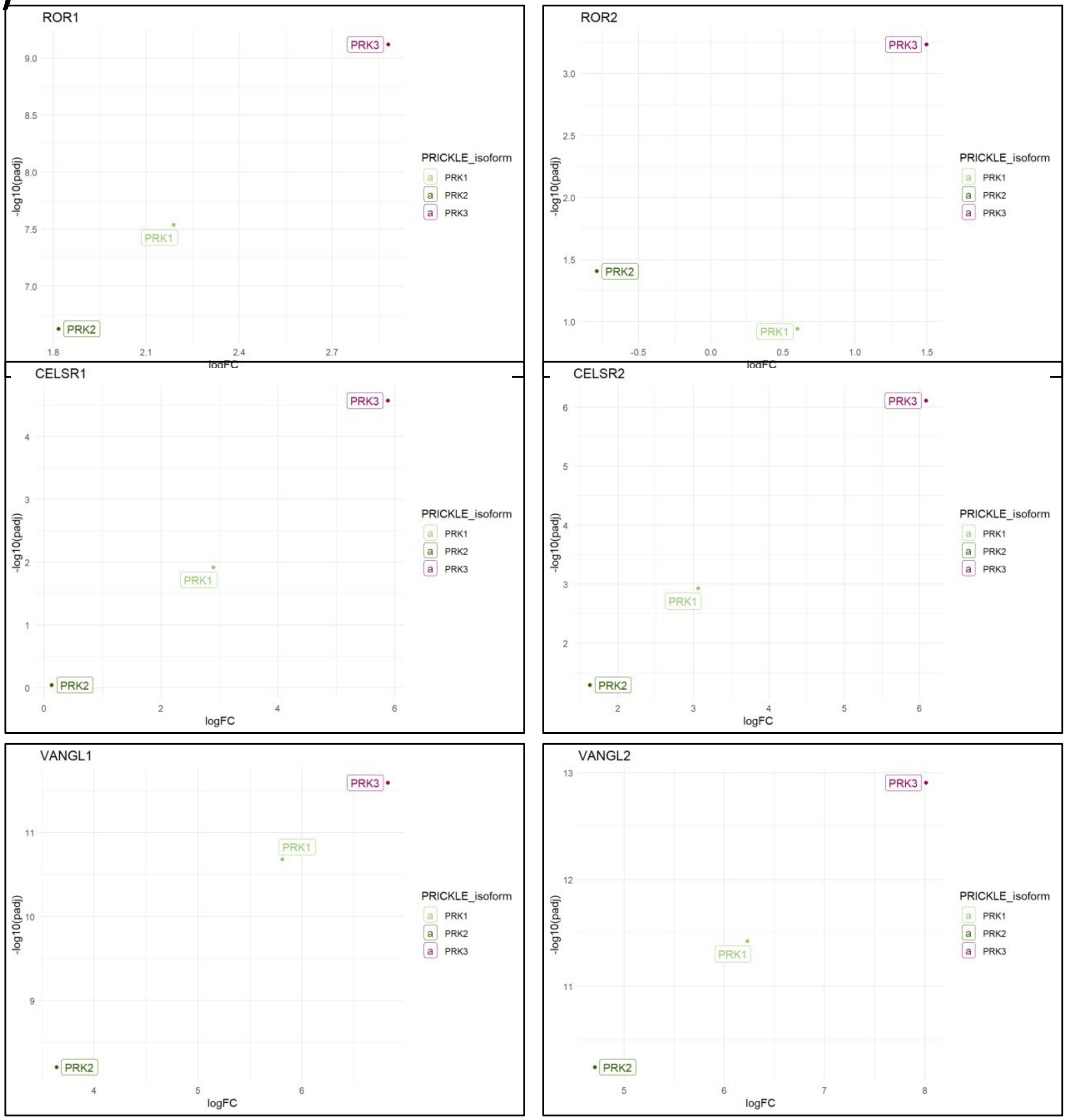

Supplementary Figure 1. PRICKLE3 interactome uncovers specific association with WNT/PCP pathway proteins.

a-d) Validation of HEK T-REx 293 cell lines stably expressing miniTurboID-tagged PRICKLE isoforms: **a)** control (miniTurboID-V5 only), **b)** PRICKLE1, **c)** PRICKLE2, **d)** PRICKLE3. Scale bar, 10 µm.

Constructs encoded human PRICKLE1–3 proteins fused at the N-terminus with miniTurboID and V5 tags. To verify inducible expression and the specificity of the biotinylation reaction, cells were treated overnight with doxycycline (dox ON), followed by the addition of biotin at various time points to initiate proximity labeling. Samples were analyzed by Western blot (WB) for bait expression (anti-V5) and for biotinylated proteins (streptavidin-HRP). α-TUBULIN served as a loading control. Additionally, immunofluorescence was used to assess subcellular localization of the baits under the same treatment. Streptavidin was used to visualize biotinylated proteins, anti-V5 to detect bait expression, and DAPI for nuclear staining.

e) Number of significantly upregulated prey proteins for each bait ($\log_2$ fold change > 1, adjusted p-value < 0.05).

f) Predicted subcellular localization of upregulated prey proteins, based on annotation from the Human Cell Map.

g) Fragment of a heatmap showing proteins upregulated in at least one bait. K-means clustering identified two distinct clusters (#C1 and #C2), both enriched in proteins associated with the WNT/Planar Cell Polarity (PCP) pathway.

h) Full heatmap of proteins upregulated for at least one bait. Proteins highlighted with a violet frame correspond to those shown in Supplementary Figure 1g.

i) Gene Ontology (GO) enrichment analysis of upregulated proteins for each bait.

j) Scatter plot showing BioID results: $\log_2$ fold change (log FC) plotted against $-\log_{10}$(adjusted p-value), for selected prey proteins from the non-canonical WNT signalling, which are membrane-associated.

Supplementary Figure 2

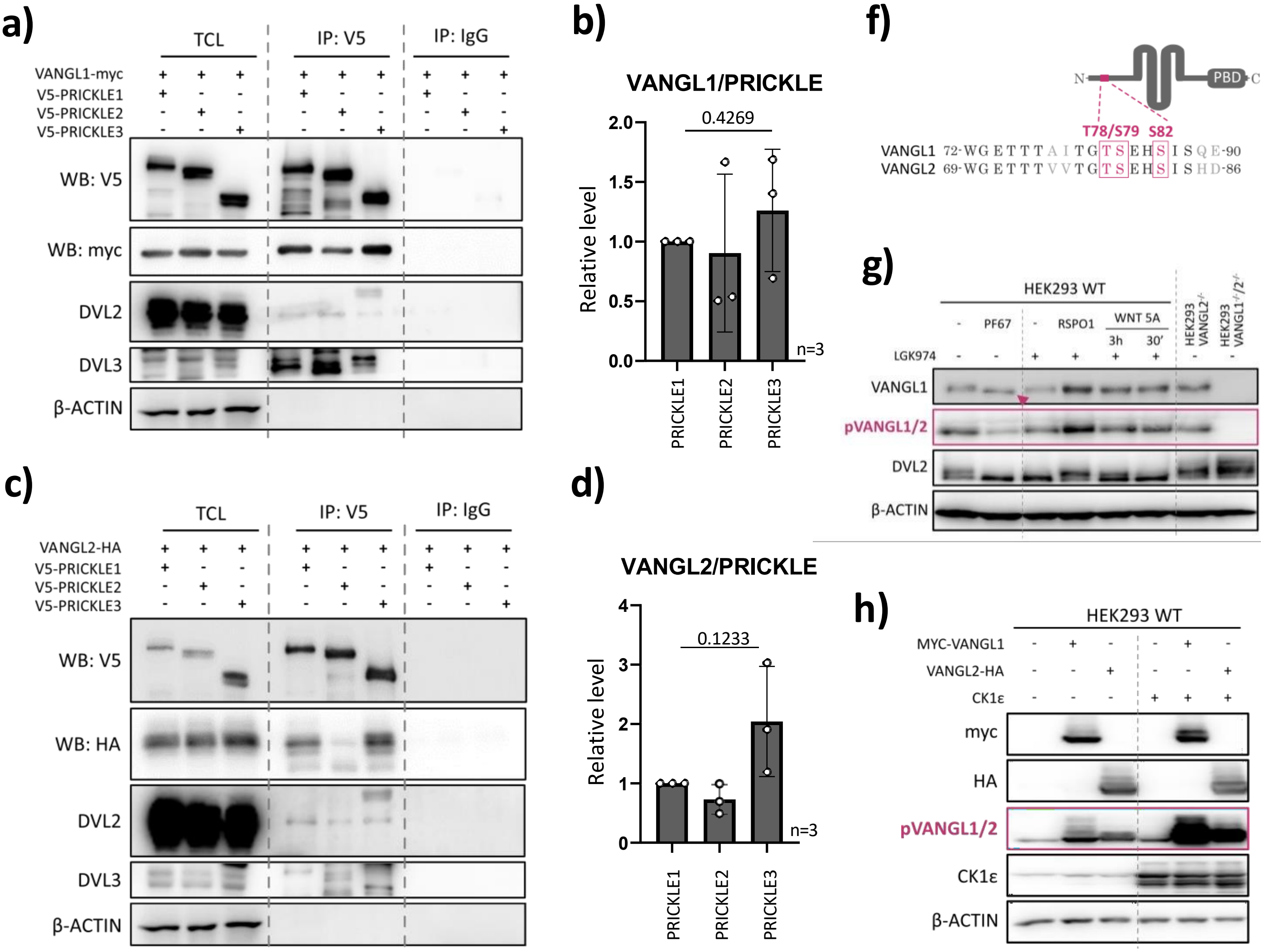

e)

Proximity biotinylation (intensity-based)

Protein hits:	PRICKLE1 logFC	PRICKLE1 adj.P.Val	PRICKLE2 logFC	PRICKLE2 adj.P.Val	PRICKLE3 logFC	PRICKLE3 adj.P.Val
DVL1	0.5093	0.1112	0.0560	0.8619	1.4453	0.0002
DVL2	0.5609	0.0550	0.6533	0.0254	0.4818	0.0938
DVL3	0.7784	0.0010	0.7987	0.0007	1.4572	0.0001

Supplementary Figure 2. PRICKLE3 contains functional VANGL-binding motives and interacts with VANGL1/2.

a) Co-immunoprecipitation of overexpressed VANGL1-myc with V5-tagged PRICKLE1–3 from HEK293 cells. Immunoprecipitation (IP) was performed using anti-V5 to pull down V5-PRICKLE proteins. PRICKLE3 showed the strong interaction with VANGL1. Endogenous Dishevelled2/3 (DVL2/3) co-precipitation was also observed, indicating additional binding to the PRICKLE complex. **b)** Quantification of the VANGL1–PRICKLE co-immunoprecipitation signal, providing an approximate comparison between isoforms, n=3. Statistical analysis was performed using an unpaired t-test.

c) Co-immunoprecipitation of overexpressed VANGL2-HA with V5-tagged PRICKLE1–3 from HEK293 cells. IP was performed using anti-V5 as above. PRICKLE3 again showed strong association with VANGL2. Endogenous DVL2/3 binding was also detected. **d)** Quantification of the VANGL2–PRICKLE co-immunoprecipitation signal, providing a rough estimate of relative interaction strength, n=3. Statistical analysis was performed using an unpaired t-test.

e) Table summarizing BioID results for Dishevelled1–3 (DVL1–3) proteins, showing $\log_2$ fold change ($\log_2$FC) and $-\log_{10}$(adjusted p-value). Values with $\log_2FC > 1.00$ and $p < 0.05$ are highlighted in bold.

f) Sequence alignment of VANGL1 and VANGL2 in the region corresponding to the epitope recognized by the phospho-specific pVANGL1/2 antibody. Three conserved CK1ε phosphorylation sites are highlighted in pink. Conserved residues are shown in dark grey; non-conserved residues in light grey. PBD = PRICKLE-binding domain.

g) Anti-VANGL antibody validation at the endogenous level. HEK T-REx 293 wild-type (WT) cells were pre-treated overnight with the LGK974 porcupine inhibitor to block WNT ligand secretion and subsequently stimulated with 100 ng/ml recombinant human WNT5A for either 30 minutes or 3 hours, 25 ng/ml R-Spondin1 (RSPO1), or 2.5 µM CK1ε/δ inhibitor PF-670462 (PF67) for 3 hours. HEK T-REx 293 cells lacking VANGL2 (KO) or both VANGL1 and VANGL2 (double KO) used as negative controls to assess antibody specificity. β-ACTIN used as a loading control.

h) Validation of anti-VANGL antibody specificity in an overexpression model. HEK T-REx 293 wild-type (WT) cells were transfected with plasmids encoding MYC-tagged VANGL1, HA-tagged VANGL2, and CK1ε. Protein expression and detection were confirmed by Western blotting.

Supplementary Figure 3

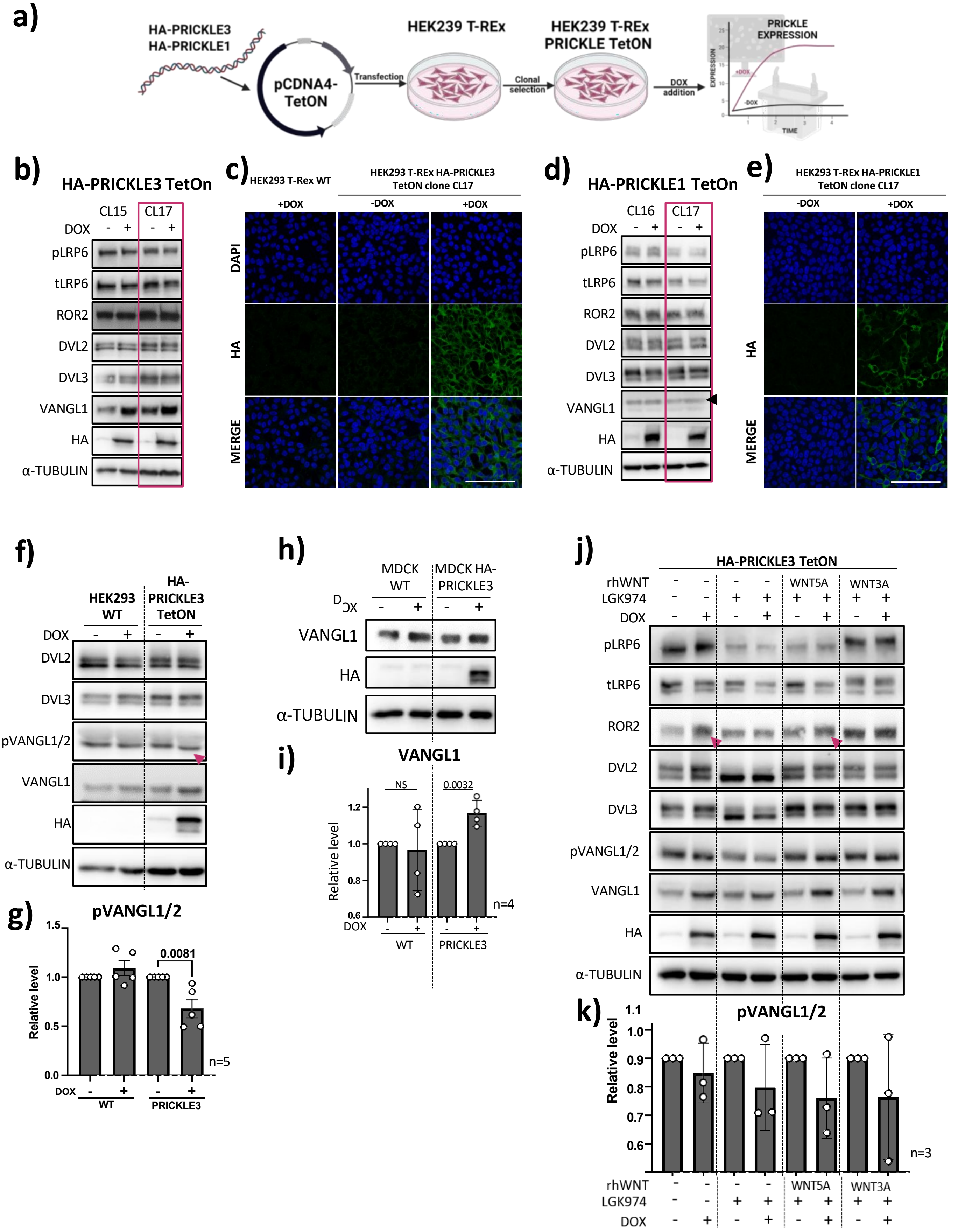

Supplementary Figure 3

continued

Supplementary Figure 3. PRICKLE3 enhances VANGL protein stability and promotes ROR2 electrophoretic shifts.

a) Generation of stable, clonal, doxycycline-inducible HEK T-REx 293 cell lines expressing N-terminally HA-tagged PRICKLE1 or PRICKLE3. Wild-type HEK T-REx 293 cells were transfected with the respective constructs, followed by single-cell cloning and validation of inducible expression. Created with BioRender.com

b) Validation of HA-PRICKLE3-inducible clones. Upon doxycycline induction, expression of HA-PRICKLE3 and representative proteins from both canonical and non-canonical WNT signaling pathways was analyzed by Western blot. α-TUBULIN was used as a loading control. Clone 17 was selected for further experiments.

c) Immunofluorescence analysis of HA-PRICKLE3 expression in the doxycycline-inducible cell line. Cells were stained with anti-HA antibody to confirm inducible expression, and nuclei were counterstained with DAPI. Wild-type HEK T-REx 293 cells served as a control for anti-HA antibody specificity. Scale bar, 100 µm.

d) Validation of HA-PRICKLE1-inducible clones. Expression of HA-PRICKLE1 and selected WNT pathway proteins was confirmed by Western blot upon doxycycline treatment. α-TUBULIN served as a loading control. Clone 17 was selected for downstream analysis.

e) Immunofluorescence analysis of HA-PRICKLE1-expressing cells. Cells were stained with anti-HA antibody, and nuclei were counterstained with DAPI. Scale bar, 100 µm.

f) Inducible overexpression of HA-PRICKLE3 in HEK T-REx 293 cells. Wild-type cells treated with doxycycline served as controls. Arrowheads indicate electrophoretic mobility shift of VANGL1/2 due to phosphorylation. α-TUBULIN was used as a loading control. Representative result from n = 4.

g) Densitometric quantification of pVANGL1/2 Western blot bands from panel f. Signal intensities were normalized to untreated cells. Statistical significance was assessed using an unpaired t-test; n = 5.

h) Inducible overexpression of HA-PRICKLE3 in MDCK TetON cells. Wild-type MDCK cells treated with doxycycline served as controls. α-TUBULIN served as a loading control. Representative result from n = 4.

i) Densitometric quantification of VANGL1 Western blot signals from panel h. Results were normalized to untreated cells. Statistical significance was calculated using an unpaired t-test; n = 4.

j) Effect of recombinant WNT stimulation on VANGL phosphorylation. HEK T-REx 293 TetON-PRICKLE3 cells were pretreated overnight with the porcupine inhibitor LGK-974 to block endogenous WNT ligand secretion and then stimulated with 100 ng/ml recombinant human WNT5A or WNT3A for 3 hours. Arrowheads indicate mobility shifts of VANGL due to phosphorylation. α-TUBULIN served as a loading control. Representative result from n = 3.

k) Densitometric quantification of Western blot signals for pVANGL1/2 from panel j. Intensities were normalized to untreated controls. Statistical analysis was performed using an unpaired t-test; n = 3. No statistical significance was observed.

Supplementary Figure 4

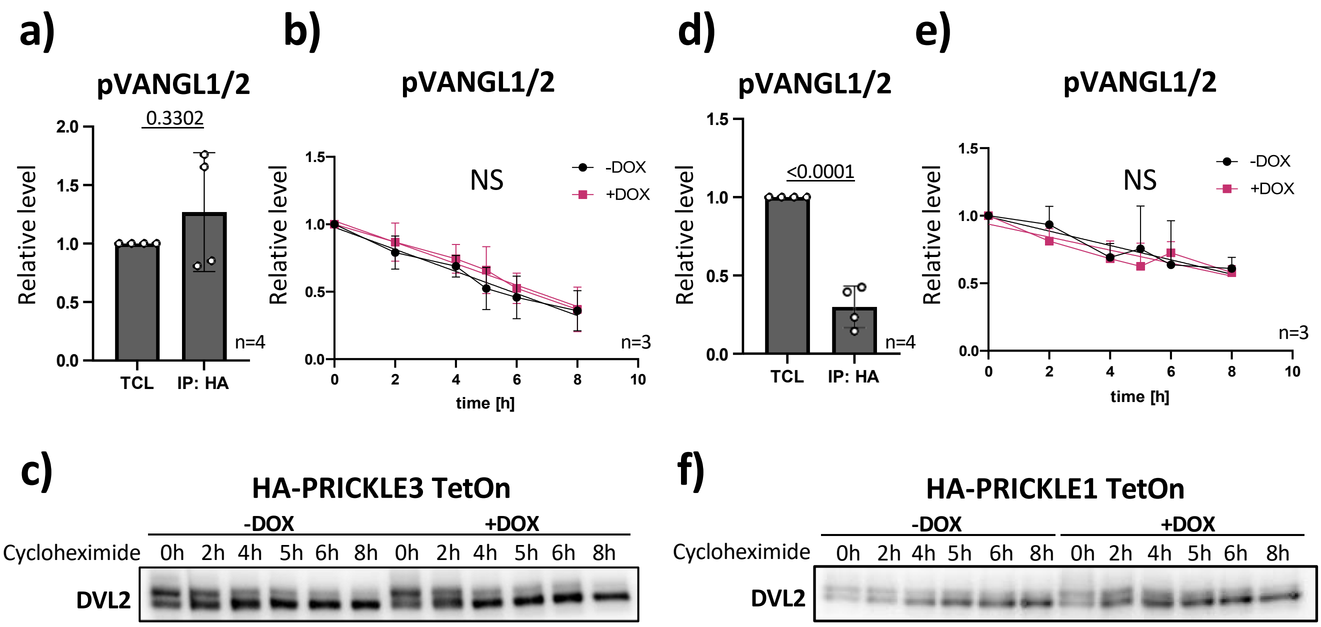

Supplementary Figure 4. PRICKLE3, but not PRICKLE1, stabilizes VANGL by protecting it from degradation.

a) Densitometric quantification of phosphorylated VANGL1/2 (pVANGL1/2) signal from Figure 4a (PRICKLE3 immunoprecipitation). Band intensities were normalized to untreated controls. Statistical significance was assessed using an unpaired *t*-test; *n* = 4.

b) Densitometric quantification of pVANGL1/2 Western blot signal from Figure 4d, representing PRICKLE3 protein stability measured by cycloheximide (CHX) pulse-chase assay in HEK T-REx 293 PRICKLE3 TetON cells. Intensities were normalized to the 0-hour time point. Linear regression was used to evaluate statistical significance; corresponding *p*-values are shown. (NS) indicates no statistically significant difference; *n* = 3.

c) Western blot analysis of DVL2 levels from the CHX pulse-chase assay shown in Figure 4d. HA-PRICKLE3 expression was induced by overnight doxycycline treatment in HEK T-REx 293 PRICKLE3 TetON cells.

d) Densitometric quantification of pVANGL1/2 signal from Figure 4g (PRICKLE1 immunoprecipitation). Band intensities were normalized to untreated controls. Statistical analysis was performed using an unpaired *t*-test; *n* = 4.

e) Densitometric quantification of pVANGL1/2 signal from Figure 4j, representing PRICKLE1 protein stability determined by CHX pulse-chase assay in HEK T-REx 293 PRICKLE1 TetON cells. Intensities were normalized to the 0-hour time point. Linear regression was used for statistical analysis; *p*-values are shown. (NS) denotes no statistically significant difference; *n* = 3.

f) Western blot analysis of DVL2 levels from the CHX pulse-chase assay shown in Figure 4j. HA-PRICKLE1 expression was induced by overnight doxycycline treatment in HEK T-REx 293 PRICKLE1 TetON cells.

Supplementary Figure 5

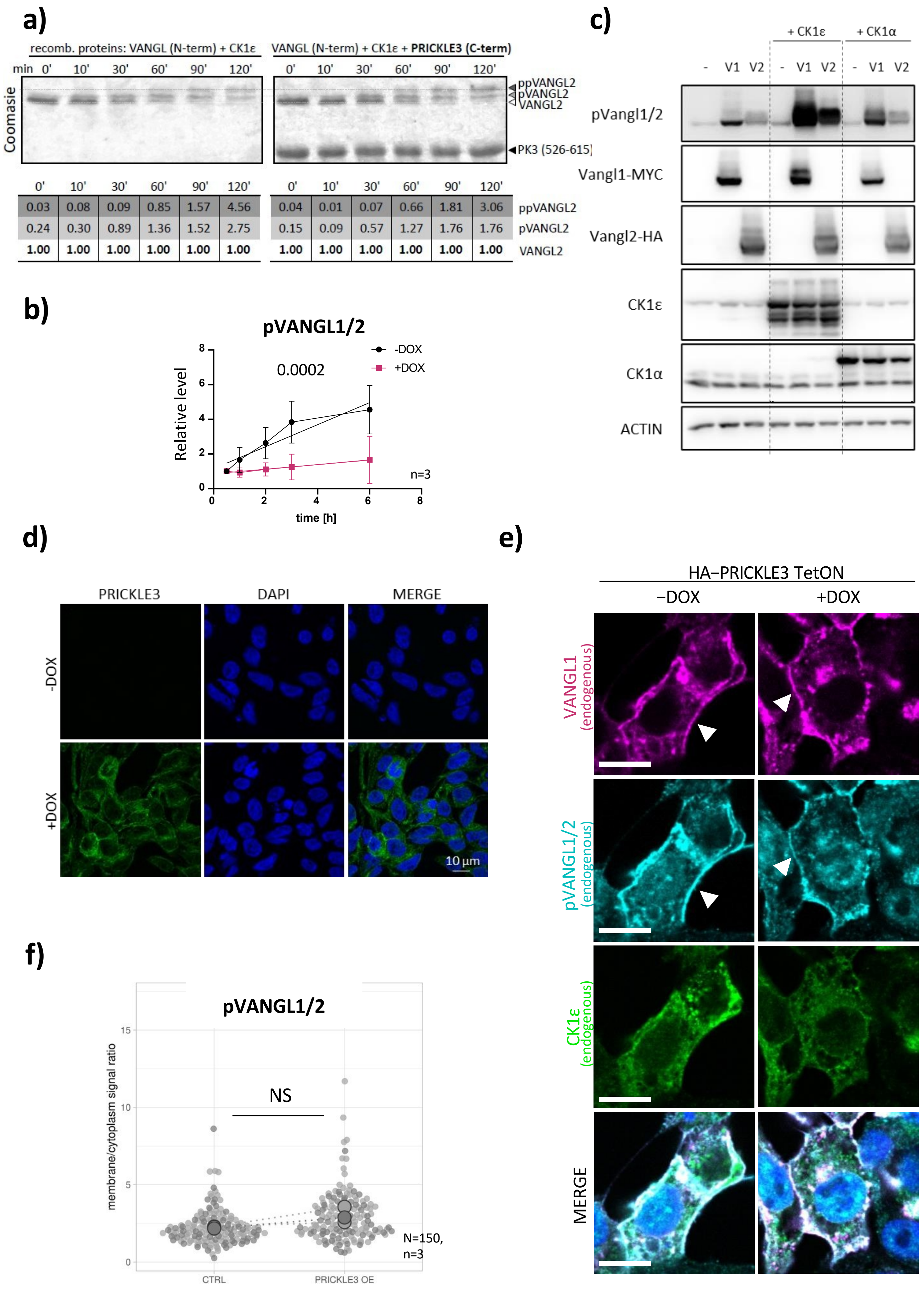

0'	10'	30'	60'	90'	120'	
0.03	0.08	0.09	0.85	1.57	4.56	ppVANGL2
0.24	0.30	0.89	1.36	1.52	2.75	pVANGL2
1.00	1.00	1.00	1.00	1.00	1.00	VANGL2

0'	10'	30'	60'	90'	120'	
0.04	0.01	0.07	0.66	1.81	3.06	ppVANGL2
0.15	0.09	0.57	1.27	1.76	1.76	pVANGL2
1.00	1.00	1.00	1.00	1.00	1.00	VANGL2

Supplementary Figure 5
continued

Supplementary Figure 5. PRICKLE3 reduces CK1ε-mediated phosphorylation of VANGL.

a) *In vitro* kinase assay using human recombinant proteins. Coomassie-stained gel showing the phosphorylation of N-terminal VANGL2 (aa 1–100) by CK1ε in the presence or absence of PRICKLE3 C-terminal fragment (aa 526– 615). The addition of PRICKLE3 slowed down CK1ε-mediated phosphorylation of VANGL2. The reaction was carried out at six time points. Densitometric quantification of phosphorylated (p and pp) versus non- phosphorylated VANGL2 is shown below.

b) Densitometric quantification of pVANGL1 signal from the Western blot. Band intensities were normalized to the 1-hour time point. Statistical analysis was performed using linear regression; (NS) indicates no statistically significant difference. N = 5. Data correspond to the experiment shown in Figure 5F (Pf67 treatment in PRICKLE3- inducible HEK293 cells).

c) Overexpression of CK1α and CK1ε kinases to test their ability to phosphorylate VANGL proteins. Both isoforms were capable of inducing pVANGL1/2 to some extent, but CK1ε more. HA-VANGL2 and myc-VANGL1 constructs were used for detection.

d) Immunofluorescence analysis of PRICKLE3 expression in inducible HEK293 cells. Cells were stained with anti- HA antibody to visualize HA-tagged PRICKLE3. Data correspond to Figure 5i. Scale bar, 10 µm.

e) Immunofluorescence imaging of membrane levels of VANGL1 and pVANGL2 in the presence of PRICKLE3. HEK T-REx 293 PRICKLE3 TetON cells were transfected with MYC-VANGL1 and CK1ε constructs. PRICKLE3 expression was induced overnight by treatment with doxycycline**.** Data correspond to Figure 5i. Scale bar, 10 µm.

f) Quantification of the membrane-localized vs. cytoplasm pVANGL1/2 signal in PRICKLE3-inducible cells (related to Figure 5i). Data were analyzed using the SuperPlots of Data tool. (NS) indicates no statistically significant difference. N = 150 cells from n = 3 biological replicates; individual replicates are shown in different shades of gray.

Supplementary Figure 6

a)

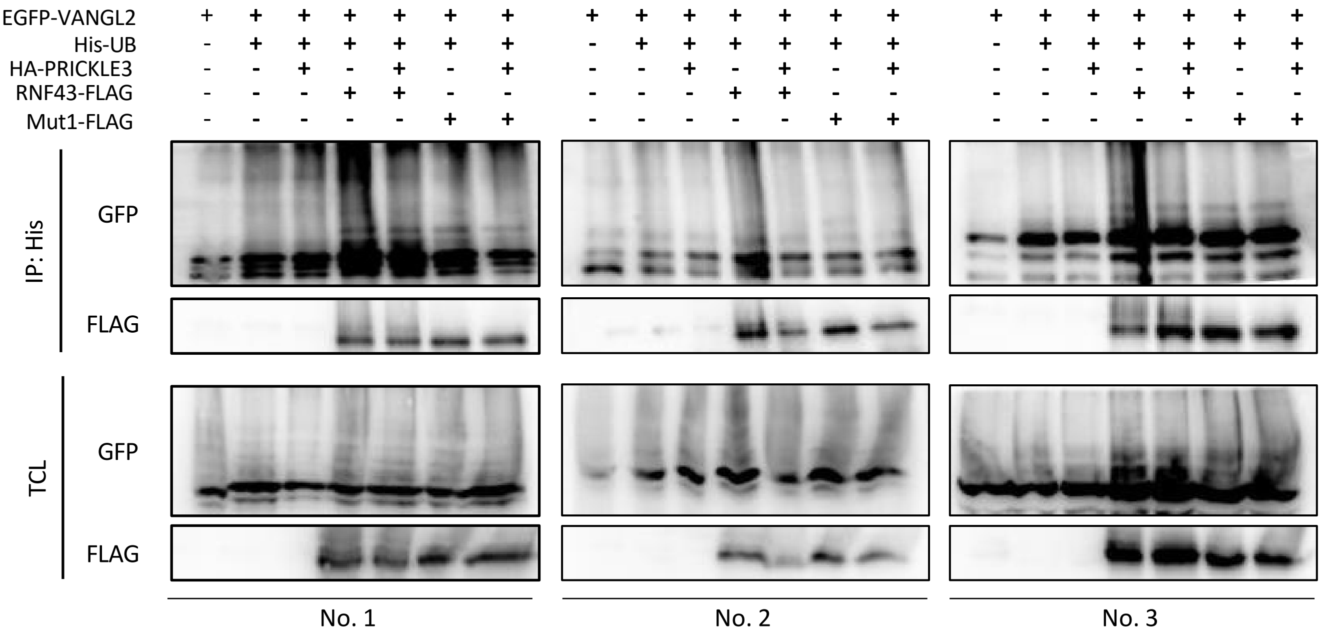

b)

```
PRICKLE3_WT       ATGTTCGCGCGTGGGTCCCGGAGGCGCCGCTCCGGGCGTGCGCCTCCAGAGGCAGAGGAC   60
CL13              ATGTTCGCGCGTGGGTCCCGGAGGCGCCGCTCCGGGCGTGCGCCTCCAGAGGCAGAGGAC   60
CL27              ATGTTCGCGCGTGGGTCCCGGAGGCGCCGCTCCGGGCGTGCGCCTCCAGAGGCAGAGGAC   60
CL39_insertion    ATGTTCGCGCGTGGGTCCCGGAGGCGCCGCTCCGGGCGTGCGCCTCCAGAGGCAGAGGAC   60
CL39_deletion     ATGTTCGCGCGTGGGTCCCGGAGGCGCCGCTCCGGGCGTGCGCCTCCAGAGGCAGAGGAC   60
                  ************************************************************

PRICKLE3_WT       CCAGACCGGGGCCAGCCCTGCAACTCCTGTAGGGAGCAGTGCCCTGGCTTCCTGCTCCAC   120
CL13              CCAGACCGGGGCCAGCCCTGCAACTCCTGTAGGGAGCAGTGCCCTGGCTTCCTGCTCCAC   120
CL27              CCAGACCGGGGCCAGCCCTGCAACTCCTGTAGGGAGCAGTGCCCTGGCTTCCTGCTCCAC   120
CL39_insertion    CCAGACCGGGGCCAGCCCTGCAACTCCTGTAGGGAGCAGTGCCCTGGCTTCCTGCTCCAC   120
CL39_deletion     CCAGACCGGGGCCAGCCCTGCAACTCCTGTAGGGAGCAGTGCCCTGGCTTCCTGCTCCAC   120
                  ************************************************************

PRICKLE3_WT       GGCTGGAGAAAGATCTGCCAGCATTGCAAATGCCCGCGGGAGGAGCATGCAGTGCACGCG   180
CL13              GGCTGGAGAAAGATCTGCCAGCATTGCAAATGCCCGCGGGAGGAGCATGCAGTGCACGCG   180
CL27              GGCTGGAGAAAGATCTGCCAGCATTGCAAATGCCCGCGGGAGGAGCATGCAGTGCACGCG   180
CL39_insertion    GGCTGGAGAAAGATCTGCCAGCATTGCAAATGCCCGCGGGAGGAGCATGCAGTGCACGCG   180
CL39_deletion    GGCTGGAGAAAGATCTGCCAGCATTGCAAATGCCCGCGGGAGGAGCATGCAGTGCACGCG    180
                 ************************************************************

PRICKLE3_WT       GTGCCTGTGGACCTGGAACGCATCATGTGTCGGCTAATCTCGGACTTCCAGCGCCACTCC   240
CL13              GTGCCTGTGGACCTGGAACGCATCTTGTGTCGGCTAATCTCGGACTTCCAGCGCCACTCC   240
CL27              GTGCCTGTGGACCTGGAACGCATCATGTGTCGGCTAATCTCGGACTTCCAGCGCCACTCC   240
CL39_insertion    GTGCCTGTGGACCTGGAACGCATCATGTGTCGGCTAATCTCGGACTTCCAGCGCCACTCC   240
CL39_deletion    GTGCCTGTGGACCTGGAACGCATCATGTGTCGGCTAATCTCGGACTTCCAGCGCCACTCC    240
                 ************************ ***********************************

PRICKLE3_WT       ATCTCCGACGACGA-CTCAGGCTGTGCATCGGAGGAGTATGCCTGGGTGCCCCCAGGCCT   299
CL13              ATCTCCGACGACGAGCTCAGGCTGTGCATCGGAGGAGTATGCCTGGGTGCCCCCAGGCCT   300
CL27             ATCTCCGACGACGAGA ----- TGTGCATCGGAGGAGTATGCCTGGGTGCCCCCAGGCCT   294
CL39_insertion    ATCTCCGACGACGAACTCAGGCTGTGCATCGGAGGAGTATGCCTGGGTGCCCCCAGGCCT   300
CL39_deletion    ATCATCTCCGACGACATCAGGCTGTGCATCGGAGGAGTATGCCTGGGTGCCCCCAGGCCT    300
                 ***  *  ******         *************************************

PRICKLE3_WT       TAAGCCGGAGCAGGTATATCAATTTTTCAGCTGCCTCCCAGAGGACAAGGTCCCCTACGT   359
CL13              TAAGCCGGAGCAGGTGACCAGAGGCCAGCCCTCCACTTTGC-------------------   341
CL27              TAAGCCGGAGCAGGTGACCAGAGGCCAGCCCTCCAC------------------------   330
CL39_insertion   ------------------------------------------------------------    300
CL39_deletion    ------------------------------------------------------------    300
```

Supplementary Figure 6
continued

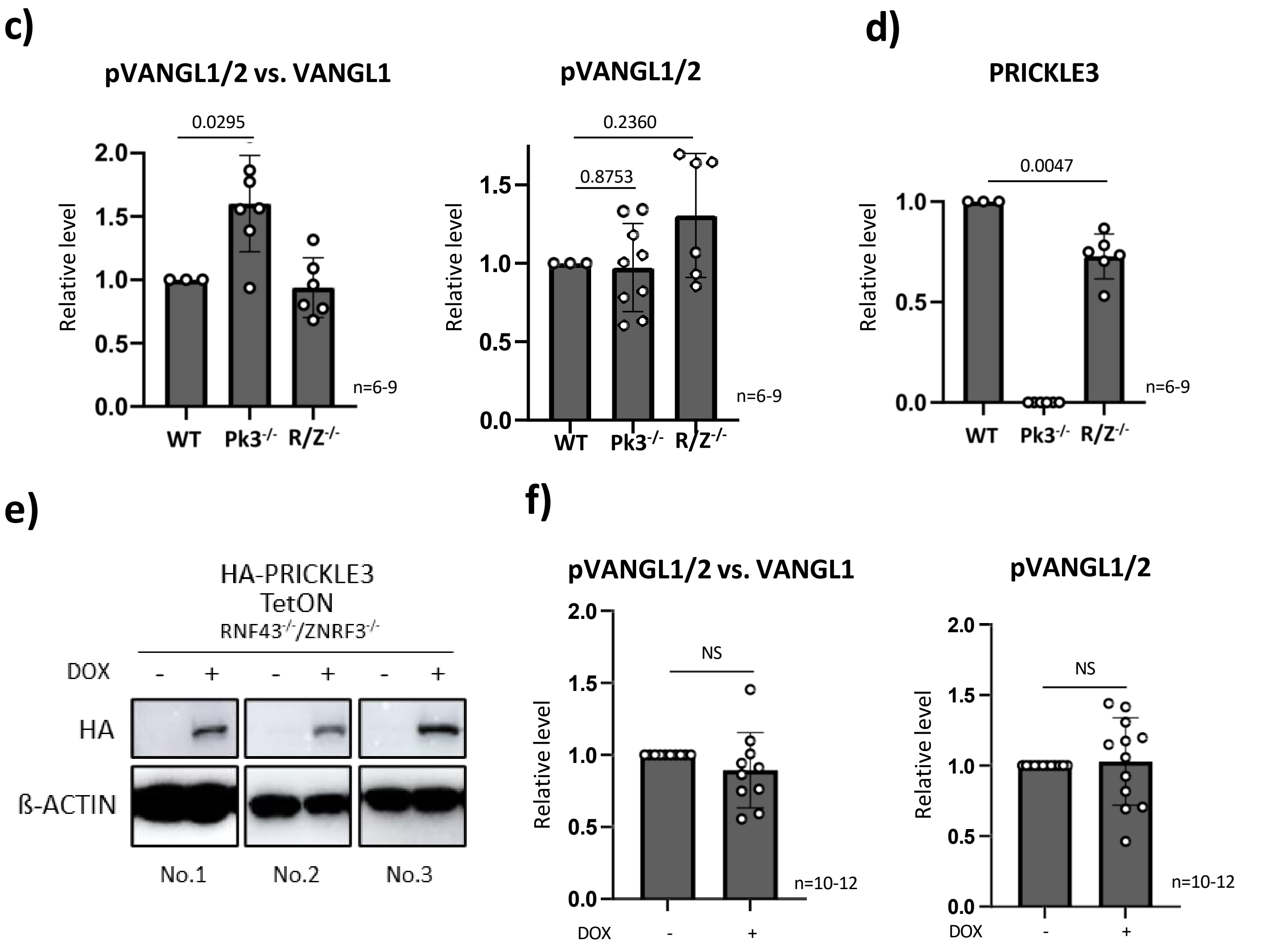

Supplementary Figure 6. PRICKLE3 protects VANGL from RNF43-mediated ubiquitination.

a) Ubiquitination assay in HEK T-REx 293 PRICKLE3 TetON cells. Cells were transfected with His-tagged ubiquitin, EGFP- VANGL2, and FLAG-tagged RNF43 constructs (wild-type or enzymatically inactive mutant Mut1). PRICKLE3 expression was induced by overnight doxycycline treatment. Ubiquitinated proteins were enriched via His pull-down and analyzed by Western blot. Total cell lysate (TCL) was used as input control. Three repeats are shown, and data corresponds to Figure 6c.

b) Alignment of PRICKLE3 exon 1 sequences (~first 300 nucleotides) from three independent CRISPR-Cas9-engineered PRICKLE3-deficient HEK293 clones (#13, #27, and #39). All clones carry frameshift-inducing mutations at the sgRNA target site. Clones #13 and #27 show uniform biallelic edits (insertion or deletion), while clone #39 displays a mixed sequence pattern consistent with compound heterozygosity: one allele carrying an insertion and the other a deletion. These indels disrupt the open reading frame and are predicted to abolish PRICKLE3 expression. The knockout status of all three clones was independently validated by Western blotting (Figure 6h).

c) Densitometric quantification of Western blot signals from Figure 6H for CRISPR-Cas9 knockout cells. Ratios of phosphorylated VANGL1/2 (pVANGL1/2) to total VANGL1 and pVANGL1/2 levels alone were quantified. Intensities were normalized to untreated controls. Statistical analysis was performed using an unpaired *t*-test; *n* = 6-9.

d) Quantification of PRICKLE3 levels from the same Western blot in Figure 6h for CRISPR-Cas9 knockout cells. Intensities were normalized to untreated controls. Statistical analysis was performed using an unpaired *t*-test; *n* = 6-9.

e) Representative images of three independent cell clones with DOX-induced PRICKLE3 expression in an *RNF43/ZNRF3- deficient* background. β-ACTIN was used as a loading control.

f) Densitometric quantification of pVANGL1/2 to VANGL1 and pVANGL1/2 levels from Figure 6j for cells with induced PRICKLE3 expression in an *RNF43/ZNRF3*-deficient background. Intensities were normalized to untreated controls. Statistical analysis was performed using an unpaired *t*-test; *n* = 10-12.

Supplementary Figure 7

Uncropped Western blots

Fig. 2c

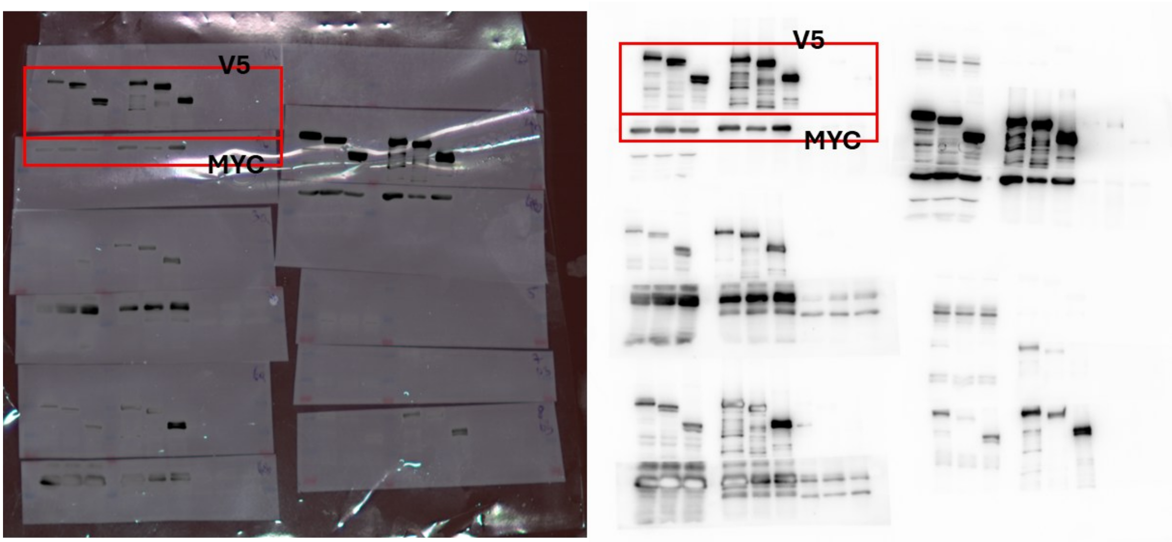

Fig. 2d

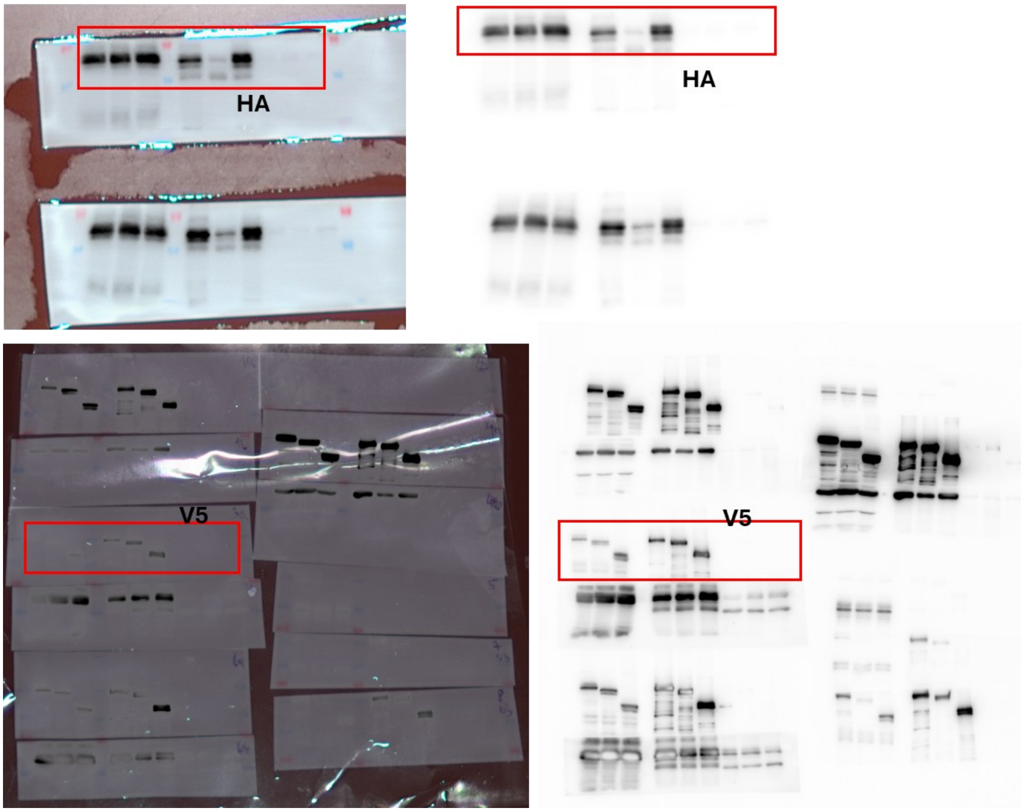

Supplementary Figure 7

continued

Fig. 3b

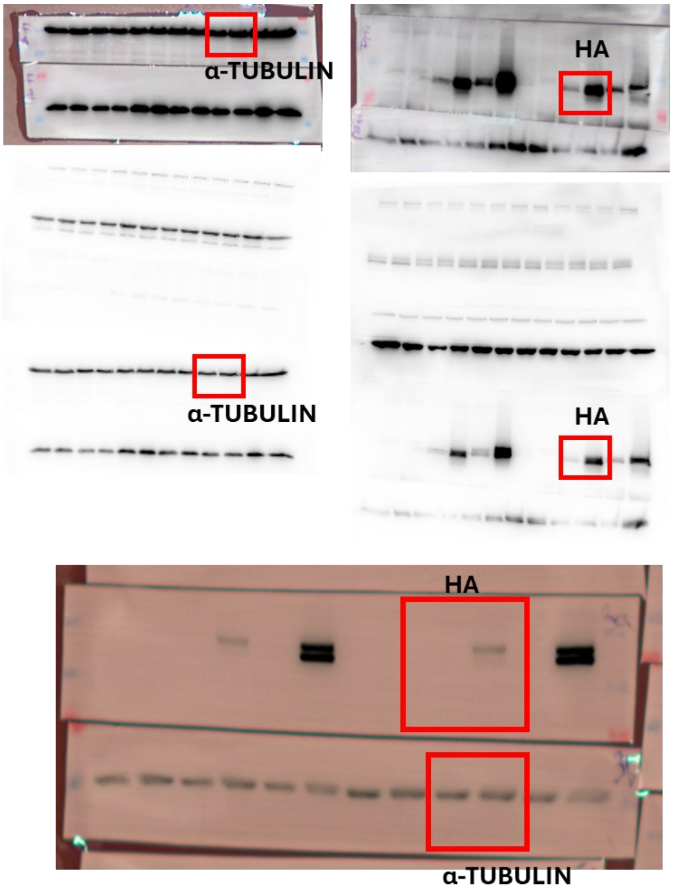

Fig. 3c

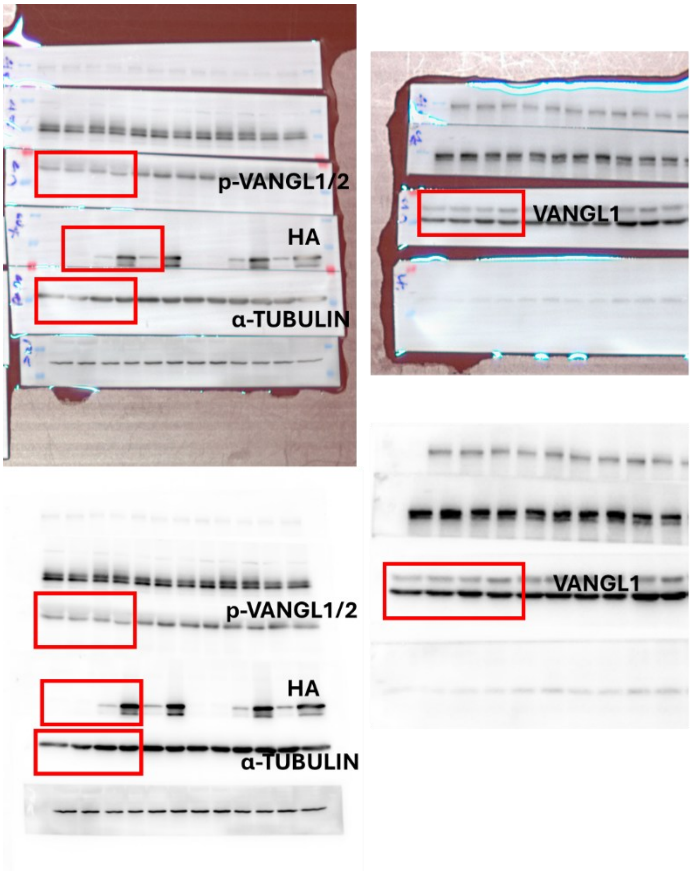

Fig. 3f

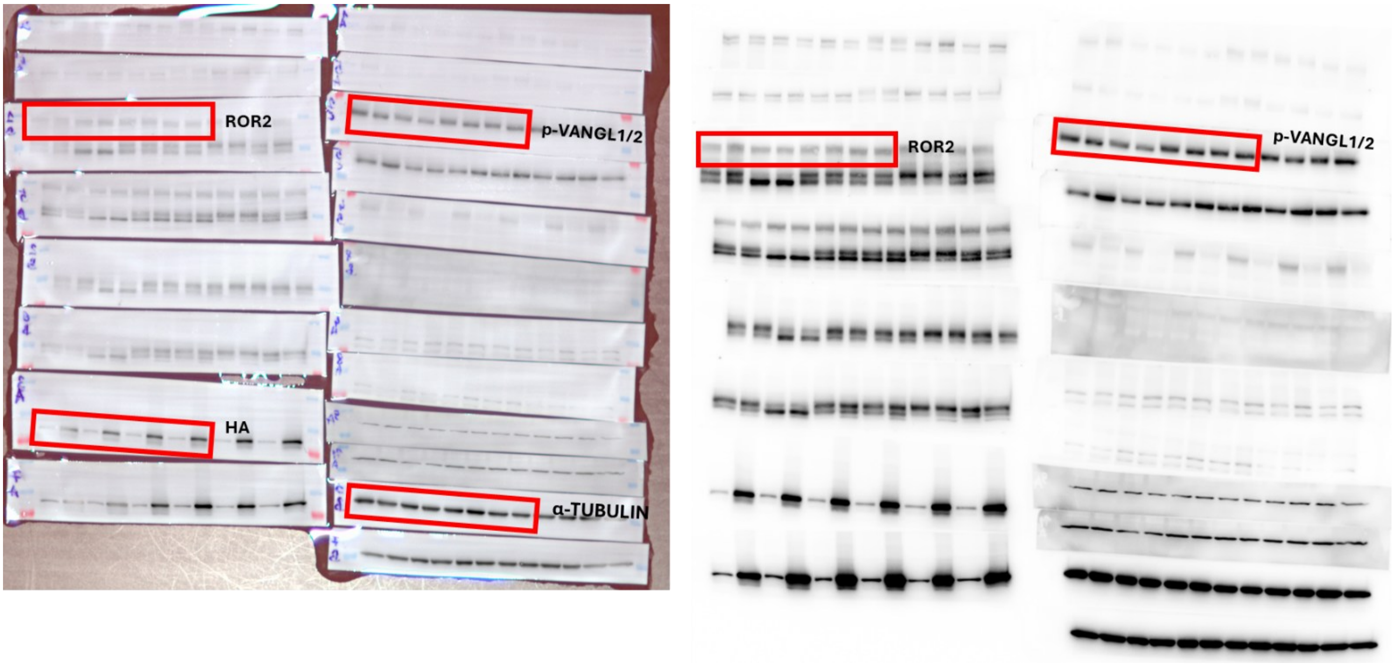

Supplementary Figure 7

continued

Fig. 3f

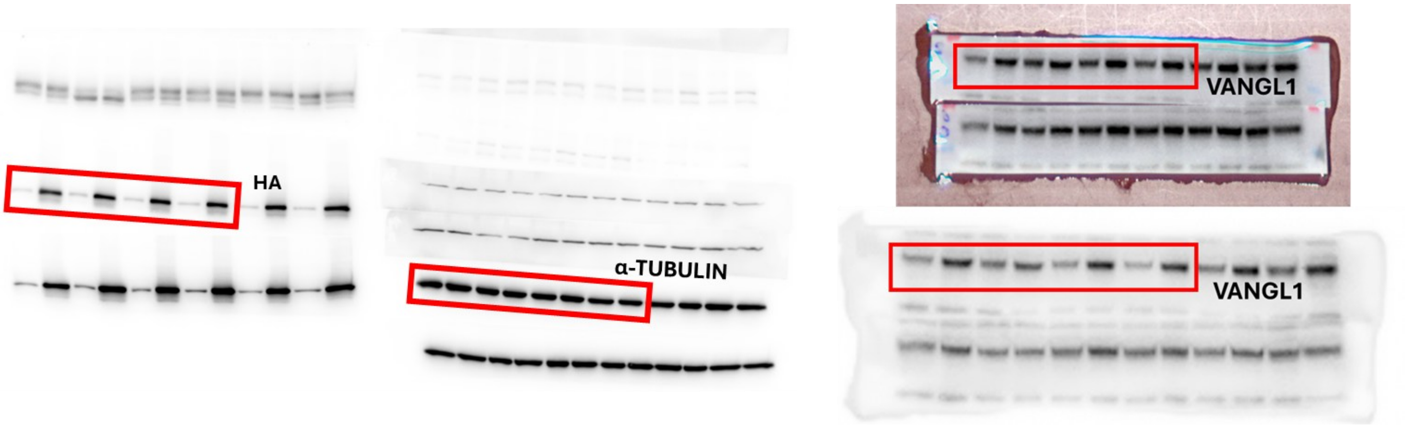

Fig. 4a

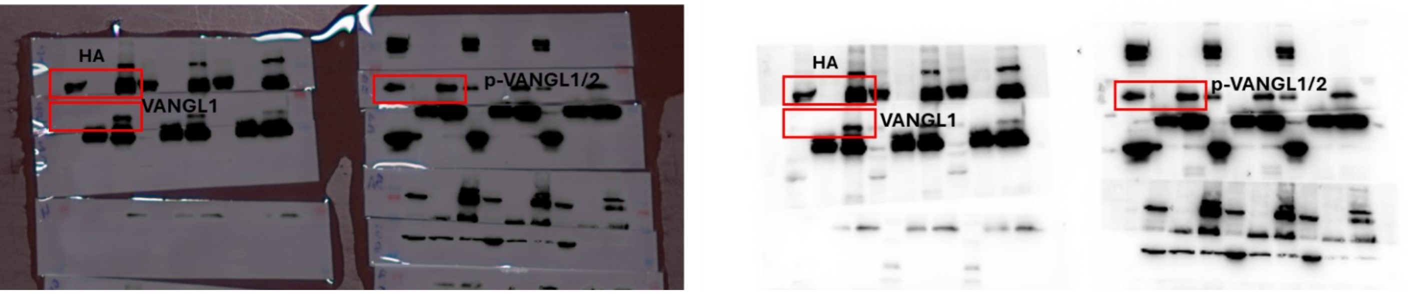

Fig. 4d

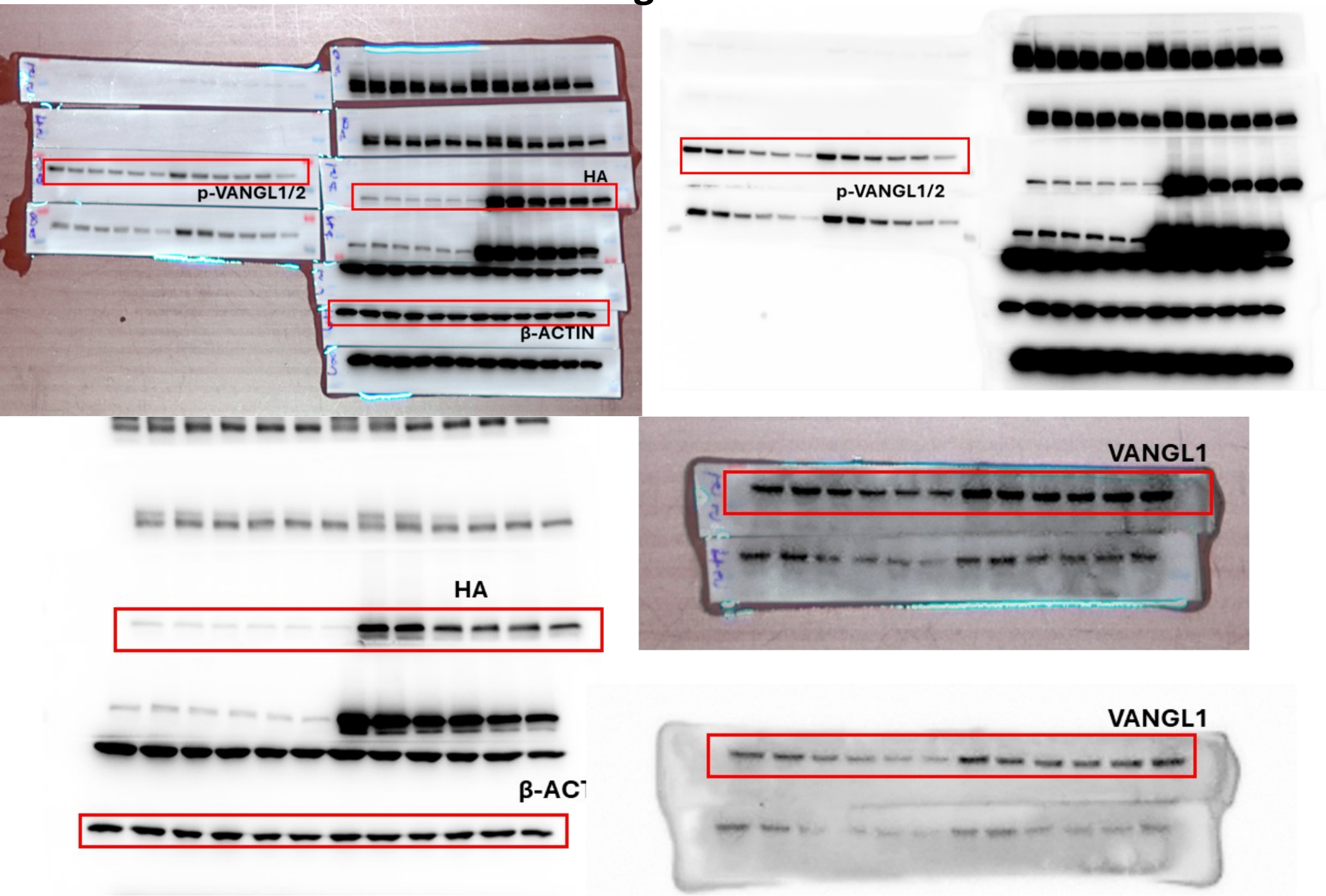

Supplementary Figure 7

continued

Fig. 4g

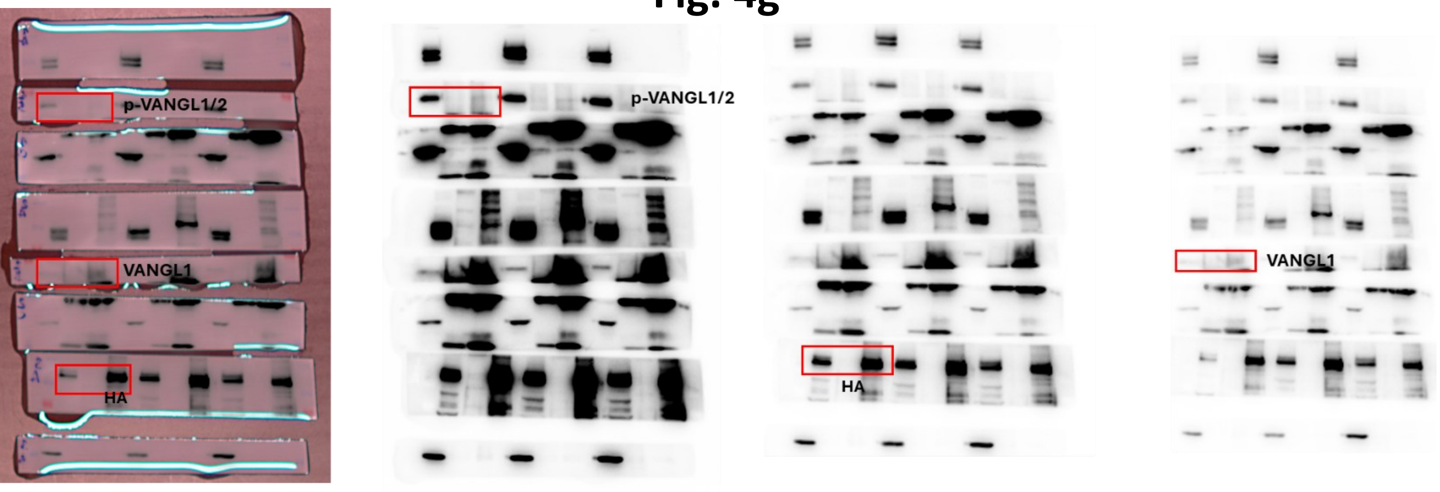

Fig. 4j

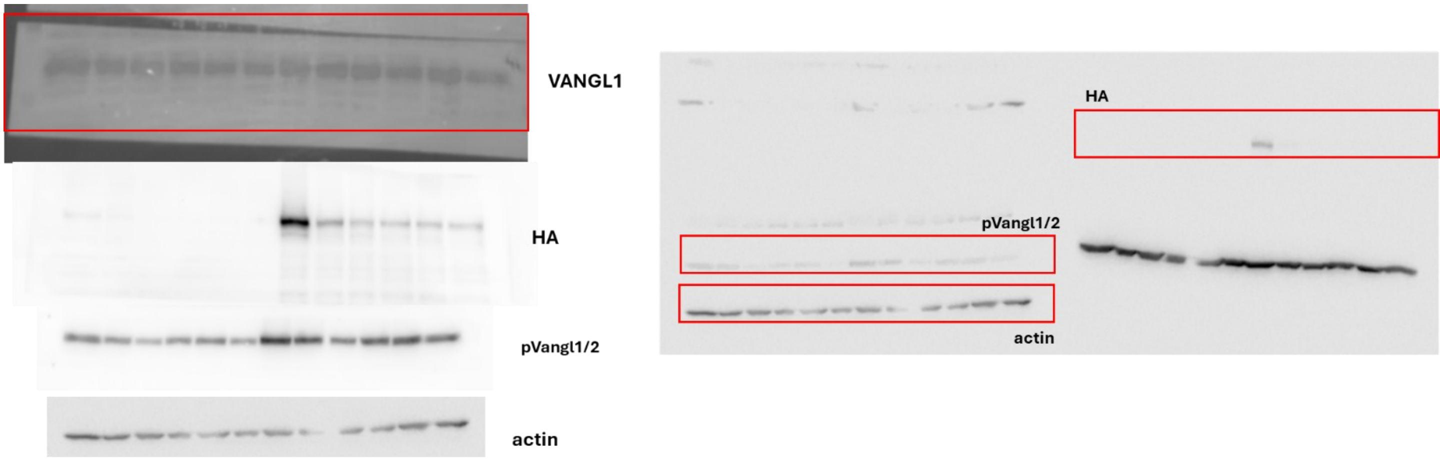

Fig. 4m

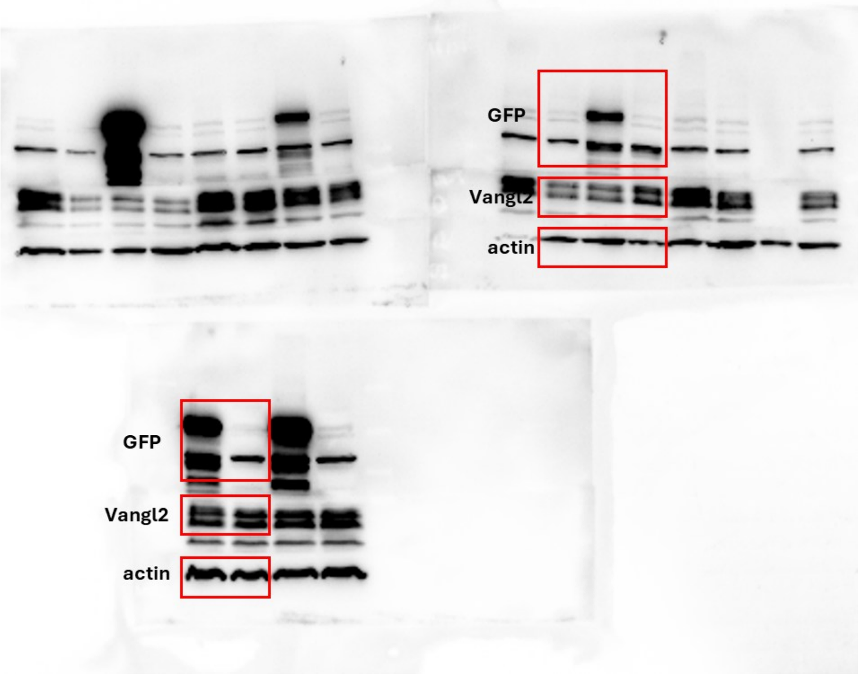

Supplementary Figure 7

continued

Fig. 5b

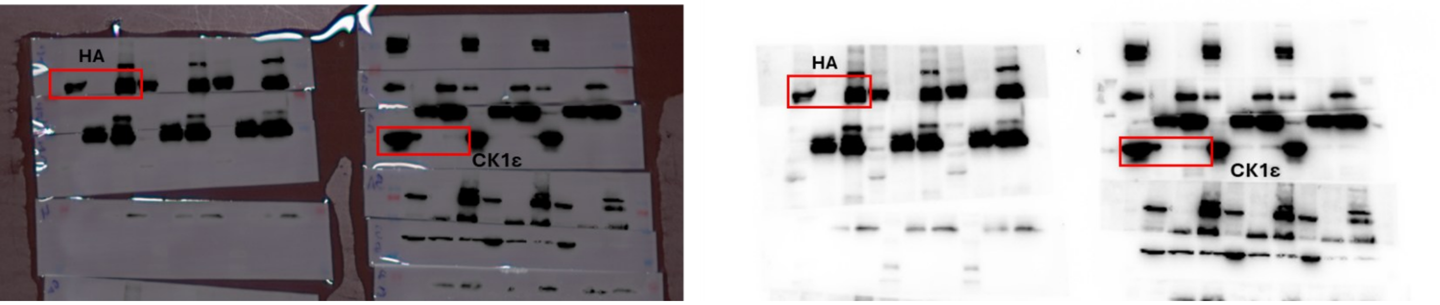

Fig. 5d

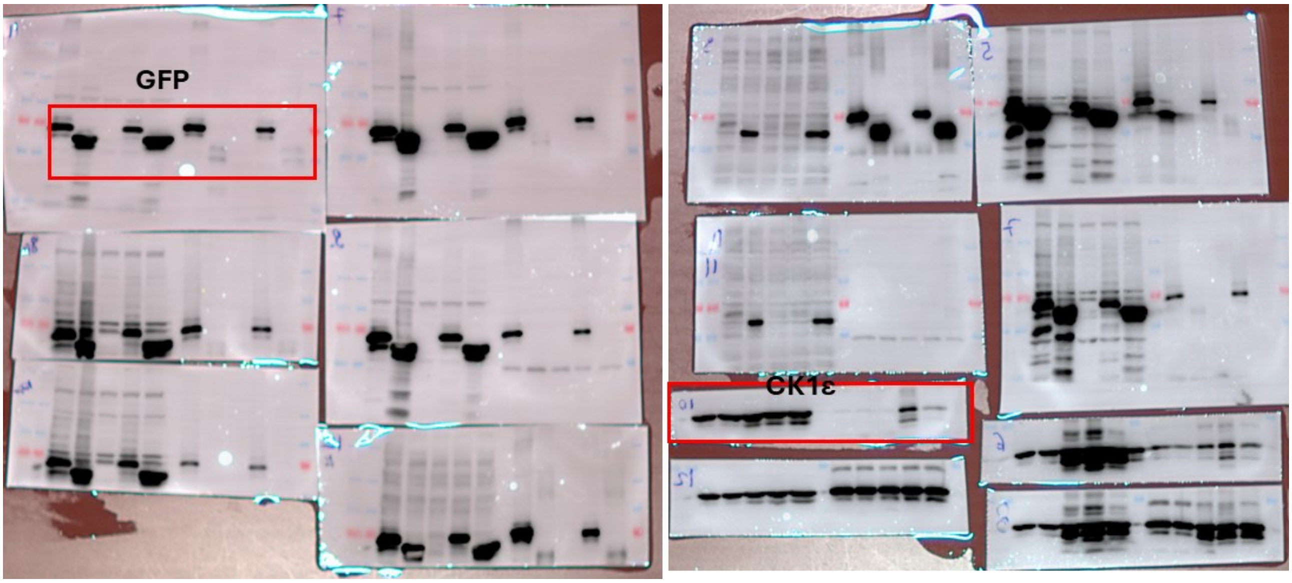

Fig. 5e

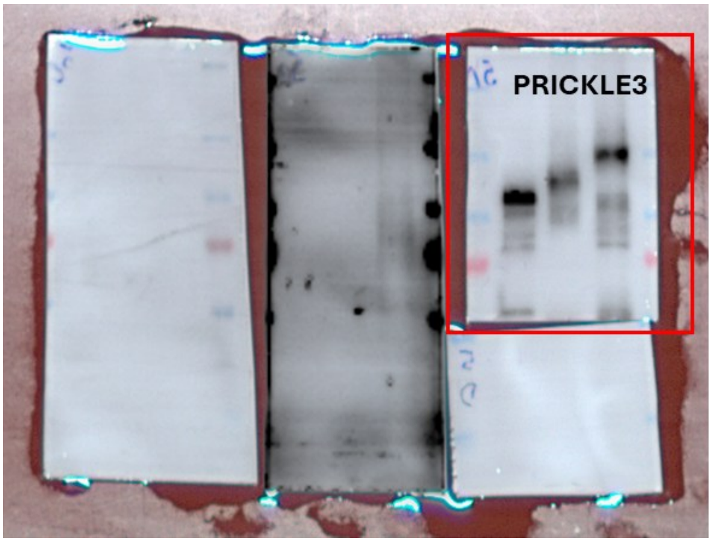

Supplementary Figure 7
continued

Fig. 5f

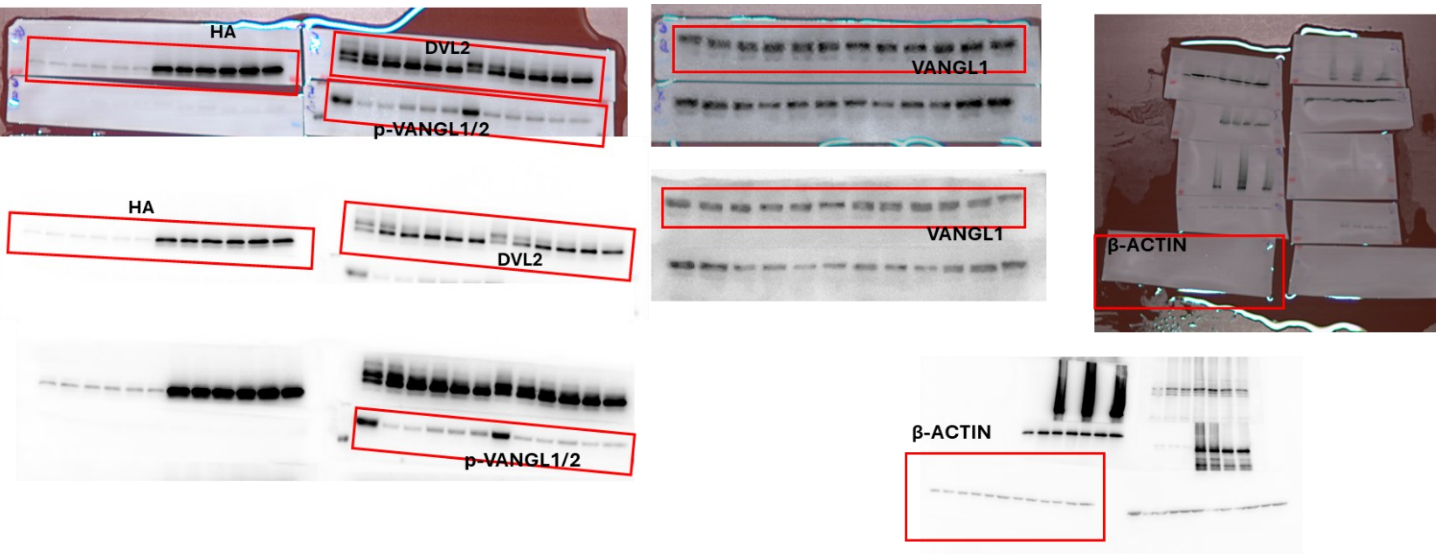

Fig. 6a

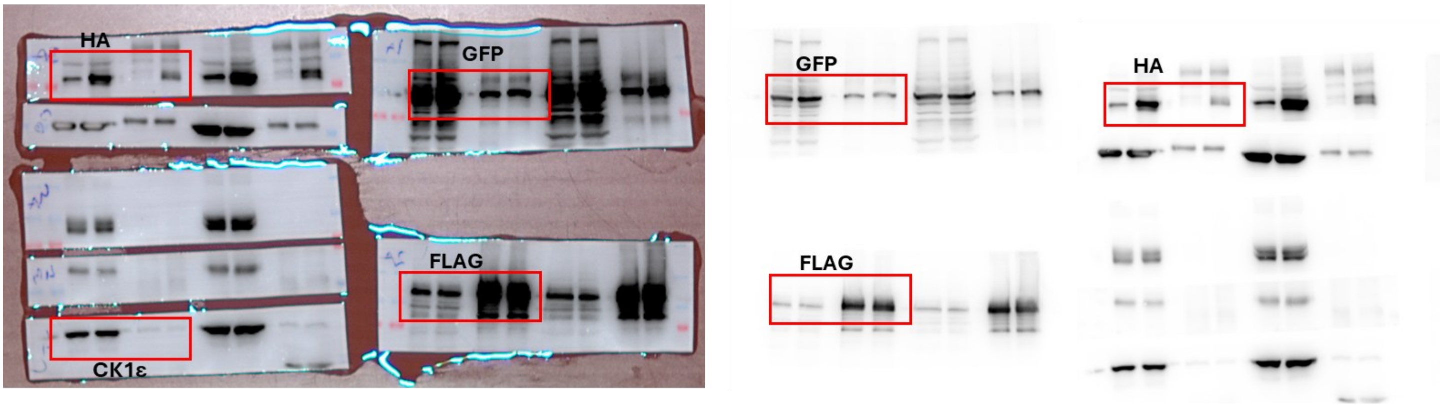

Fig. 6c

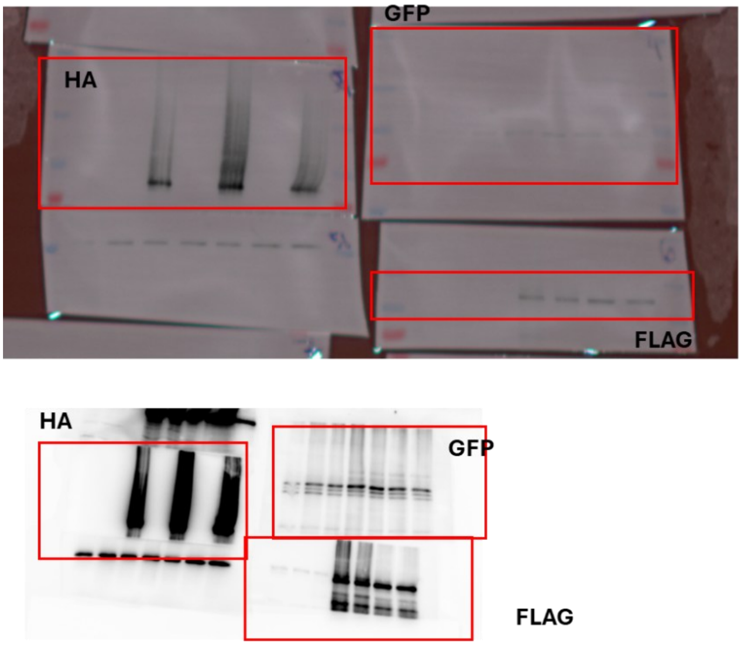

Supplementary Figure 7
continued

Fig. 6f

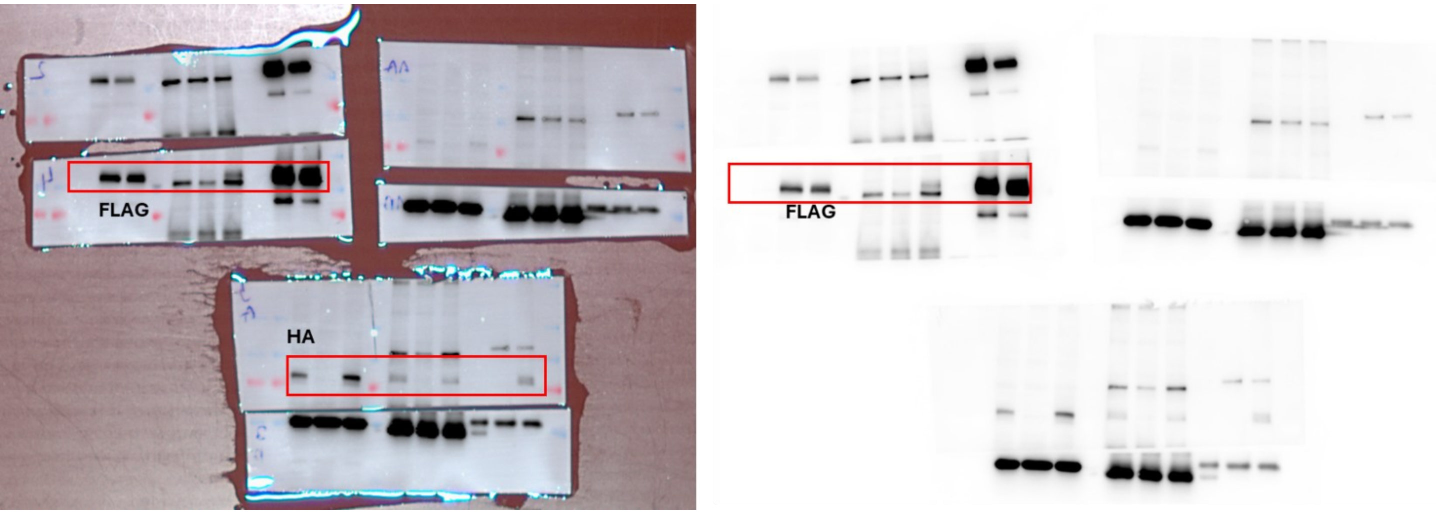

Fig. 6h

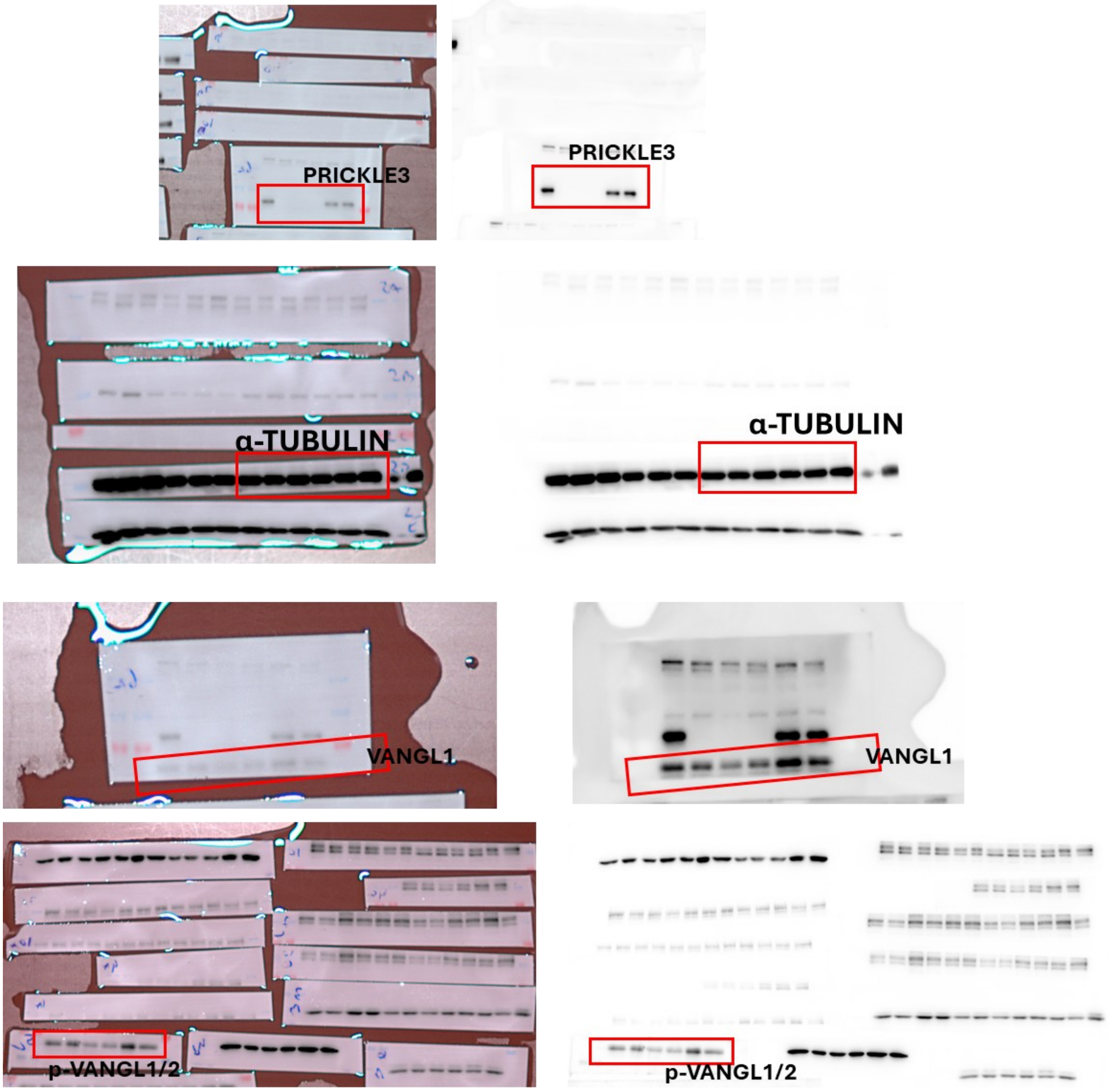

Supplementary Figure 7

continued

Fig. 6j

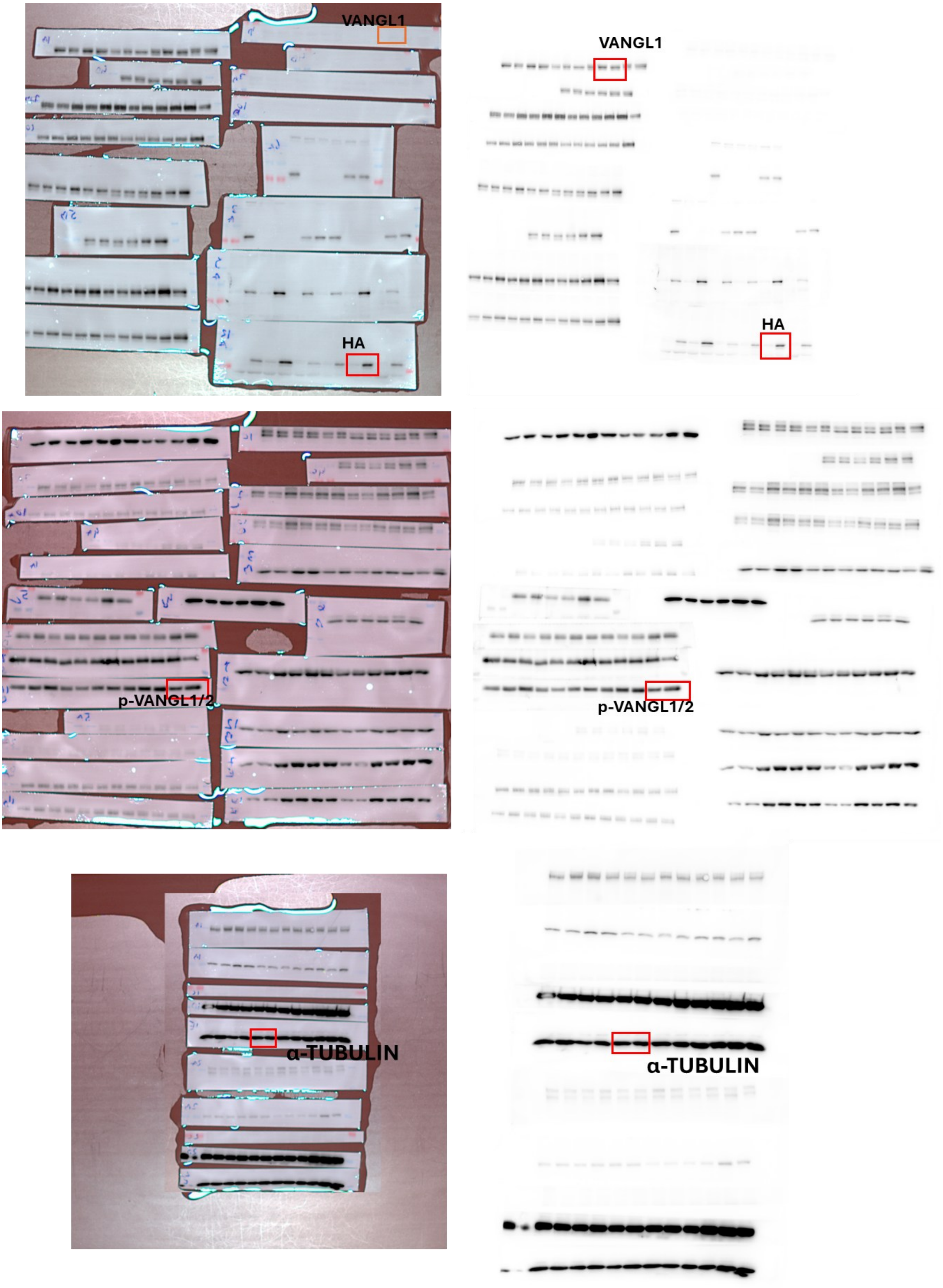

Supplementary Table 1

Overview of cell lines used in this study

Cell line	Source	Additional information
HEK293 T-REx	(R71007, Invitrogen)	Wild type cells
HEK293	(CRL-1573, ATTC)	Wild type cells
HEK293 T-REx PRICKLE1 TetON	This publication	Cells that inducibly overexpress PRICKLE1
HEK293 T-REx PRICKLE3 TetON	This publication	Cells that inducibly overexpress PRICKLE3
HEK293 T-REx miniTurboID TetON	This publication	miniTurboID cell line
HEK293 T-REx miniTurboID PRICKLE1 TetON	This publication	miniTurboID cell line
HEK293 T-REx miniTurboID PRICKLE2 TetON	This publication	miniTurboID cell line
HEK293 T-REx miniTurboID PRICKLE3 TetON	This publication	miniTurboID cell line
HEK293 VANGL1/2 KO	Mentink R.A. et al., 2018	Cells lacking VANGL1/2; CRISPR/Cas9
HEK293 VANGL2 KO	Mentink R.A. et al., 2018	Cells lacking VANGL2; CRISPR/Cas9
HEK293 PRICKLE3 KO	This publication	Cells lacking PRICKLE3; CRISPR/Cas9
HEK293 RNF43/ZNRF3 KO	Radaszkiewicz T., et al., 2020	Cells lacking RNF43/ZNRF3; CRISPR/Cas9
PRICKLE3 TetON MDCK	This publication	Cells that inducibly overexpress PRICKLE3
PRICKLE3 TetON RNF43 KO	This publication	Cells that inducibly overexpress PRICKLE3
MDCK wt	ATCC-CCL-34 MDCK (NBL-2)	Wild type cells

Supplementary Table 2

Overview of plasmid constructs used in this study

Plasmid	Source	Additional information
pcDNA4-HA-PRICKLE3	This publication	Inducible expression of PRICKLE3; stable cell lines generation
pcDNA4-HA-PRICKLE1	This publication	Inducible expression of PRICKLE1; stable cell lines generation
pcDNA4-CTRL-miniTurboBioID	This publication	MiniTurboID stable cell lines generation
pcDNA4-PRK1-miniTurboBioID	This publication	MiniTurboID stable cell lines generation
pcDNA4-PRK2-miniTurboBioID	This publication	MiniTurboID stable cell lines generation
pcDNA4-PRK3-miniTurboBioID	This publication	MiniTurboID stable cell lines generation
pcDNA3-RNF43-FLAG	This publication	Expression of RNF43 with FLAG tag
pcDNA3-RNF43 Ring mut-FLAG	This publication	Expression of RNF43 mutant with FLAG tag
pCW57-EPHA2-V5-miniTurboID	kindly provided by Vítězslav Bryja	backbone for cloning
pCMV6-XL5-Prickle1	Origene	backbone for cloning
pGateway 3XFlag Prickle2	Addgene	backbone for cloning
pGFP_LMO6 (PRICKLE3)	kindly provided by Susana de la Luna	backbone for cloning
myc-Vangl1	Belotti et al., 2012	Expression of VANGL1 with myc tag
GFP-Vangl2	Belotti et al., 2012	Expression of VANGL2 with GFP tag
His-ubiquitin	Tauriello et al., 2010	Tagged ubiquitin for His-Ub pulldown assay
pcDNA4-TO-RNF43-2xHA-2xFLAG	Koo et al., 2012	backbone for cloning
pcDNA4-TO-RNF43Mut1-2xHA-2xFLAG	Koo et al., 2012	backbone for cloning
pcDNA3-Ck1ε	Foldynová-Trantírková S. et al., 2010	Expression of CK1ε
pcDNA3	Invitrogen	backbone for cloning
hRNF43	Tsukiyama et al., 2015	Expression of RNF43 with HA tag
V5-Pk1	This publication	Expression of PRICKLE1 with V5 tag
V5-Pk2	This publication	Expression of PRICKLE1 with V5 tag
V5-Pk3	This publication	Expression of PRICKLE1 with V5 tag
Pk3 aa 526-615 recombinant plasmid	This publication	For recombinant protein purification
Vangl2 aa 1-100 recombinant plasmid	This publication	For recombinant protein purification
pCS2+ Ck1e recombinant plasmid	Harnos et al., 2019	For recombinant protein purification
pCS2+-mApple-vangl2	Le Y, et al., 2025	Expression of VANGL2 with GFP tag
pCS105-EGFP-Prickle	Le Y, et al., 2025	Expression of Prickle with GFP tag
Topflash	Korinek et al., 1997	Reporter for WNT activity
Renilla	Korinek et al., 1997	Reporter for houskeeping gene transcription
pGFP_LMO6 (PRICKLE3) aa 1-375	This publication	Expression of N-terminal PRICKLE3
pGFP_LMO6 (PRICKLE3) aa 372-615	This publication	Expression of C-terminal PRICKLE3

Supplementary Table 3

Overview of antibodies used in this study

Antibody	Source	Additional information
β-actin (rabbit monoclonal)	Cell Signaling Technology, CS-4970	WB (1:2000)
DVL-2 (rabbit polyclonal)	Cell Signaling Technology, CS-3216	WB (1:1000)
DVL-3 (mouse monoclonal)	Santa Cruz Biotechnology, SC-8027	WB (1:1000)
LRP6 (rabbit monoclonal)	Cell Signaling Technology, CS-2560	WB (1:1000)
pLRP6 (rabbit polyclonal)	Cell Signaling Technology, CS-2568	WB (1:1000)
α- TUBULIN (monoclonal mouse)	Sigma-Aldrich, T6793	WB (1:2000)
ROR2 (mouse monoclonal)	Santa Cruz Biotechnology, SC-374174	WB (1:1000)
CK1ε (mouse monoclonal)	BD Biosciences, 610446	WB (1:1000); IF (1:500); IP (1 µg)
CK1ε (goat polyclonal)	Santa Cruz Biotechnology, SC-6471	IF (1:500)
pVANGL1/2 (rabbit monoclonal)	ABclonal, AP1206	WB (1:1000), IF (1:500)
VANGL1 (monoclonal mouse)	Santa Cruz, SC-166844	WB (1:500)
HA-11 (mouse monoclonal)	Covance, MMS-101R	WB (1:2000); IF (1:500); IP (1 µg)
HA (rabbit polyclonal)	Abcam, ab9110	WB (1:2000); IF (1:500); IP (1 µg)
HA (goat polyclonal)	Bethyl Laboratories, A190-138A	IF (1:500)
c-Myc (rabbit polyclonal)	Sigma-Aldrich, C3956	WB (1:1000); IF (1:500)
GFP 3 H9 (rat monoclonal)	Chromotek, 3 H9	IP (1 µg)
GFP (rabbit polyclonal)	Fitzgerald, 20R-GR-011	WB (1:2000); IP (1 µg)
FLAG M2 (mouse monoclonal)	Sigma-Aldrich, F3165	WB (1:2000), IF (1:500), IP (1 µg)
FLAG (rabbit polyclonal)	Sigma, F7425	WB (1:2000); IF (1:500), IP (1 µg)
V5 (mouse monoclonal)	Thermo Fisher Scientific, R96025	WB (1:1000), IF (1:1000); IP (1 µg)
PRICKLE3 (rabbit polyclonal)	Atlas Antibodies, HPA000998	WB (1:1000)
pSer/pThr (mouse monoclonal)	BD Biosciences, 612548	WB (1:500)
Streptavidin, Alexa Fluor 488 conjugate	Thermo Fisher Scientific, S-32354	IF (1:600)
a-mouse-HRP (goat monoclonal)	Sigma-Aldrich, A4416	WB (1:5000)
a-rabbit-HRP (goat monoclonal)	Sigma-Aldrich, A0545	WB (1:5000)
a-mouse Alexa Fluor 488 (donkey polyclonal)	Thermo Fisher Scientific, A21202	IF (1:600)
a-goat Alexa Fluor 488 (donkey polyclonal)	Thermo Fisher Scientific, A11055	IF (1:600)
a-rabbit Alexa Fluor 568 (donkey polyclonal)	Thermo Fisher Scientific, A10042	IF (1:600)
a-mouse Alexa Fluor 680 (donkey polyclonal)	Thermo Fisher Scientific, A32788	IF (1:600)
Vangl2 (rabbit polyclonal)	Montcouquiol et al., 2006	WB (1:1000)
