# Supplementary material for: PRICKLE3 protects VANGL proteins from CK1-mediated phosphorylation and RNF43-mediated degradation
Source: Commun Biol. 2025 Dec 27;9:142. doi: 10.1038/s42003-025-09422-9 (PMC12859043; doi:10.1038/s42003-025-09422-9)
Supplement: Supplementary file 2 — Description of Additional Supplementary Materials [file 42003_2025_9422_MOESM2_ESM.pdf]

Description of Additional Supplementary Files

File name: Supplementary Data 1

Description: Table with log2 transformed, normalized and imputed MaxLFQ protein intensities, results of limma test for each PRICKLE-control comparison.

File name: Supplementary Data 2

Description: Upregulated preys for individual PRICKLE isoforms.

File name: Supplementary Data 3

Description: UpSet plot background table

File name: Supplementary Data 4

Description: Human Cell Map localization of individual preys. Columns starting with "Up_" denote whether the protein was upregulated (1) or not (0) in particular PRICKLE-control comparison.

File name: Supplementary Data 5

Description: Interactors specific to the individual PRICKLE proteins

File name: Supplementary Data 6

Description: Metascape analysis of interactors specific to PRICKLE1

File name: Supplementary Data 7

Description: Metascape analysis of interactors specific to PRICKLE2

File name: Supplementary Data 8

Description: Metascape analysis of interactors specific to PRICKLE3

File name: Supplementary Data 9

Description: REPRINT results

File name: Supplementary Data 10

Description: Transitions list

File name: Supplementary Data 11

Description: The source data in the Excel format behind the graphs in the paper
