# Supplementary material for: PRICKLE3 protects VANGL proteins from CK1-mediated phosphorylation and RNF43-mediated degradation
Source: Commun Biol. 2025 Dec 27;9:142. doi: 10.1038/s42003-025-09422-9 (PMC12859043; doi:10.1038/s42003-025-09422-9)
Supplement: Supplementary file 5 — Reporting Summary [file 42003_2025_9422_MOESM5_ESM.pdf]

nature portfolio

Corresponding author(s): Jakub Harnos, COMMSBIO-25-10810

Last updated by author(s): Nov 23, 2025

Reporting Summary

Nature Portfolio wishes to improve the reproducibility of the work that we publish. This form provides structure for consistency and transparency in reporting. For further information on Nature Portfolio policies, see our Editorial Policies and the Editorial Policy Checklist.

Statistics

For all statistical analyses, confirm that the following items are present in the figure legend, table legend, main text, or Methods section.

n/a	Confirmed	
☐	☒	The exact sample size (n) for each experimental group/condition, given as a discrete number and unit of measurement
☐	☒	A statement on whether measurements were taken from distinct samples or whether the same sample was measured repeatedly
☐	☒	The statistical test(s) used AND whether they are one- or two-sided<br>*Only common tests should be described solely by name; describe more complex techniques in the Methods section.*
☒	☐	A description of all covariates tested
☒	☐	A description of any assumptions or corrections, such as tests of normality and adjustment for multiple comparisons
☐	☒	A full description of the statistical parameters including central tendency (e.g. means) or other basic estimates (e.g. regression coefficient) AND variation (e.g. standard deviation) or associated estimates of uncertainty (e.g. confidence intervals)
☐	☒	For null hypothesis testing, the test statistic (e.g. F, t, r) with confidence intervals, effect sizes, degrees of freedom and P value noted<br>*Give P values as exact values whenever suitable.*
☒	☐	For Bayesian analysis, information on the choice of priors and Markov chain Monte Carlo settings
☒	☐	For hierarchical and complex designs, identification of the appropriate level for tests and full reporting of outcomes
☒	☐	Estimates of effect sizes (e.g. Cohen's d, Pearson's r), indicating how they were calculated

Our web collection on statistics for biologists contains articles on many of the points above.

Software and code

Policy information about availability of computer code

Data collection	Data acquisition. Mass spectrometry data were acquired in data-independent acquisition (DIA) mode. Survey scans covered m/z 350–1,400 at a resolution of 60,000 (at m/z 200) with a maximum injection time of 55 ms and a normalized AGC target of 300%. HCD MS/MS spectra were acquired at 27% normalized collision energy in the m/z 200–2,000 range at a resolution of 30,000 (maximum injection time 55 ms; normalized AGC target 1,000%). An overlapping-window DIA scheme covering precursor m/z 400–800 was used (window placements listed in Supplementary Table S13).<br><br>Data conversion. Raw files were converted to mzML using msconvert (v. 3.0.21193-ccb3e0136) applying vendor peak picking (msLevel = 1–) and demultiplexing (optimization = overlap_only; massError = 10 ppm).<br><br>Database search. mzML files were processed with DIA-NN (v. 1.8.1; Demichev et al., 2020) in library-free mode using a modified cRAP database (111 sequences) and the UniProtKB Homo sapiens reference proteome (UP000005640, release 2023-11-08; 20,596 sequences). Carbamidomethylation (C) was set as a fixed modification; Trypsin/P was selected as the protease (1 missed cleavage allowed; peptide length 7–30 aa). False discovery rate was controlled at 1%. MS1 and MS2 accuracies (8 ppm and 21 ppm, respectively) and scan-window settings (9 scans) were set based on initial test searches. Match-between-runs (MBR) was enabled.<br><br>Quantification and initial processing. MaxLFQ intensities from DIA-NN (report.tsv) were processed within the containerized workflow environment provided at https://github.com/OmicsWorkflows
Data analysis	Software environment. All downstream mass spectrometry data analyses were performed in R (v. 4.4.0). Analysis code and reproducible scripts are publicly available at https://github.com/HarnosLab/2024_Radaszkiewicz

Data preprocessing. As implemented in the referenced workflow, protein group intensities were log2-transformed and normalized using LoessF. Missing values were imputed column-wise from a left-shifted Gaussian distribution (shift = 1.8, scale = 0.3). Differential expression analysis was performed using the LIMMA statistical framework.

Visualization and functional analysis. Data visualization used the R packages ComplexHeatmap, UpSetR, and ggplot2. Functional enrichment analyses were carried out using gProfiler2 (version e111_eg58_p18_f463989d) and the Metascape platform.
Protein–protein interaction scoring. SAINT probability scores for bait–prey interactions were computed using REPRINT (v. 2.0; Mellacheruvu et al., 2013) with experiment type set to "Proximity-dependent Biotinylation," file type set to tab-separated matrix, and only user-provided controls selected. SAINTexpress was used to compute probabilistic scores. Dot plots were generated using the ProHits-viz suite (Knight et al., 2017).

GraphPad Prism software was used for statistical analysis and data graphical representation. SuperPlotsOfData - Plots Data and its Replicates - web applicaiton was used for statistical analysis and data graphical representation. ImageJ was used for confocal images analysis. LasX software was used for confocal images analysis.

For manuscripts utilizing custom algorithms or software that are central to the research but not yet described in published literature, software must be made available to editors and reviewers. We strongly encourage code deposition in a community repository (e.g. GitHub). See the Nature Portfolio guidelines for submitting code & software for further information.

Data

Policy information about availability of data

All manuscripts must include a data availability statement. This statement should provide the following information, where applicable:

- Accession codes, unique identifiers, or web links for publicly available datasets
- A description of any restrictions on data availability
- For clinical datasets or third party data, please ensure that the statement adheres to our policy

The mass spectrometry proteomics data have been deposited to the ProteomeXchange Consortium via the PRIDE partner repository with the dataset identifier PXD057854.

Research involving human participants, their data, or biological material

Policy information about studies with human participants or human data. See also policy information about sex, gender (identity/presentation), and sexual orientation and race, ethnicity and racism.

Reporting on sex and gender	Not applicable
Reporting on race, ethnicity, or other socially relevant groupings	Not applicable
Population characteristics	Not applicable
Recruitment	Not applicable
Ethics oversight	Not applicable

Note that full information on the approval of the study protocol must also be provided in the manuscript.

Field-specific reporting

Please select the one below that is the best fit for your research. If you are not sure, read the appropriate sections before making your selection.

☒ Life sciences ☐ Behavioural & social sciences ☐ Ecological, evolutionary & environmental sciences

For a reference copy of the document with all sections, see nature.com/documents/nr-reporting-summary-flat.pdf

Life sciences study design

All studies must disclose on these points even when the disclosure is negative.

Sample size	Sample sizes were determined based on prior published work in the field and pilot data collected for this study.
Data exclusions	No data were excluded in this study.
Replication	All experiments were replicated at least three times independently with consistent results. The reported n values represent biological replicates; N marks the number of individual subjects analyzed. An exception is the experiment analyzing Prickle3 phosphorylation (Fig. 5E), which was performed once but subsequently confirmed by a more detailed experiment (XXX).

Randomization	Animals were randomly assigned to experimental groups prior to treatment. For cell-based experiments, treatments were applied in a randomized manner across wells and plates. For immunofluorescence analyses, images of fields were acquired randomly to avoid selection bias, and the cells included in the analysis were also selected at random.
Blinding	Investigators performing data analysis (e.g., quantification of Western blot bands and fluorescence staining counts) were blinded to group allocation whenever feasible.

Reporting for specific materials, systems and methods

We require information from authors about some types of materials, experimental systems and methods used in many studies. Here, indicate whether each material, system or method listed is relevant to your study. If you are not sure if a list item applies to your research, read the appropriate section before selecting a response.

Materials & experimental systems

n/a	Involved in the study	
☐	☒	Antibodies
☐	☒	Eukaryotic cell lines
☒	☐	Palaeontology and archaeology
☐	☒	Animals and other organisms
☒	☐	Clinical data
☒	☐	Dual use research of concern
☒	☐	Plants

Methods

n/a	Involved in the study	
☒	☐	ChIP-seq
☒	☐	Flow cytometry
☒	☐	MRI-based neuroimaging

Antibodies

Antibodies used	All primary and secondary antibodies used in this study are listed in Supplementary Table S12 with catalog numbers.
Validation	All antibodies were validated for specificity using at least one of the following: Knockout/over-expression or literature review. Antibodies were tested in at least two independent experiments. Dilutions were optimized for each assay (Western blot, immunofluorescence, immunoprecipitation) to produce minimal background and robust signal.

Eukaryotic cell lines

Policy information about cell lines and Sex and Gender in Research

Cell line source(s)	All cell lines and their sources are described in the Supplementary Table 10.
Authentication	All cell lines were authenticated by short tandem repeat (STR) profiling. Authentication was performed by the institutional genomics core facility within the past 12 months, and results matched the reference profiles from ATCC/ECACC.
Mycoplasma contamination	All cell lines tested negative for mycoplasma contamination, verified by PCR. Mycoplasma testing is conducted routinely every 4–6 weeks
Commonly misidentified lines (See ICLAC register)	None used.

Animals and other research organisms

Policy information about studies involving animals; ARRIVE guidelines recommended for reporting animal research, and Sex and Gender in Research

Laboratory animals	Zebrafish (Danio rerio); African clawed frog (Xenopus laevis) - only early embroynic stages, which are not considered animals under current legislation, were used in the study
Wild animals	This research does not involve wild animals.
Reporting on sex	No sex-specific animals were selected for the experiments.
Field-collected samples	This research does not involve field-collected samples.
Ethics oversight	All experiments involving zebrafish were conducted in accordance with protocol BR22-1497 as approved by the Institutional Animal Care and Use Committee (IACUC) of the National University of Singapore. All procedures involving Xenopus laevis were conducted in accordance with Czech legislation on the use of animals for research and were approved by the relevant institutional and governmental authorities (MSMT-30784/2022-1; Animal Care and Housing Approval: 45055/2020-MZE-18134, Ministry of Agriculture of the Czech Republic).

Note that full information on the approval of the study protocol must also be provided in the manuscript.

Plants

Seed stocks	*Report on the source of all seed stocks or other plant material used. If applicable, state the seed stock centre and catalogue number. If plant specimens were collected from the field, describe the collection location, date and sampling procedures.*
Novel plant genotypes	*Describe the methods by which all novel plant genotypes were produced. This includes those generated by transgenic approaches, gene editing, chemical/radiation-based mutagenesis and hybridization. For transgenic lines, describe the transformation method, the number of independent lines analyzed and the generation upon which experiments were performed. For gene-edited lines, describe the editor used, the endogenous sequence targeted for editing, the targeting guide RNA sequence (if applicable) and how the editor was applied.*
Authentication	*Describe any authentication procedures for each seed stock used or novel genotype generated. Describe any experiments used to assess the effect of a mutation and, where applicable, how potential secondary effects (e.g. second site T-DNA insertions, mosiacism, off-target gene editing) were examined.*
